# Mapping Determinants of Gene Expression Plasticity by Genetical Genomics in *C. elegans*

**DOI:** 10.1371/journal.pgen.0020222

**Published:** 2006-12-29

**Authors:** Yang Li, Olga Alda Álvarez, Evert W Gutteling, Marcel Tijsterman, Jingyuan Fu, Joost A. G Riksen, Esther Hazendonk, Pjotr Prins, Ronald H. A Plasterk, Ritsert C Jansen, Rainer Breitling, Jan E Kammenga

**Affiliations:** 1 Groningen Bioinformatics Centre, Groningen Biomolecular Sciences and Biotechnology Institute, University of Groningen, Haren, The Netherlands; 2 Laboratory of Nematology, Wageningen University, Wageningen, The Netherlands; 3 Hubrecht Laboratory, Netherlands Institute of Developmental Biology, Utrecht, The Netherlands; North Carolina State University, United States of America

## Abstract

Recent genetical genomics studies have provided intimate views on gene regulatory networks. Gene expression variations between genetically different individuals have been mapped to the causal regulatory regions, termed expression quantitative trait loci. Whether the environment-induced plastic response of gene expression also shows heritable difference has not yet been studied. Here we show that differential expression induced by temperatures of 16 °C and 24 °C has a strong genetic component in Caenorhabditis elegans recombinant inbred strains derived from a cross between strains CB4856 (Hawaii) and N2 (Bristol). No less than 59% of 308 *trans*-acting genes showed a significant eQTL-by-environment interaction, here termed plasticity quantitative trait loci. In contrast, only 8% of an estimated 188 *cis*-acting genes showed such interaction. This indicates that heritable differences in plastic responses of gene expression are largely regulated in *trans*. This regulation is spread over many different regulators. However, for one group of *trans*-genes we found prominent evidence for a common master regulator: a transband of 66 coregulated genes appeared at 24 °C. Our results suggest widespread genetic variation of differential expression responses to environmental impacts and demonstrate the potential of genetical genomics for mapping the molecular determinants of phenotypic plasticity.

## Synopsis

It is widely documented that environmental changes will induce differential expression of genes, yet it is unknown how these patterns of environment-induced expression plasticity are inherited and how they differ between genetically divergent individuals of a biological species. In this paper the authors used recombinant inbred lines of the nematode worm C. elegans that were derived from parental lines originally collected in Bristol (United Kingdom) and Hawaii, and measured genome-wide gene expression at two different temperatures. Using statistical analysis tools developed for quantitative trait locus mapping, they found genes with genetically determined differences in their plastic response to temperature changes. A majority of them were found to be regulated by genes at a different genome position (regulated in *trans*). A striking observation was a group of 66 genes that share a common potential regulator and may be related to differences in fertility plasticity. These results show that differential responses of different genotypes to environmental changes are widespread. Because all species are subjected to environmental change, both at individual and evolutionary time scales, the authors' work calls for studying the heritable component of plasticity of gene regulation in other organisms to enhance understanding of the environmental forces that drive evolutionary adaptation.

## Introduction

Expression quantitative trait loci (eQTLs) are polymorphic genetic loci that cause heritable differences in mRNA concentration. eQTLs have been used in recent genetical genomics studies [1] to infer the structure of genome-wide gene regulatory networks [2–4]. The definition of eQTLs in these studies is essentially static and does not consider the highly dynamic nature of gene expression. However, mRNA levels respond rapidly to variable ambient conditions such as temperature change. This has been shown for yeast [5], bacteria [6], and C. elegans [7] after exposure to heat shock.

In contrast to these short-term exposures to extreme temperatures, populations under natural conditions are often exposed to longer periods of less extreme temperature changes. The ability to respond to these temperature changes (so-called phenotypic plasticity) differs among genotypes. Phenotypic plasticity to temperature plays an important role in the evolution of life histories in a variable climate [8] and is widespread among species. Typical examples are temperature-induced sex determination in reptiles [9] and seasonal polyphenism in butterflies [10]. The detection of temperature-specific proteins was reported by Madi et al. [11], who analyzed proteome temperature plasticity in wild-type C. elegans.

Insight into the genetic control of plasticity is a key issue for understanding evolutionary trajectories. Recently, we detected specific QTLs underlying plasticity to temperature in C. elegans life-history traits such as growth and fertility [12].

In this paper we focus on the plasticity of gene expression in C. elegans juveniles that have been exposed for their entire life to (different) constant temperatures. We used a genetical genomics approach for detecting loci controlling such gene expression plasticity (plasticity quantitative trait loci [pQTL]). It has been shown that intraspecific evolution of variations in gene expression is to a large extent dominated by intense stabilizing selection [13]. This implies that any beneficial mutation affecting gene expression levels should show its favorable effects selectively in certain environments without disrupting the existing adaptation to other conditions. This is much more likely the case for pQTLs than for nonplastic eQTLs. The “genotype-by-environment” interaction characterizing a pQTL is the prerequisite for adaptive evolution in a fluctuating environment [14]. In fact, it has been shown that more than half of the regulatory connections in a gene expression network are unique for specific conditions such as cell cycle, sporulation, DNA damage, and stress response [15]. Recently, genotype-by-environment interaction was found for genome-wide gene expression among yeast strains [16].

## Results/Discussion

We used a set of 80 recombinant inbred (RI) strains generated from a cross of N2 (Bristol) and CB4856 (Hawaii), representing two genetic and ecological extremes of C. elegans [17,18]. Their genetic distance amounts to about one polymorphism per 873 base pairs [19]. Both strains have contrasting behavioral phenotypes (solitary versus gregarious) [18] and differ strikingly in their response to a temperature change [12]. We have exposed the RI strains to 16 °C and 24 °C, temperatures that are known to strongly affect phenotypic characteristics such as body size, lifespan, and reproduction [12]. Gene expression patterns were assessed by oligonucleotide microarray hybridization (Genisphere) using a distant pair design, which pairs the RI strains with the largest genetic difference on the same array, to maximize the amount of useful signal for the QTL mapping [20]. The genetic architecture of the 80 RI strains and the description of a dense single nucleotide polymorphism (SNP) map can be found in Protocol S1 and Tables S1–S3.

### Genome-Wide Detection of Expression and Plasticity QTLs

**Figure 1:**
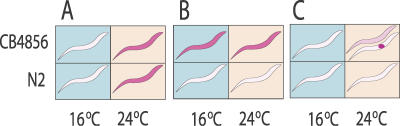
Illustration of Temperature, eQTL, and pQTL (eQTL-by-Temperature Interaction) Effects Genotype (N2 and CB4856) and temperature (16 °C and 24 °C) are two factors that might induce differential expression for transcripts. The colors of the animals correspond to the different gene expression levels. (A) Transcript with differential expression induced by temperature. The transcript is overexpressed at 24 °C independent of the genotype. (B) Transcript with strong eQTL effect. At both temperatures, worms with N2 genotype at a locus of interest show higher expression. (C) Transcript with pQTL effect. At 16 °C, transcripts show low expression in both genotypes. At 24 °C, only one allele (e.g., CB4856, as shown here) shows a strong induction of gene expression. If this upregulation is restricted to a specific tissue (the lower worm), it will be diluted in the total body when average of expression is measured (the upper worm). Other possible pQTL patterns can easily be conceived based on this example.

**Figure 2:**
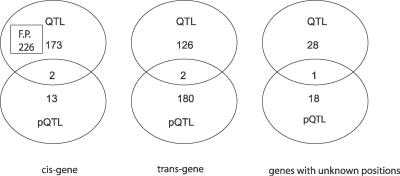
Venn Diagram Result of Joint Analysis The figures indicate the number of transcripts detected with significant *cis-* and *trans*-eQTL or pQTL effect (*p* < 0.001 with FDR of 0.04 after multiple testing correction) in a full ANOVA model (see Materials and Methods for details). In the first Venn diagram, F.P refers to the number of estimated potential false positive eQTLs.

Schematic examples of eQTL, temperature, and eQTL-by-temperature interaction (pQTL) effects are shown in Figure 1A–1C, respectively. We used a two-step procedure to detect pQTLs. First, we applied a separate eQTL analysis for the expression data at either temperature (see Materials and Methods). With a genome-wide significance threshold of 4.25 (corresponding to an effective *p*-value of 0.001; throughout the paper, thresholds are in units of −log_10_ *p*) there are 186 transcripts with significant eQTL effects at 16 °C and 279 at 24 °C, respectively (42 of these are common for both temperatures), suggesting eQTLs vary significantly between environmental conditions. To detect how much of this difference is due to pQTLs (plasticity regulators), we used the eQTL positions from the separate analyses. We postulated that interaction must happen at positions with eQTL effects and focused on these positions in a joint statistical analysis of data from both temperatures, thereby increasing the power of the method (see Materials and Methods for details). Differential expression for a given gene can result from *cis*-regulation due to variation in the region of the gene itself or from *trans*-regulation by other genes. The criterion used in our analysis is that the putative *cis*-acting QTL peak is within 2 Mb of the transcript. It is worthwhile to notice that the *cis*-QTLs could actually be *trans*-QTLs, due to the limited resolution of the mapping. We found 308 transcripts showing significant *trans*-acting eQTL effects (effective *p* < 0.001) and 182 of these (59%) showed a significant pQTL effect (eQTL-by-temperature interaction) (Figure 2). This indicates that a large part of the observed gene expression dynamics differs consistently between the two parental alleles at plasticity-controlling loci.

**Figure 3:**
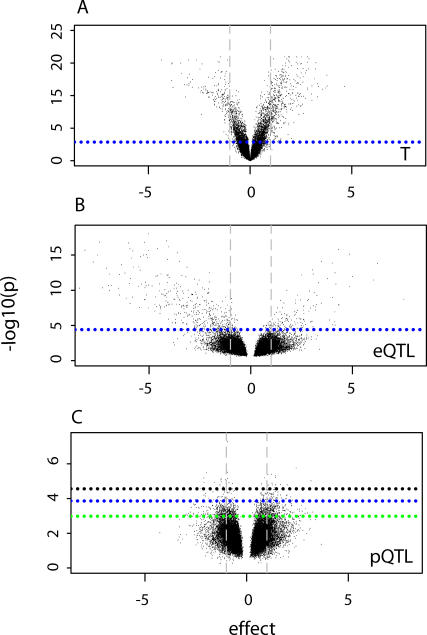
Volcano Plots for Temperature, eQTL, and pQTL Effect The temperature (T) (A), eQTL (B), and pQTL (C) effects for all genes are plotted on the *x*-axes. (A) Temperature effect −log_10_ *p*-values from intensity-based analysis are plotted on the *y*-axis. (B and C) eQTL and pQTL −log_10_ *p*-values from full model are plotted on the *y*-axes. Vertical dashed lines correspond to 2-fold change in expression. The dotted lines indicate the significance thresholds: (A) FDR 0.01; (B) *p* = 0.001 for single and two-locus search; (C) *p* = 0.001 for genomewide (black), single-locus (green), and two-locus (blue) search.

That the temperature shift indeed leads to a drastic change in the gene regulation network is confirmed by the major differential gene expression observed between the two temperatures (Figure 3A). The amount of genes with a significant eQTL is relatively small (Figure 3B), while significant pQTLs are even less common, despite their relatively large effect size (Figure 3C). This justifies our use of the powerful two-stage statistical analysis outlined above.

### Test for Genetic Assimilation

The parental lines of our RI strains originated from two very different thermal environments, and even though they have been maintained for many generations in controlled laboratory conditions, their highly divergent genomes are still expected to reflect the original allelic differences to a large extent. This gives us a unique opportunity to test our data for evidence of the controversial concept of “genetic assimilation,” whereby originally plastic traits become genetically fixed in a novel environment, e.g., because the original selective pressure favoring plasticity is no longer experienced [21]. In our case, we predict that genetic assimilation would be observed for temperature-related traits in the Hawaiian strain: genes that show strong differential expression in the highly seasonal conditions in Bristol lost this behavior in the more constant tropical oceanic climate of Hawaii. This behavior would be reflected in the alleles in our RI strains. However, we find no evidence that genetic assimilation plays a role in the observed expression patterns. Out of 182 genes with pQTL, equal numbers of genes show strong differential expression when the plasticity-controlling *trans* locus carries the Hawaiian allele as when it carries the Bristol allele, and the most extreme differential expression is seen for control by the Hawaiian allele (*p* = 0.002, one-sided *t*-test), exactly the opposite of the predicted pattern. This result may be due to a lack of adaptation of Hawaiian worm strains to their specific environment, possibly due to recent population dispersal.

### Functional Assessment of Temperature-Specific Coregulated Genes

**Figure 4:**
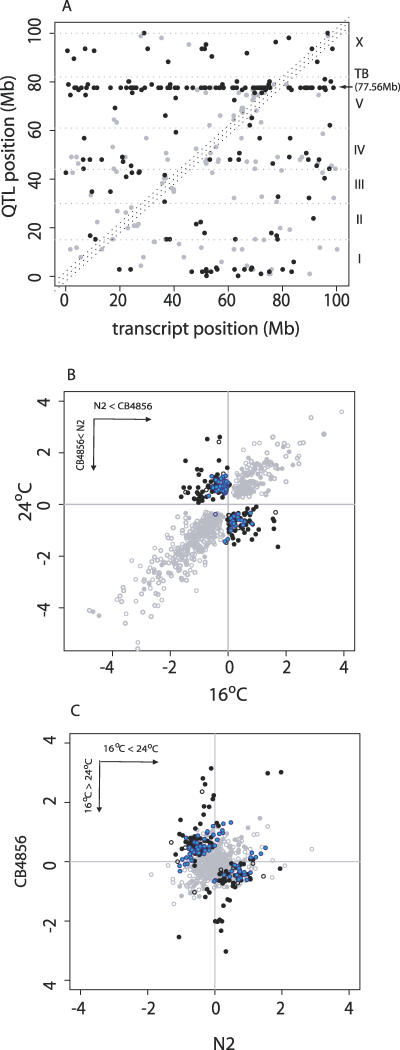
Temperature-Dependent eQTL Effects (A) Comparisons of eQTL and transcript positions for *trans*-regulated genes with significant eQTL and pQTL effects in the full model. The grey dotted horizontal lines separate the genome into different chromosomes. Grey and black circles indicate *trans*-regulated transcripts with significant eQTL effect and with significant pQTL effect, respectively, in the joint analysis. Among the transcripts with significant eQTL effect at both temperatures, a majority (72%) is *cis*-regulated (not included in the plot), while most of the transcripts (85%) with pQTL effect are *trans*-regulated. A horizontal transband was observed at 77.56 Mb (Chromosome V) by joint analysis. The transcripts falling into the region specified by dotted diagonals have *cis*-regulated eQTL (±2 Mb). (B) Comparison of eQTL effect for transcripts at two temperatures. (C) Comparison of temperature-induced differential expression (T effect) for transcripts at two genotypes. In (B) and (C), open and closed circles indicate *cis*-regulated and *trans*-regulated transcripts, respectively; grey and black circles are used for genes with significant eQTL and with significant pQTL effect, respectively. *Trans*-regulated transcripts in the 77.56Mb transband are colored blue.

The most prominent case of pQTL in our dataset is found for a group of 66 genes that map to the same genomic region (Figure 4A) and in 63 out of 66 cases have a strong eQTL only at 24 °C (Figure 4B). Of these genes, 41 have a stronger differential expression for the Hawaii allele (*p* = 0.05, one-sided Wilcoxon test) (Figure 4C). Such a temperature-specific “transband” (TB) seems extremely unlikely, both statistically (*p* << 0.001, hypergeometric test) and biologically, because it has been demonstrated recently that natural selection leads to the elimination of mutations in loci that affect many downstream gene expression levels [13]. To test that the TB is not an artifact, we applied a permutation test (Materials and Methods). The results show that the TB does have a strong and significant genetic component (*p* << 0.0001). In addition to three miRNA genes in this region *(cel-mir-48, cel-mir-241,* and *cel-mir-257),* potential plasticity regulators for the transband genes are listed in Table S4. Additional analysis of the partial correlation coefficients between TB genes (Materials and Methods) shows that they are only partly controlled by the plasticity regulator at the *cis* position. This suggests that these genes are involved in the same pathway and controlled by a number of shared upstream factors. In fact, the TB genes form a conspicuous biological unit according to a gene ontology analysis [22], with enrichments in signal transduction (*p* = 0.03 after multiple testing correction) and cell communication (*p* = 0.04 after multiple testing correction).

The expression patterns of TB genes are also significantly correlated in an independent dataset (Kim dataset) [23] as compared with randomly selected genes (one-way Kolmogorov-Smirnov test, *p* << 0.001) and they are enriched in the “neuronal” functional group (coexpression mount 6, *p* < 7.9 e-14) [23]. It is particularly interesting to see that the group of 66 TB members contains one gene for an FMRFamide-related neuropeptide *(flp-9)* and four for G-protein coupled receptors (C17H11.1, C48C5.1, C24B5.1, and K10C8.2), all of them uncharacterized (Fisher's exact test, *p* = 0.02). Expression variations of neuropeptides of the FMRFamide-related group (*flp-1* [24], *flp-18,* and *flp*-*21* [25]) as well as single amino acid mutations of their G-protein coupled receptor *(npr-1)* [13]underlie important ecological and behavioral differences among C. elegans strains [13,24,25]. It is therefore tempting to speculate that the TB regulator occurred in two different alleles in the pedigree of the two parental populations (N2 and CB4856) because it controls an adaptive phenotypic difference in response to particular thermal conditions.

Interestingly, we found, in our related study of genotype-by-temperature interaction in classical phenotypic traits, that a fertility QTL maps to the immediate vicinity of our transband and shows the same interaction pattern. This suggests that our TB is possibly involved in fertility regulation or regulated by the same upstream factor(s).

### Estimating the Rate of False-Positives in *cis*-QTL Effects

In addition to the *trans*-acting (p)eQTLs, which are the primary focus of the present paper, previous studies[26,27] have also reported numerous *cis*-acting eQTLs, i.e., QTLs that explain expression variation of genes that are physically located at the same position as the QTL. However, as shown in Figure 4B, in our data there is a surprisingly high proportion of *cis*-acting QTLs that show a negative eQTL effect (*p* = 1e-9, Fisher's exact test). One likely explanation is the confounding effect of SNPs on array hybridization. Under the assumption that true *cis*-acting QTLs with positive and negative eQTL effects should occur in equal proportions, we estimate that there are about 226 false positives among the *cis*-acting QTLs (402 total *cis*-QTLs minus twice the number of 88 *cis*-QTLs with positive eQTL effects). Following Hughes et al. [28], we estimate that, on average, a single mismatch or indel in the ten nucleotides most 5′ in our 60-mer probes would result in a significant detectable hybridization difference (about 40% decreased signal). The parental strains, N2 (for which the arrays were designed) and CB4856, differ in their genome sequence by up to one per 873 bp of aligned sequence. Ignoring, for simplicity, the unequal distribution of SNPs in coding and noncoding sequences, we thus estimate the number of genes with one influential SNP to be 238, which corresponds closely to the 226 false positives estimated above. This indicates that *cis* effects are not only less relevant for regulatory gene expression plasticity (topology of the gene regulatory network), but also very prone to hybridization artifacts.

### Power Analysis for Plasticity QTLs

Our ability to detect numerous pQTLs is even more striking when we consider that our approach is likely to underestimate the extent of environment-specific genotype effects (pQTLs). This underestimation is due to the fact that such effects have been diluted by measuring the average abundance of transcripts from all cells of C. elegans (Figure1C); it is hard to detect a large pQTL effect if such an effect is actually cell-type specific.

To check that the number of pQTLs is not seriously underestimated due to our stringent statistical threshold, which might lead to false negatives, we estimated the detection power of interaction for various QTL effect sizes using simulation (Materials and Methods). We detected 98% of interactions if the difference in QTL effect is larger than two at the two temperatures (a pQTL effect of two, Materials and Methods). This suggests that our detection power is more than sufficient.

### Conclusion

Recently the genetic architecture of gene expression has revealed many epistatic interactions in a constant environment [29]. The present results imply that these interactions will change with environmental conditions. In addition, we show that the plasticity of gene expression in C. elegans is mainly controlled by *trans-*acting pQTLs (genotype-by-environment interactions). Our results demonstrate widespread heritable variation in gene expression responses to environmental changes, which are used to generate the first comprehensive map of the genetic polymorphisms underlying differences in expression plasticity.

Future studies of ecological adaptation and evolutionary genetics of gene expression will benefit from this molecular genetics perspective, when exploring the plastic patterns of mRNA levels in different cell types, a wider range of environmental conditions, and a larger number of ecotypes.

## Materials and Methods

### Genetical genomics experiment.

*Strain culturing.* Both N2 and CB parental strains were homozygous. Strains were grown in 9-cm petri dishes at 15 °C or 20 °C on standard nematode growth medium with Escherichia coli strain OP50 as a food source and transferred to new dishes by a chunk of agar once a week. Recombinant inbred lines (RILs) were constructed by putting, on each of ten 6-cm dishes, one J4 hermaphrodite of strain N2 with five males of strain CB4856, and vice versa on each of ten other 6-cm dishes to avoid any maternal or paternal effects. Mating was considered to be successful if the ratio of males to hermaphrodites was approximately 1:1 in the F_1_ hybrids. Approximately 1,500 F_1_ hermaphrodites were transferred to individual dishes in 24-well multiplates and allowed to self-fertilize at 20 °C. This was repeated until F_20_.

*DNA isolation.* For all lines, liquid cultures in S-basal (100 mM NaCl, 50 mM KH_2_PO_4_ [pH 6.0], 5 mg/l cholesterol) were started and allowed to develop for one week in 50-ml tissue-culture flasks at 20 °C. Cultures were transferred to 10-ml blue caps and centrifuged for 5 min at 4,000 rpm. Pelleted nematodes were transferred to a 1.5-ml Eppendorf tube, washed once with 1 ml M9 buffer, and centrifuged for 3 min at 8,000 rpm. After removal of the supernatant, 300 μl lysis buffer (20 mM Tris-HCl [pH 8.0], 2 mM EDTA, 2% Triton X-100) and 5 μl proteinase K (10 mg/ml) were added, and samples were left for 3 h at 65 °C in a rotary shaker. Samples were washed with 400 μl phenol:chloroform:isoamylalcohol (25:24:1) and centrifuged for 3 min at 14,000 rpm, after which the upper layer was transferred to a new tube. This step was repeated once. Next, 30 μl 3 M sodium acetate (pH 5.0) and 750 μl ice-cold isopropanol was added and samples were centrifuged for 3 min at 14,000 rpm. The DNA was washed once with 1 ml 70% ethanol and subsequently dissolved in 100 μl Milli-Q water. 1 μl RNase A was added and samples were incubated for 2–3 h at 37 °C, after which they were stored at 4 °C.

*Genotyping RILs*. All markers were selected on the C. elegans SNP data website, (http://www.genome.wustl.edu/genome/celegans/celegans_snp.cgi). For Chromosomes I, II, III, IV, and X, we selected 20 evenly spaced markers, for Chromosome V we selected 21 markers because this chromosome is larger than the other chromosomes. We selected easily detectable (i.e., with a common restriction enzyme) SNP markers with high *P_snp_*values (*P_snp _*≥ 0.7), of which 75 were already confirmed.

PCR was performed on a Biozym MJ Research PTC-200 Peltier thermal cycler in thin-walled 200-μl reaction tubes under the following conditions: 4 min at 94 °C; 35 cycles of 45 s at 94 °C, 45 s at 56 °C, 45 s at 72 °C; 5 min at 72 °C. Total reaction volume was 10 μl, with 5 μl 20-fold diluted DNA sample, 1 μl 10× PCR buffer (100 mM Tris-HCl [pH 9.0], 15 mM MgCl_2_, 500 mM KCl, 0.1% gelatin, 1% Triton X-100), 0.5 μl 50 mM MgCl_2_, a final primer concentration (Gibco-BRL, www.invitrogen.com; Isogen, www.isogen-lifescience.com; or Proligo, http://www.proligo.com) for each of a 0.4 pmol/μl, a final dNTP (Gibco-BRL) concentration of 0.2 mM, and a final Supertaq polymerase (HT Biotechnology, http://www.sphaero-q.com/HTbiotechnology.html) concentration of 0.02 U/μl.

Subsequently, samples were digested by adding 1μl of restriction enzyme buffer and 3 U of the appropriate restriction enzyme (Boehringer; Invitrogen, http://www.invitrogen.com; New England Biolabs, http://www.neb.com) directly to the sample. BSA was added if necessary. Digestions were performed for 3 h at the appropriate temperature, after which samples were loaded on 1.5%–3% agarose gels (depending on the expected fragment sizes) and run for 1.5 h at 100 V. Suspected mistypings were checked for a second time.

Marker analysis. The order of markers was not based on a constructed linkage map but on their physical position in the sequenced genome. Physical and F2-derived genetic positions were obtained from Wormbase WS106 (http://www.wormbase.org). Marker segregation deviation (segregation distortion) from a 1:1 ratio was analyzed using a χ^2^ test. To correct for Type I errors, we Bonferroni-corrected the significance level of these tests downwards with a factor of 12, which equals the estimated number of independent tests within our dataset: six for the chromosome number multiplied by two for the theoretical number of independent markers on each chromosome (the two outermost ones, which show approximately 50% recombination). Genetic distances between any two neighbouring markers were inferred from recombination fractions using the Kosambi mapping function. Recombination within one chromosome between neighbouring and nonneighbouring markers was analyzed by comparing the observed recombination using a χ^2^ test in which the expected recombination was calculated with the inverse Kosambi function from twice the F2-derived distances between markers to correct for the multiple rounds of meiosis [30].

Association between any two markers on different chromosomes was analyzed for significant deviation from neutrality by comparing the overall number of associations and nonassociations (analogous to (non) recombinants if the markers were close to one another on the same chromosome) for any two markers with a calculated expected number using a χ^2^ test. To obtain a model describing the expected fraction of association based on allele frequency, we performed nonlinear regression on data obtained from a simulation in which we determined the random association between two unlinked loci, each with two alleles, given a specific allele frequency for both alleles at both loci. The random association value finally used as input for the model was an average based on 1,020 replicates in which for each replicate, 80 marker-to-marker comparisons were randomly selected out of a total of 1,000.

*Culturing.* All recombinant inbred lines were reared on NGM agar plates seeded with the OP50 strain of E. coli as a food source. Stock cultures of OP50 were stored at −80 °C, and the bacterial cultures were grown in autoclaved LB medium (10 g peptone, 10 g yeast extract, 5 g NaCl/l water) for 16 h at 37 °C and shaken at 150 rpm. Populations were started with only nonmated hermaphrodites and screened regularly to remove any occurring males.

*Synchronization.* Experiments were carried out with nematodes belonging to the L3 life stage. To determine the entry into this stage at 16 °C and 24 °C, the size of the gonads and vulva were monitored. At 72 h of age, nematodes kept at 16 °C were at the L3 stage, whereas 40 h of age determined this life stage at 24 °C. Populations of each of the RILs were bleached (0.5 M NaOH, 1% hypochlorite) to collect synchronized eggs, which were then inoculated onto fresh dishes. Four replicate dishes of synchronized eggs for each RIL were kept in each of the two temperatures until L3 was reached. The nematodes were then collected and frozen in liquid nitrogen.

*Probe construction and hybridization.* The parental N2 and CB4856 strains differ in their genome sequence by up to one per 873 bp of aligned sequence [19]. Koch et al. reported that 85% of the SNPs were found in noncoding DNA [31]. In an attempt to minimize hybridization differences based on SNPs, 60-mer oligonucleotide microarrays were used in this study. The frozen nematode samples were ground and RNA was extracted using the Trizol method, and cleaned up with the RNeasy Micro kit (Qiagen, http://www1.qiagen.com/). RNA concentration and quality was measured with a NanoDrop spectrophotometer (http://www.nanodrop.com). cDNA was obtained using Array 900 HS kit (Genisphere, http://www.genisphere.com) and Superscript II (Invitrogen). The cDNA samples were hybridized to 60-mer oligo arrays using the Genisphere Array 900 HS protocol. The probes on the arrays cover genes all over the genome. These 60-mers (provided by Washington University) were designed to uniquely represent each gene with proximity to the gene 3′ end and with a minimum of secondary structure potential. All microarray data have been deposited in NCBI's Gene Expression Omnibus (GEO, http://www.ncbi.nlm.nih.gov/geo) and are accessible through the GEO Series accession number listed under the Accession Numbers heading in Supporting Information.

*Pairwise design.* We adopted a novel distant-pair design for the microarray experiments, which was proposed especially for genetic studies on gene expression [20]. In this design, the 80 RILs are hybridized directly on 40 arrays, in pairs that are maximally genetically different.

### Data analysis.

*Full ANOVA model for pQTLs and eQTLs.* The expression data of two temperatures were first analyzed separately by the following ANOVA model [20]

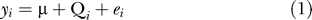
where *y_i_* is the gene's log ratio at the *i*th microarray; μ is the mean; Q*_i_* is −β, 0, β, for arrays comparing *A/B, A/A* or *B*/*B*, and *B*/*A*, respectively; β is the effect of differential allele expression between *A* and *B* at a regulatory locus (or nearby marker) under study; letters *A* and *B* correspond to N2 and CB4856, respectively; and *e_i_* is the residual error (see [20] for details).

Then expression data of two temperatures are combined together and analyzed by a full ANOVA model including T and eQTL*T effects:

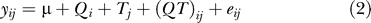
where *y_ij_* is the gene's log ratio at the *i*th microarray (*i* = 1,…*n*) and *j*th temperature, *T_j_* is the temperature effect for *j*th temperature, (*QT*)*_ij_* is the interaction effect (pQTL) between the *i*th eQTL genotype and *j*th temperature, and *e_ij_* is the residual error. To increase the power of detecting interaction, we not only did a genome-wide linkage analysis, but also reduced the multiple testing issue by focusing on those three marker positions that show a maximum eQTL in either the full model or one of the two single temperature models. Interaction was assessed at these three positions using significance thresholds determined by simulation. The same strategy was applied for detecting significant eQTL effects.

*Two-stage search for pQTLs* **.** To increase the power of detecting pQTLs, we not only did a genomewide linkage analysis, but also reduced the multiple testing issue by focusing on those three marker positions that show a maximum eQTL in either the full model or one of the two single temperature models. At the strongest eQTL genome position *SL* (single locus), the corresponding pQTL effect for each transcript was judged to be significant or not. As we expect a pQTL for a gene to occur at the positions with eQTL at one of the two temperatures, we focus on the strongest eQTL genome positions (obtained by separate analysis) for each transcript at 16 °C and 24 °C. These positions we call *TL* (two loci, one locus per temperature). At the *TL*, we checked if the pQTL effect obtained by joint analysis is significant or not. The thresholds were obtained by simulation. A gene is claimed to have a significant interaction effect if it passes the corresponding threshold at one of three positions (*SL* and *TL*). The same strategy was applied for detecting significant eQTL effects.

*Determination of genome-wide significance thresholds.* To calculate the genome-wide threshold for separate analysis, we performed the following five steps. (1) We simulated trait data by randomly sampling from a standard normal distribution (with zero mean and unit variance) 1,000 times under the null hypothesis of no eQTL. We did this for 16 °C and 24 °C. (2) We carried out a single marker analysis for all 1,000 runs mimicking 16 °C and then for the 1,000 run mimicking 24 °C. (3) At each marker, we obtained the corresponding *−*log_10_ *p*. (4) We took the maximum overall markers and stored this value. (5) These values were ordered from low to high over all 1,000 runs, and their 100(1−α) percentile was the estimated critical value (genome-wide threshold).

For the joint analysis the threshold can be obtained in a similar way. After simulating the trait data under the null hypothesis of no eQTL for two temperatures, the joint analysis was applied to the combined data of 16 °C and 24 °C. Then the genome-wide threshold for eQTL and interaction was obtained at a significant *p-*value of 0.001. With the same simulated data, we calculated the −log_10_ *p* of interaction effect at *SL* position or *TL* positions and stored these values, respectively. At the significance level of 0.001, the thresholds for single locus and two-locus analysis can be obtained. The same strategy was applied for the eQTL effect.

In our analysis, we set the genome-wide α to be 0.001 at 16 °C and 24°C, as well as in the joint analysis. This implies that—with 20,490 transcripts—we expect only 0.001 × 20,490 ≈ 20.5 false positives. The threshold of 4.25 was obtained for the separate analyses at both temperatures. For the joint analysis, the genome-wide threshold for eQTL is 4.50 and the single-locus threshold is 4.41. For the interaction effect, the genomewide threshold is 4.56 while the single-locus threshold and two-locus threshold are 2.98 and 3.88, respectively.

*Estimation of temperature-induced differential expression (T effect) based on intensity data.* The intensity-based analysis considers the model

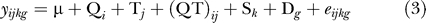
where *y_ij_* is the gene's log intensity at the *i*th microarray (*i* = 1,…,*n*) and *j*th temperature; T*_j_* is the temperature effect for the *j*th temperature; (QT)*_ij_* is the interaction effect (pQTL) between the *i*th eQTL genotype and *j*th temperature, S*_k_* is the random spot effect, D*_g_* is the effect of the *g*th dye, and *e_ijkg_* is the residual error. Firstly, the QTL effects of two temperatures estimated from ratio data using the full model as described in the main text were used to replace the Q*_i_* and (QT)*_ij_* terms by constant values in the intensity-based model. Then temperature-induced differential expression effects were estimated from the remaining model.

*Coexpression of transband genes in Kim dataset [23]*. The experiments in the Kim dataset compare RNA between mutant and wild-type strains or between worms grown under different conditions. The dataset consists of expression of 19,738 genes in 553 experiments. 56 out of 66 of our TB genes are found in the Kim dataset. We calculated all pairwise Pearson correlation coefficients among these 56 genes. Then we randomly chose the same number of genes from the Kim dataset 10,000× and calculated the correlation coefficients of each pair of them. The resulting distribution is compared with that among the original TB genes by a one-way Kolmogorov−Smirnov test (*p-*value ≪ 10^−10^).

*Permutation test for the transband.* We used the real gene expressions of transband genes (i.e., the structure of correlation is kept unchanged), but reassigned different genomes to the different TB randomly to disturb the association between trait and genotype. From 10,000 permutations, the maximum genome-wide number of QTL for each permutation is stored and the 99.9 percentile corresponding to a −log_10_ *p* of 6 was obtained. The results show that the TB does have a strong and significant genetic component (*p* ≪ 0.0001).

*Cis-factor test for transband.* Pearson correlation coefficients (zero order) were first calculated for the trait data of transband genes at 24 °C. Then first order partial correlation coefficients conditioning on the genotype of the transband position (marker 97th) were calculated according to the following formula:

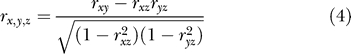
where *r_xy_, r_xz_,* and *r_yz_* are the Pearson correlation coefficient of gene expression between *x* and *y*, *x* and *z*, and *y* and *z*, respectively. We simulated random trait data for the same number of genes as in the TB and calculated corresponding zero and first order correlation coefficients. The results show that the first order partial correlation coefficient on genotype for TB genes decreases significantly from zero order coefficients. However, they are still larger than those for random traits. This indicates that the TB genes are only partly controlled by the master regulator at the QTL position and that these genes are involved in the same pathway and controlled by a number of shared upstream factors.

*Power of detection for pQTL by full model.* Compared with the total number of transcripts, only about 0.8% of 20,000 genes had a detectable pQTL effect, i.e., a surprisingly low proportion of regulatory connections seem to respond differentially to the major environmental change in the two genotypes. To check that this is not due to our stringent threshold, which might lead to false negatives, we estimated the detection power of pQTL for various eQTL effect sizes using simulation. We simulated the expression data for 1,000 genes with an eQTL effect size of B but opposite sign at two temperatures. Then the strategy of searching for pQTL used in real data was applied for the simulated data. The detected proportion of genes with significant pQTL indicates the power of our two-stage search method. With varying B between 0 and 5 with interval 0.25, the power of detection for pQTLs can be estimated. We detect 98% of interactions if the eQTL effect is larger than 1 and has opposite signs at the two temperatures, which corresponds to a pQTL effect of 2. This suggests that our detection power is more than sufficient.

*Master regulator for transband searching.* There are 66 genes with significant pQTL at 77.56 Mb (Chromosome V). It is likely that there is a *cis*-acting master regulator at the QTL position. We first averaged the pQTL profiles for the transband genes and then took a 1.5 dropoff (−log_10_ *p*) to obtain genome region 75.91–79.33 Mb as the searching region. There are 1,180 potential candidates in total with a physical location in this region (819 potential candidates had a measured expression level in our dataset). We divided them into different groups according to their eQTL and pQTL effect and their annotation (see Table S4). The top candidates might be the genes that themselves have a significant pQTL effect (e.g., Y75B12B.3), and eQTL effect, ( e.g., *nhr-54* and *nhr-116*) involved in transcription factor activity, and map in *cis;* i.e., have a possible regulatory polymorphism in their promoter region.

## Supporting Information

Protocol S1 Detailed Description of the RIL Population (115 KB DOC)

Table S1 Information on Cosmid SNP Location, Map Position, and Primers and Restriction Enzymes Used (92 KB PDF)

Table S2 N2 and CB Polymorphisms of the SNP Markers in the RILs and Marker Segregation Ratios (66 KB PDF)

Table S3 Marker-Association Frequencies between Markers on the Same and on Different Chromosomes (173 KB PDF)

Table S4 Potential Master Regulator Candidates for the Transband (17 KB PDF)

### Accession Numbers

The National Center for Biotechnology Information (NCBI) Entrez Gene database (http://www.ncbi.nlm.nih.gov/entrez/query.fcgi?db=gene) accession numbers for the genes discussed in this paper are *C17H11.1* (GeneID181075), *C48C5.1* (GeneID183574), *C24B5.1* (GeneID179301), *flp-1* (GeneID177737), *flp-9* (GeneID178232),*flp-18* (GeneID 180587), *flp-21* (GeneID182944), *K10C8.2* (GeneID187257), *nhr-116* (GeneID180129), *nhr-54* (GeneID180106), *npr-1* (GeneID180752), and *Y75B12B.3* (GeneID190717).

The NCBI Gene Expression Omnibus (GEO, http://www.ncbi.nlm.nih.gov/geo) accesssion number for the microarray data discussed in the paper is GSE5395.

## Abbreviations

eQTL: expression quantitative trait locus
pQTL: plasticity quantitative trait loci
RI: recombinant inbred
RIL: recombinant inbred line
SL: single locus
TB: transband
TL: two loci
