# Supplementary material for: Mapping Determinants of Gene Expression Plasticity by Genetical Genomics in C. elegans
Source: PLoS Genet. 2006 Dec 29;2(12):e222. doi: 10.1371/journal.pgen.0020222 (PMC1756913; doi:10.1371/journal.pgen.0020222)
Supplement: Protocol S1 — (115 KB DOC) [file pgen.0020222.sd001.doc]

**Supporting Protocol**

We present the genotypes of 80 recombinant inbred lines (RILs) that were genome-wide analyzed with 121 Single Nucleotide Polymorphism (SNP) markers. These RILs represent a random subset of a total of approximately 1,000 RILs that were bred in for 20 generations. We describe the patterns of recombination and segregation across chromosomes and the associations between markers on different chromosomes, and try to integrate and explain these patterns with data presently known. The RILs are available upon request. Please contact Jan Kammenga at Jan.Kammenga@wur.nl.

## RESULTS

# SNP marker map: following the segmentation of the chromosomes on the *C. elegans* SNP website (http://genome.wustl.edu/projects/celegans/index.php?snp=1) we selected 20 markers for chromosomes *I, II, III, IV* and *X*, and 21 for chromosome *V* because of its larger physical size. Positions in cM and in bp of the markers used in this study are shown in table S1 for all chromosomes based on Wormbase release WS106 (http://www.wormbase.org) where positions in cM reflect the interpolated genetic positions of the cosmid on which the SNP marker is mapped; the interpolated genetic positions of the SNP marker might therefore differ up to approximately 0.5 cM. The position in % is relative to the full size of the chromosome in bp. Segregation ratios were determined in the RILs with p-values for the χ2-test based on an expected marker ratio of 1:1. Significance levels were Bonferroni corrected with a factor 12 to correct for Type I errors (* <4.2E-3, **<8.3E-4, ***<8.3E-5). Chr stands for chromosome, CB for CB4856. All selected polymorphisms were detectable by digestion with a restriction enzyme. Out of a total of 121 SNPs 74 were previously confirmed by digestion (http://genome.wustl.edu/projects/celegans/index.php?snp=1). The remaining 47 SNPs were up to date only predicted but are in this paper confirmed to be real. The approach of one SNP marker per segment resulted in a well-saturated and well-covered map (table 1 below). The overall average distance between two markers was 835 kbp or 2.38 cM and with an overall average chromosomal coverage of 95.91% if measured in bp or 95.29% if measured in cM. The remaining 5% not covered are the telomeric regions outside the two outermost markers. However, because of the low amount of recombination within these regions it is likely that the genotype outside the outer markers (*i.e.* the parental origin of that region) will be similar to the genotype on the position of the outer marker.

**Strain genotypes:** Singson *et al.* (1999) (S1) and Lewis and Fleming (1995) (S2) reported that hermaphrodites crossed with males prefer male sperm above their own to fertilize eggs. However, this predominance is not absolute (S1) which shows from our data as well. Initially we genotyped 91 RILs but 11 appeared to hold a genomic constitution completely equal to one of the parents. All these cases could be explained by self-fertilization of the parental hermaphrodite, of which 7 out of 43 resulted from a CB4856 hermaphrodite x N2 male cross and 4 out of 47 from a N2 hermaphrodite x CB4856 male cross. The 11 parental-like strains were excluded from subsequent analyses. Table S2 shows the genotypes for all 80 RILs.

**Map expansion due to inbreeding:** compared to the Wormbase F_2_-derived genetic maps (http://www.wormbase.org/, release WS106) our maps show on average an ample twofold expansion (table 1). The sex chromosome is in this sense aberrant with a map expansion of 2.57 instead of around two as observed for the other chromosomes, which might be due to the apparent absence of a central gene cluster (S3). In *C. elegans* a map expansion due to the use of RILs has been observed before (S4). Higher recombination fractions can be explained by the multiple rounds of meioses undergone and is common for RILs bred by self-fertilization or sib-mating (S5). To compare recombinations in a recombinant inbred cross with F_2_-derived data these values should accordingly be divided by two. Such an analysis shows that in most cases (except for three to five comparisons on each chromosome) there is normal recombination between two neighboring markers in our set of strains. As reported before (S3) we observed also a sigmoid relationship between marker distance in cM and marker distance in bp in our cross (figures not shown), with a low amount of recombination in the gene-dense central clusters and higher amounts of recombination on the chromosome arms. Such a relationship between a physical and genetic map is common for other organisms as well (S6).

**Marker segregation ratios:** overall marker frequencies were 52.9% for the N2 allele and 47.1% for the CB4856 allele. Compared with an expected 1:1 ratio with Bonferroni corrected significance levels markers 2 to 17 of chromosome I showed significant segregation distortion (see figure 1). Allele distribution within this region showed a bias towards the N2 allele with a maximum N2:CB4856 allele ratio of 10.29 for marker 4. Chromosomes *II, III, IV, V* and *X* showed slight but non-significant deviations from neutrality. The segregation distortion on chromosome *I* suggests selection for the N2 allele during the development of RILs.

**Associations between unlinked markers on the same chromosome:** recombination fractions between the outermost markers on the same chromosome (which should be inheriting independently) exceeded 50% for chromosome *I* (RF = 53.3%), chromosome *IV* (RF = 55.4%), chromosome *V* (RF = 51.4%) and the sex chromosome *X* (RF = 58.1%), whereas for chromosomes *II* and *III* it was below 50% (44.6% and 41.9% respectively). Recombination within one chromosome exceeding the theoretical maximum of 50% has been described previously (S7, 8) and has been explained as proof of chromatid interference in the four-strand stage. In *C. elegans* meiotic crossover is strictly controlled in a narrow range, with on average exactly one crossover per chromosome per meiosis (S9). However, as the observed values are statistically not different from the expected recombination fractions based on the inverse Kosambi mapping function (RF_exp. chrom. I_ = 47.8% (p=0.33), RF_exp. chrom. II_ = 47.6% (p=0.60), RF_exp. chrom. III_ = 47.9% (p=0.30), RF_exp. chrom. IV_ = 47.0% (p=0.14), RF_exp. chrom. V_ = 47.4% (p=0.49), and RF_exp. chrom. X_ = 47.1% (p=0.06)) they are likely to be the result of random statistical association, possibly due to inbreeding.

**Association between markers on different chromosomes:** Figure 2 shows the expected fraction of association for unlinked markers inheriting independently based on a simulation model. The model can be described by $z=x+y-2xy$ (R^2^ =0.99998) where *z* is the expected fraction of association and *x* and *y* are the marker fractions of one of the two parents on the positions (or loci) (*i.e.* on both positions either the fraction N2 markers or the fraction CB4856 markers). Using this model we analyzed all experimentally obtained pairwise associations between all markers on different chromosomes, which show in some cases strong deviation from the expected association (table S3). Associations significantly exceeding 50% are more abundant: 7.95% of all comparisons between markers on different chromosomes exceed 60% (with a maximum of 71.6%) whereas only 2.13% of all comparisons is smaller than 40% (with a minimum of 32.4%). Although it is likely that some associations will be due to chance some of them could reflect selection for or against combinations of alleles neighboring these markers, and thus indicate the presence of gene interactions or genetic pathways. This would be most likely in case of strong (dis)association between two chromosomal *regions* of markers, for example between chromosome *II* markers 8-20 and chromosome *X* markers 17-20.

**DISCUSSION**

We have genotyped a subset of the inbred progeny genome-wide with 121 markers which together make up a well covered and well saturated genetic map. Analysis revealed that part of the subset was non-recombinant but this could be explained by self-fertilization of the parental hermaphrodite. The remaining RILs showed normal recombination behavior, segregation distortion on a large section of chromosome I and non-random marker associations between (sections of) markers on different chromosomes.

Segregation distortion in intraspecific crosses has been observed before for various organisms (see for example S10, 11, 12), and has been hypothesized and in some cases proven to be caused by selective embryo abortion or gametophyte selection or the existence of segregation distortion regions (SDRs) which map close to or on (previously mapped) specific gametophyte or sterility genes (S10, S13-16). For our *C. elegans* inbred cross there might be a similar association. During the development of RILs we randomly selected one individual per strain for the next generation but experienced sometimes that these individuals did not generate viable offspring. For some lines this happened in several generations during inbreeding, for others only once. In most cases we succeeded in rescuing the particular inbred line by selecting another individual from the specific strain (as described in *Methods*) but in 66 out of 1245 (5.3%) we failed to do so. Therefore, even though we tried to avoid selection for particular nematodes or traits in each generation by randomly selecting individuals, the only trait which we selected for (because we had to) is the ability to reproduce.

Non-random associations between markers on different chromosomes might suggest selection for or against specific combinations of alleles. However, it can result from a slight preferential inheritance of alleles during the breeding of the RILs, as discussed by Williams *et al.* (2001) (S12) who also observed statistical associations between intervals on different chromosomes in recombinant inbred crosses with mice. To test this hypothesis we simulated independent inheritance of two loci each with two alleles (A and B). First we calculated the expected frequency of both alleles at both loci after 20 generations of inbreeding given a specific probability of transfer to the next generation for each allele. We performed simulations with either equal or unequal probabilities for similar alleles on the different loci (*i.e.* both A or both B). After randomization of both allele distributions we selected 80 out of 1000 comparisons, similar to the approach in practice, and analyzed this subsample for recombination (*i.e.* statistical associations due to chance). We repeated this procedure 1020 times for each combination of probabilities. The results are shown in table S3, and show that in a large number of comparisons statistical associations are indeed not unlikely to occur, even when the probability of inheritance is 0.5 for both alleles. Within the probability range of 0.45-0.55 there is no statistical difference between the results of the different simulations. In addition, the simulations in which the probability of inheritance for similar alleles was unequal shows higher maximum association values. Taking into account the slight bias towards alleles from one of the two strains on the different chromosomes this suggests that the associations in our dataset probably do not result from allelic interactions. However, in the simulations the number of associations with values above and below 50% were present in an equal amount. In our “real” dataset we observed more non-random associations with an association value above 50% than below 50% of which some tend to form clusters of non-random association with a clear center (see for example the comparison between chromosomes *II* and *X*). This might indicate interactions between alleles or otherwise some kind of hybrid vigor due to which “heterozygotes” are more likely to survive.

Recombination between (the two outermost) markers on the same chromosome exceeds 50% for chromosome *I* (RF_max_ = 54.7%), chromosome *IV* (RF_max_= 55.4%), chromosome *V* (RF_max_= 55.4%) and the sex chromosome *X* (RF_max_= 60.8%), whereas for chromosomes II and III recombination stays below 50% (45.9% and 47.3% respectively). Recombination within one chromosome exceeding the theoretical maximum of 50% has been described previously (S7, 8) and has been explained as proof of chromatid interference in the four-strand stage. In this study, however, it might be the result of non-random statistical associations as well as the observed frequencies are well within the range of possible frequencies of two independently inherited alleles.

**References**

S1 Singson, A., Hill, K.L., and L'Hernault, S.W. 1999. Sperm competition in the absence of fertilization in Caenorhabditis elegans. Genetics 152: 201-208.

S2 Lewis, J.A. and Fleming, J.T. 1995. Basic culture methods. In Caenorhabditis elegans: Modern Biological Analysis of an Organism (eds. Epstein, H.F. and Shakes, D.C.), pp. 3-29. Academic Press, Inc., San Diego, California.

S3 Barnes, T.M., Kohara, Y., Coulson, A., and Hekimi, S. 1995. Meiotic Recombination, Noncoding DNA and Genomic Organization in Caenorhabditis elegans. Genetics 141: 159-179.

S4 Ayyadevara, S., Ayyadevara, R., Hou, S., Thaden, J.J., and Reis, R.J.S. 2001. Genetic mapping of quantitative trait loci governing longevity of Caenorhabditis elegans in recombinant-inbred progeny of a Bergerac-BO x RC301 interstrain cross. Genetics 157: 655-666.

S5 Dixon, L.K. 1993. Use of Recombinant Inbred Strains to Map Genes of Aging. Genetica 91: 151-165.

S6 Nachman, M.W. 2002. Variation in recombination rate across the genome: evidence and implications. Curr. Opin. Genet. Dev. 12: 657-663.

S7 Fisher, R.A. and Mather, K. 1936. Verification in Mice of the Possibility of more than Fifty per cent Recombination. Nature 137: 362-363.

S8 Wright, M.E. 1947. Two Sex-linkages in the House Mouse, with Unusual Recombination Values. Heredity 1: 349-354.

S9 Hillers, K.J., and Villeneuve A.M., 2003, Choromosome-wide control of meiotic crossing over in *C. elegans,* Current Biology, 13:1641-1647

S10 Lu, H., Romero-Severson, J., and Bernardo, R. 2002. Chromosomal regions associated with segregation distortion in maize. Theor. Appl. Genet. 105: 622-628.

S11 Paran, I., Goldman, I., Tanksley, S.D., and Zamir, D. 1995. Recombinant inbred lines for genetic mapping in tomato. Theor. Appl. Genet. 90: 542-548.

S12 Williams, R.W., Gu, J., Qi, S., and Lu, L. 2001. The genetic structure of recombinant inbred mice: high-resolution consensus maps for complex traits analysis. Genome Biology 2: research0046.0041-0046.0018.

S13 Grant, V. 1975. Altered Segregation. Pp. 228-250 in Genetics of flowering plants.

S14 Xu, Y., L. Zhu, J. Xiao, N. Huang, and S.R. McCouch. 1997. Chromosomal regions associated with segregation distortion of molecular markers in F-2, backcross, doubled haploid, and recombinant inbred populations in rice (Oryza sativa L). Mol. Gen. Genet. 253: 535-545.

S15 Vogl, C. and S.Z. Xu. 2000. Multipoint mapping of viability and segregation distorting loci using molecular markers. Genetics 155: 1439-1447.

S16 Korbecka, G., P.G.L. Klinkhamer, and K. Vrieling. 2002. Selective embryo abortion hypothesis revisited - A molecular approach. Plant Biol. 4: 298-310.

# Table 1. Genetic map size and marker distance of the RI cross

|  |  |  |  | marker distance*^b^* | | | | | | |  |  |  |  |  | coverage *^d^* | |
| --- | --- | --- | --- | --- | --- | --- | --- | --- | --- | --- | --- | --- | --- | --- | --- | --- | --- |
|  | size*^a^* | |  | cM | | |  | kbp | | |  | genetic map size*^c^* | | |  | (%) | |
| Chr | cM | Mbp |  | avg | max | min |  | avg | max | min |  | this study | WB | expansion |  | in cM | in bp |
| I | 48.21 | 15.08 |  | 2.48 | 6.34 | 0.25 |  | 778 | 1230 | 304 |  | 86.33 | 47.18 | 1.83 |  | 98.07 | 97.86 |
| II | 55.62 | 15.28 |  | 2.45 | 7.05 | 0.09 |  | 767 | 1093 | 414 |  | 91.15 | 46.48 | 1.96 |  | 95.43 | 83.56 |
| III | 48.98 | 13.78 |  | 2.52 | 7.85 | 0.14 |  | 677 | 1053 | 320 |  | 88.95 | 47.93 | 1.86 |  | 93.30 | 97.85 |
| IV | 44.79 | 17.49 |  | 2.29 | 11.44 | 0.16 |  | 891 | 1230 | 612 |  | 94.76 | 43.52 | 2.18 |  | 96.79 | 97.17 |
| V | 45.66 | 20.92 |  | 2.26 | 5.33 | 0.31 |  | 1008 | 1369 | 544 |  | 86.88 | 45.24 | 1.92 |  | 96.39 | 99.08 |
| X | 45.42 | 17.71 |  | 2.30 | 6.30 | 0.47 |  | 890 | 1297 | 639 |  | 112.22 | 43.72 | 2.57 |  | 95.50 | 96.25 |
|  |  |  |  |  |  |  |  |  |  |  |  |  |  |  |  |  |  |
| Overall | 288.69 | 100.27 |  | 2.38 | - | - |  | 835 | - | - |  | 560.30 | 274.07 | 2.04 |  | 95.91 | 95.29 |

*^a^* chromosome size in cM and Mbp are based on Wormbase 106

*^b^* maximum and minimum marker distances in cM are not necessarily correlated with maximum and minimum marker distances in kbp

*^c^* values for the genetic map size do not reflect the full chromosomal size but the distance between the two outermost (distal) markers; expansion is the distance between those markers according to this study divided by their distance according to Wormbase 106 (WB)

*^d^* coverage is the fraction of the chromosome located between the two outermost markers if measured in cM or bp

# FIGURE LEGENDS

Figure 1. N2:CB4856 marker segregation ratios for all markers on the six chromosomes. Segregation distortion is present on a large section of chromosome I. Exact segregation ratios are shown in the table S2.

Figure 2. Model to calculate the expected association frequencies between two loci given different marker frequencies at each locus. The model is based on simulation data and can be described by *z* = *x* + *y* – 2*xy* (R^2^=0.99998), in which *z* is the expected association frequency and *x* and *y* are the marker frequencies of the marker inherited from the same parent at both positions (or loci) *X* and *Y*.

FIGURE 1

N2:CB4856 ratio

# FIGURE 2

Expected level of association
