# Supplementary material for: Mapping Determinants of Gene Expression Plasticity by Genetical Genomics in C. elegans
Source: PLoS Genet. 2006 Dec 29;2(12):e222. doi: 10.1371/journal.pgen.0020222 (PMC1756913; doi:10.1371/journal.pgen.0020222)
Supplement: Table S1 — (92 KB PDF) [file pgen.0020222.st001.pdf]

chromosome	marker#	Cosmid name	SNP location on cosmid	Map position		Position in % of chromosomal size		Primers				Polymorphism		Previously identified	Restriction enzyme	Digestion results (bp)	
				bp	cM	if size in bp	if size in cM	Forward	Tm	Reverse	Tm	Wormbase 106 name	type			N2	CB4856
I	1	F56C11	22274	168807	-18.2603	1.12	1.17	atcgaaatccccttgtaccc	60	aggtcgctccaaactttctagg	66	pkP1050	T[T/G]TAAA	Y	*Dra* I	95, 475	570
	2	C54G6	1258	992188	-17.2825	6.58	3.20	cgcgtttcgtaatgtatcg	56	gaacttccaggtcactctgg	62	pkP1101	GAATT[C/A]	Y	*Eco* RI	104, 278	382
	3	Y71G12A	10851	1884415	-11.9590	12.47	14.24	acctagaataagaatgggcagg	64	tttggcttattgccgatatgc	60	pkP1103	GT[A/G]C	Y	*Rsa* I	85, 231	316
	4	W03D8	34384	2818973	-6.1004	18.69	26.39	cgaacttttatccgtcaccg	60	caccccaattaatctgtgcg	60	pkP1052	TT[T/-]AAA	Y	*Dra* I	174, 334	507
	5	Y47G6A	69880	3502476	-3.5488	23.23	31.68	ttgtttcacggcaaacctag	58	aatgaggagcaggaagtttg	58	snp_Y47G6A.8	TTT[T/A]AA	N	*Dra* I	98, 200, 252	25, 98, 200, 227
	6	T03A1	28270	4338254	-1.4887	28.77	35.96	actagccaatgtcaagcctc	60	cagaaatcagttgatcagcg	58	snp_T03A1.3[?]	GTC[A/G]AC	N	*Sal* I	525	252, 273
	7	F57C9	23691	4845515	-0.6162	32.13	37.77	atgtgtctgcaatgtggtgg	60	aaatctcgcggtctcgaaac	60	pkP1054	CCCGG[G/A]	Y	*Sma* I	263, 294	557
	8	C34G6	15466	5893622	0.4597	39.08	40.00	attgacagggtctgaagatg	58	gacatccgactcttcattcg	60	snp_C34G6.3	T[T/C]GA	N	*Taq* I	127, 258	42, 127, 216
	9	K04F10	19618	6359867	0.9366	42.17	40.99	atcattctccaggccacgttac	66	ctgaactagtcgaacaaacccc	66	pkP1057	CAT[T/A]TG	Y	*Nde* I	595	294, 301
	10	D2030	16772	7589863	2.1576	50.33	43.52	ggtgtactggctccttccaatg	68	cgcactcactttgattacaacg	64	pkP1116	AAC[G/A]TT	Y	*Acl* I	113, 224	337
	11	F55D12	7673	7894081	2.4087	52.35	44.04	cattactatgctgccagtcc	60	tgcatgggtatcgtactagc	60	snp_F55D12.3	GGTAC[C/T]	N	*Kpn* I	213, 329	542
	12	VF39H2L	3079	8654360	2.9456	57.39	45.16	ttcaggctccactttatgcc	60	catctgggacgttctttcac	60	pkP1059	TTTA[G/A]A	Y	*Dra* I	641	219, 422
	13	C36F7	23997	9569914	3.7959	63.46	46.92	tatcgaaaggtttcacacctgg	64	gccgaaatataagctgtacaag	62	pkP1122	[T/A]TGCAT	Y	*Nsi* I	388	154, 234
	14	ZC247	14561	10259909	4.7801	68.03	48.96	ccatgcttccgtttcaaacg	60	taccagctgtcaaaagtagg	58	snp_ZC247.2	GGATC[C/T]	N	*Bam* HI	233, 238	471
	15	W02B9	8155	11085295	6.0193	73.51	51.53	tgatttccgtagaatttcgcg	60	cggataacgatcaggtagag	60	snp_W02B9.2	[G/T]GCC	N	*Hae* III	84, 158, 250	158, 334
	16	ZK1151	1902	11760182	7.5226	77.98	54.65	gcacctcgtctttgaatctg	60	agaacttcttgcgtcagctc	60	pkP1068	[A/G]GCC	Y	*Hae* III	682	212, 470
	17	T07D10	11635	12614280	13.8659	83.65	67.81	cttggtgtggggagaatatagg	66	tttgtccggattgactctgc	60	pkP1070	GA[C/T]C	Y	*Mbo* I or *Sau* 3AI	62, 305	62, 96, 209
	18	M01E5	454	13280531	17.4648	88.06	75.27	ccgagtgttgaagtgagaac	60	ttgtgaagcccgttgagttg	60	snp_M01E5.1	GTCTA[C/T]	N	*Acc* I	129, 383	512
	19	C37A5	17439	14162948	22.9129	93.92	86.57	ctcatgcatgatttcgaggg	60	aaatccaacaggagcaggac	60	pkP1071	GAATT[C/T]	Y	*Eco* RI	241, 286	527
	20	ZK909	10916	14958660	28.9192	99.19	99.03	cacaagtggtttggaagtaccg	66	caacaaagggatagatcacggg	66	pkP1072	AA[C/G]CTT	Y	*Hind* III	451	215, 236
II	1	T01D1	14052	176721	-17.3780	1.16	1.36	aagaggtgttcttctgcagc	60	accatacacgcagttcattc	58	pkP2101	TT[T/-]AAA	Y	*Dra* I	217, 402	619
	2	Y57G7A	4213	1270097	-15.4084	8.31	4.90	tttgcagatttccgatactgtg	62	ttttgtcgtaagaccttttggtg	62	pkP2135	TTT[A/G]AA	Y	*Dra* I	143, 299	442
	3	F58E1	13123	1683953	-14.9140	11.02	5.79	ttcaaccgtcttgactgctac	62	atggtaaccgcatgctttg	56	pkp2136	[C/T]CTAGA	Y	*Xba* I	336	58, 278
	4	F29A7	8269	2755074	-10.0773	18.03	14.48	gcgatttgacctctgtaagg	60	tccatctccagttgtcgttc	60	pkP2114	A[C/G]CT	Y	*Alu* I	647	274, 373
	5	F18A12	7765	3403575	-5.9476	22.28	21.91	aggagtttgccgtactactg	60	taaaaggcattctccggtgg	60	snp_F18A12.2	[A/G]GCT	N	*Alu* I	69, 114, 319	183, 319
	6	F56D3	5769	4147051	-5.4083	27.14	22.88	ggaaccattgatggagttcg	60	atactaccctcacctcgtag	60	snp_F56D3.3	GG[T/A]ACC	N	*Kpn* I	104, 145	249
	7	C33F10	5689	4800868	-3.5209	31.42	26.27	ctgttgatccaaatgctgcg	60	aaatggtctcgagtagatgg	58	snp_C33F10.1	CAT[T/A]TG	N	*Nde* I	576	274, 302
	8	F10C1	18148	5761743	-0.9662	37.71	30.86	tgtccagacgacaccaattag	62	caaatgcgagaactgaattg	56	pkP2147	AGTAC[C/T]	Y	*Sca* I	406	122, 284
	9	K03H9	16942	6427387	-0.3160	42.07	32.03	atcgtggagaacctggtaagtg	66	cgtccactcatcaaagatgg	60	pkP2106	CAT[G/T]	Y	*Nla* III	252, 638	890
	10	F32A5	35775	7257517	0.4409	47.50	33.39	attactggaaccacatctgg	58	attcttgctggagacgactg	60	snp_F32A5.2	[G/C]TCAG	N	*Dde* I	380	180, 200
	11	T05C12	27580	8195651	0.7867	53.64	34.01	gttgtcccaatcctcatcattg	64	tgaatttcgagaatggagtacg	62	pkP2151	G[T/A]TC	Y	*Mbo* I or *Sau* 3AI	381	99, 282
	12	K08F8	10717	8748358	0.8759	57.26	34.17	gacacagatgtcctcaagtg	60	gtatgtcaagcttctccctg	60	snp_K08F8.2	C[T/C]CAG	N	*Dde* I	49, 53, 62, 240, 260	49, 62, 240, 313
	13	Y51B9A	7381	9401070	1.2287	61.53	34.81	ctaggagatgctccaacttctg	66	tcgcatcaagtccatctctg	60	pkP2109	GAATT[C/T]	Y	*Eco* RI	327, 329	656
	14	E04D5	3557	10414073	2.4889	68.16	37.07	tagcaaacaaagtgttctcgg	60	gtgattccgtgcacataccg	62	snp_E04D5.1[?]	GTGCA[C/T]	Y	*Apa* LI	13, 155, 262	13, 417
	15	F37H8	7719	11180836	3.2851	73.18	38.50	ttctcacaacttcttttccaag	60	ttcactatttccctcgctgg	60	pkP2110	TC[A/G]A	Y	*Taq* I	16, 112, 573	16, 112, 194, 379
	16	F44E5	3808	11752128	4.0335	76.92	39.85	tcatcatcaatctcagggcg	60	attccgtgtgagacatctgg	60	snp_F44E5.2	TTA[A/G]	N	*Mse* I	216, 249	465
	17	Y38E10A	60924	12630696	10.8343	82.67	52.08	aggaataaagcggtggaacg	60	atcctttgaatcgcccatcg	60	snp_Y38E10A.4[?]	T[G/T]AATTAA	N	*Pac* I	741	342, 399
	18	F15D4	22813	13235559	15.8706	86.62	61.13	ttcccattttcctcccag	54	tcaaaaacccagacactgg	56	pkP2116	TTT[T/A]AA	Y	*Dra* I	517	119, 398
	19	Y39G8B	11219	13980541	22.0573	91.50	72.25	aaacgtttcgagtgtgatcg	58	atatcgcagtttgcccagtg	60	snp_Y39G8B.1	T[C/T]TAAA	N	see comment	439	~539
	20	Y54E2A	20317	14758293	29.1025	96.59	84.92	aatgaagttcgcgcttccag	60	tttctcgagagttgtgtgtc	58	snp_Y54E2.1	[A/G]TCGAT	N	*Cla* I	179, 277	456
III	1	K10F12	2024	360894	-27.0345	2.62	0.74	atcacttgctccatgagagg	60	aaaatcaccgatagcctgcg	60	snp_K10F12.5	G[G/A]ATTC	N	*Eco* RI	642	305, 337
	2	K02F3	17776	840469	-25.8751	6.10	3.10	ggcataccatagtatgcggtac	66	gactcttgggattcttgggaac	66	pkP3045	TT[T/A]A	Y	*Mse* I	23, 31, 110, 170	23, 31, 50, 60, 170
	3	B0524	10276	1893290	-18.0248	13.74	19.13	ataaagtgaccaagcgctgc	60	aattactggattcgcggctg	60	snp_B0524.4	CTG[T/A]G	N	*Dde* I	400	152, 248
	4	E02H9	23593	2459092	-13.6165	17.84	28.13	gaaactgacagcattgcctac	62	aaagtgcgctccagccactag	66	snp_E02H9.8	T[C/T]AA	N	*Mse* I	366	130, 236
	5	H06I04	15629	3068098	-9.3329	22.26	36.88	tatccaagtggcttttgtgg	60	aagaccatgcttgactgacc	60	pkp3057	[C/G]TTAG	Y	*Dde* I	235, 270	505
	6	ZK1058	25918	3923438	-4.3164	28.47	47.12	ccaactgtcgacgatagagatg	66	aattccgtgtagcgaatcg	56	pkP3096	GAAT[T/G]C	Y	*Eco* RI	208, 258	466
	7	F25F2	15611	4517529	-3.2032	32.78	49.39	tgttctagacatatcgggag	58	ttggagatgacacttgagac	58	snp_F25F2.1	CTGA[A/G]	N	*Dde* I	488	189, 299
	8	W03A5	26462	5431252	-1.7369	39.40	52.38	cagacatttaggagtaggcagg	66	gagtacgcgggtcatttttg	60	pkP3047	[A/T]TAA	Y	*Mse* I	365	164, 201
	9	C28H8	43335	5925983	-1.4086	42.99	53.05	aatgagttactgcgctctac	58	tttcttgtttcagcgcgatg	58	snp_C28H8.2	GTA[T/C]	N	*Rsa* I	37, 160	37, 58, 102
	10	R13F6	12315	6847169	-0.9176	49.68	54.06	catctgaaactcacgggaagtc	66	agttacaacctcgggtcgtg	62	pkP3100	[G/A]TAC	Y	*Rsa* I	244, 277	521
	11	F56C9	13335	7320107	-0.7451	53.11	54.41	aagtagagtacacatggcag	58	tcactgtgcagtcttacctc	60	snp_F56C9.1	TT/A/T]AAA	N	*Dra* I	275	125, 150
	12	C06G4	21304	7998164	-0.4552	58.03	55.00	tttctcttccactcttgctc	58	ttacttagaagcacgcagtc	58	snp_C06G4.4	AAG[C/T]TT	N	*Hin* dIII	130, 205, 245	130, 450
	13	F10E9	39795	8318553	-0.3121	60.35	55.29	agcagatgaaagttccgacg	60	ccccgctgtggttattatac	60	pkP3049	GTAT[A/T]C	Y	*Acc* I	254, 600	854
	14	C40H1	186	9308858	0.3746	67.54	56.69	aattcagcgaacacagtagc	58	aaaagttgccgatgaatgcg	58	pkP3107	[C/T]TTAAA	Y	*Dra* I	62, 108	54, 54, 62
	15	M04D8	10485	10027496	1.4094	72.75	58.81	ttcagagcaccccttttctgg	60	aatccgttggcattgcaaag	58	snp_M04D8.2	TTT[A/T]AA	N	*Dra* I	58, 78, 217, 297	78, 217, 355
	16	Y39A1A	17456	10613119	4.3513	77.00	64.81	ccccacagcttttaatcttg	58	aaagcctttcggaacttcc	56	pkP3059	TCT[A/G]GA	Y	*Xba* I	31, 211, 342	31, 553
	17	T28D6	5473	11341120	8.6849	82.28	73.66	tttcgtgtacgaacgtctcc	60	catttctcccactcttgctg	60	pkP3060	TTTAA[G/A]	Y	*Dra* I	501	218, 283
	18	Y75B8A	233018	12301725	14.9161	89.25	86.38	aaacagcattgtcgacgagc	60	agcctaagcccaagctttag	60	pkP3075	A[G/A]GCTT	Y	*Hin* dIII	16, 315	16, 56, 259
	19	Y111B2A	247164	12739834	18.4884	92.43	93.67	aatcagatatattccaggcgg	62	ctaccaaccctgacaacacg	62	pkP3113	C[A/C]TG	Y	*Nla* III	32, 47, 195	32, 242
	20	Y39E4B	153208	13220073	20.8962	95.91	98.59	ttaccgctttgagagggatg	60	tttccgacaataggcaattcc	60	snp_Y39E4B.10	ATG[C/T]AT	N	*Nsi* I	221, 301	522
IV	1	Y38C1AA	4951	151889	-27.2932	0.87	0.67	aggagtaagggtaacgtcag	60	acttgcatttaccgccagag	60	snp_Y38C1A.2	T[T/C]TAGA	N	*Xba* I	515	74, 441
	2	C07B3	1388	1381409	-19.9736	7.90	17.01	agtcaccccacacaagttatg	62	aagtgagtaagaaagaggcgg	62	snp_C07B3.2	CA[G/A]TTT	Y	*Apo* I	79, 133	64, 69, 79
	3	C37F5	15538	2288742	-8.5343	13.08	42.55	cttttcgtggaaattcgcac	58	attctagctatgagcttctg	56	snp_C37F5.13	GAGT[T/C]	N	*Hin* fI	478	152, 326
	4	Y67D8C	30669	3067374	-4.7814	17.53	50.93	tcatagtgtccgcttttctg	58	ttcgagcaatttcaggac	52	snp_Y67D8C.4	TTTAA[A/-]	N	*Dra* I	44, 103, 120, 237	103, 163, 237
	5	F56D6	30166	3920366	-1.7108	22.41	57.78	atcgcgaacgagataaagag	58	tgttgactctgactacgaag	58	snp_F56D6.2	[T/C]TCGAG	N	*Xho* I	645	254, 391
	6	E03H12	15628	4991858	1.4532	28.54	64.85	ccaaacaacctacagaaaatgc	62	aagatattcatgcgtcgtagtg	62	pkP4071	TT[A/T]AAA	Y	*Dra* I	56, 71, 457	56, 71, 172, 285
	7	C10G6	5412	5819735	2.4204	33.27	67.01	gacttgcttacatctgtagc	58	gaatacacgcaaaagtgcag	58	snp_C10G6.1	T[C/T]AA	N	*Mse* I	21, 212	21, 101, 111
	8	F38A5	19715	6599685	3.2114	37.73	68.77	gcaccatcttctgctccaac	62	ttaaccttggagtgactgcg	62	pkP4075	TCTA[A/G]A	Y	*Xba* I	547	243, 304
	9	C48A7	11713	7405743	3.3697	42.33	69.13	aagaacgagaacgcactgtg	60	tgttggttgcgcgaaaactc	60	snp_C48A7.2	TTT[T/A]AA	N	*Dra* I	588	292, 296
	10	D2096	39721	8397264	3.7498	48.00	69.98	acgaaaaatcacagagcggg	60	aatcaacaacggacgacgag	60	pkP4039	GA[A/T]TTC	Y	*Eco* RI	202, 650	852
	11	C01F6	7442	9102404	4.0579	52.03	70.66	tcagcgagaaaatagacatcg	60	aacctgtacttgttcaccgc	64	pkP4080	ATGCA[A/T]	Y	*Nsi* I	343	135, 208
	12	K08F4	2338	10122930	4.5536	57.87	71.77	agttgagttgttgcatggtgac	64	gaaaattgtcgcaacaactgg	60	dbP3	TTTAA[G/A]	Y	*Dra* I	33, 451	33, 207, 244
	13	ZK596	10024	10909560	4.7525	62.36	72.21	cgatgcaatttgattgacc	54	aggttacctgttgggtattgg	62	dbP6	GC[A/G]C	Y	*Hha* I	427	155, 272
	14	ZK792	2021	11668242	5.1651	66.70	73.14	aatggttgtattgggccagg	60	tcttggcttgaacttgacgg	60	snp_ZK792.2[?]	[T/C]CCGGG	Y	*Sma* I	728	54, 674
	15	C36H8	8331	12748880	6.2380	72.88	75.53	ggtctgatgccttctttgtg	60	ccaaaataccctgtacctgc	60	pkP4047	G[A/G]TC	Y	*Mbo* I or *Sau* 3AI	45, 131, 194, 195	45, 195, 325
	16	Y45F10C	14189	13667267	10.2031	78.13	84.38	cccaaaatggagtctctgtg	60	cccaagctttcataatcgcg	60	pkP4048	TTT[A/G]AA	Y	*Dra* I	63, 80, 246	143, 246
	17	Y57G11B	16234	14566567	12.1345	83.27	88.70	gtaaataccccacatttcaagc	62	ttccaattgttcaaagcctg	56	pkP4095	TTT[A/G]AA	Y	*Dra* I	48, 162, 283	48, 445
	18	Y40H7A	37766	15178091	13.0337	86.76	90.70	ttacaccaacttggagcttg	58	gtgtttcgagcaaacggaac	60	snp_Y40H7.7	GCG[T/C]	N	*Hha* I	489	119, 370
	19	C35D6	39314	16371991	15.0474	93.59	95.20	cttcagtaacagttgttgtgg	60	gttcacatatacttgtcgagg	60	snp_C35D6.4	TT[C/T]AAA	N	*Dra* I	99, 149, 163	68, 95, 99, 149
	20	Y116A8C	184204	17084259	16.2268	97.66	97.83	ttcaccgtctcatatgcatc	58	aaacgcaatgccgcactgaa	60	snp_Y116A8C.6	CAT[A/G]	N	*Nla* III	479	205, 274
V	1	ZK488	1867	590471	-20.0903	2.82	0.18	aaagagaagcactactgcac	58	ttgactagtggatgacagag	58	snp_ZK488.2	GA[A/T]C	N	*Mbo* I or *Sau* 3AI	299	146, 153
	2	F36H9	7973	1773465	-17.4243	8.48	6.01	cggaaaattgcgactgtc	54	attaggactgcttggcttcc	60	pkP5076	TT[T/A]AAA	Y	*Dra* I	163, 331	494
	3	F37B4	19808	2878209	-12.0977	13.76	17.68	aagttttcgattggcgacag	58	tagagtagtgctgaccgag	58	snp_F37B4.2	TT[T/A]AAA	N	*Dra* I	33, 64, 125, 157, 272	97, 125, 157, 272
	4	T22F3	27378	3606323	-8.8643	17.24	24.76	tctaccgaaaatatcccgtg	58	ttcagcaagttcacatccgtc	62	snp_T22F3.3	GG[C/T]C	N	*Hae* III	88, 304	392
	5	Y61A9LA	14897	4550758	-4.9530	21.75	33.33	tagagaaatggacacccgac	60	atcgagttgaaaacacgcaag	60	snp_Y61A9L.1	TT[A/T]AAA	N	*Dra* I	152, 531	152, 231, 300
	6	ZC250	19324	5814403	-0.6700	27.79	42.71	tatttgcacgaatggcctga	58	atccgcttacataaggcttg	58	snp_ZC250.1	TT[C/T]AAA	N	*Dra* I	399	83, 316
	7	F44C4	3079	6612376	0.1310	31.60	44.46	cgggaacctgactcttctattg	66	tctcgagaaaattgctgatgc	60	pkP5113	GTCGA[A/C]	Y	*Sal* I	343	99, 244
	8	C12D5	22518	7690531	0.8527	36.76	46.04	aacatcgaagttcacgtcag	58	attggagcaaggagacaaac	58	snp_C12D5.4	[A/T]CTAGA	N	*Xba* I	255	88, 167
	9	F25G6	25139	8567608	1.4605	40.95	47.38	aatttcgtcaccctctttgaac	62	gccgatatctacttgtgtaccg	66	snp_F25G6.2 [?]	TTTA[A/T]A	Y	*Dra* I	93, 223	316
	10	F36D4	17110	9405441	2.0011	44.95	48.56	attccgcagctttgatacag	58	tttcagccaatactcggtcg	60	snp_F36D4.1	[G/A]GTACT	N	*Sca* I	332	81, 251
	11	AC3	3025	10368660	2.5149	49.56	49.68	ctcttagataccctttctgcgc	66	ttcagcgtttggtctgacgtag	66	pkP5064	T[T/C]TAAA	Y	*Dra* I	105, 386	491
	12	F32D8	39286	10912994	2.8247	52.16	50.36	gtcgcacctttgctcaatc	60	tcgaaaattgccctccctac	60	pkP5065	TC[G/A]	Y	*Taq* I	33, 77, 252	33, 329
	13	D2023	2486	11796050	3.3745	56.38	51.57	ccgccagctattccaatttc	60	acaacgaagaagagccacag	60	pkP5067	GAA[T/C]C	Y	*Hin* fI	25, 122, 568	25, 690
	14	W07G4	11352	13033974	4.8313	62.30	54.76	atcttcgtccaacaggtttc	58	aagagcaactccagcgatag	60	snp_W07G4.1	[T/C]CGG	N	*Msp* I	441	92, 349
	15	R10D12	16645	13951861	5.7659	66.68	56.80	aggatttttgagcaacctagc	60	tcgacttttgaagtacactgcc	64	pkP5125	TTT[T/A]AA	Y	*Dra* I	56, 482	56, 183, 299
	16	T09F5	10378	15158759	7.4648	72.45	60.53	gcacacaaatgataccacgc	60	cttaaagcgctgcagaaagc	60	pkP5071	[C/G]AATTC	Y	*Eco* RI	67, 79, 601	67, 79, 238, 363
	17	C06C6	17146	16008404	9.2990	76.51	64.54	aaatctctacaggccgacag	60	ttcttgtcgccaggcgaatg	62	snp_C06C6.2/3	TTT[A/G]AA	N	*Dra* I	99, 300	125, 274
	18	Y20C6A	2502	17377158	12.3353	83.06	71.19	gcaatcgagaagctccaaag	60	ttgaaaaagcccagtagccc	60	snp_Y20C6.1	CT[A/G]CAG	Y	*Pst* I	853	338, 515
	19	Y51A2D	92865	18574593	16.3810	88.78	80.05	caggcatattacatgggatagg	64	ccatctcacctccattctgtg	64	pkP5082	TT[A/T]AAA	Y	*Dra* I	394	121, 273
	20	Y43F8B	71256	19525561	21.6572	93.32	91.61	tagtgatgcactacggtttc	58	tatcatagttgaccccgtca	58	snp_Y43F8B.2	CTA[G/A]G	N	*Dde* I	359	109, 250
	21	F38A6	14347	20758352	25.1471	99.22	99.25	tttatccgcagggacttgac	60	tctcctctccctcatggttaac	66	pkP5075	TTTTA[A/C]A	Y	*Dra* I	128, 183, 672	CB-:128, 855
X	1	F02G3	5645	696607	-19.3558	3.93	3.17	ctgaaatcgtgtgcgaagtc	60	tcttcagttggttaaggtcctc	64	pkP6140	GGAT[C/T]C	Y	*Bam* HI	290, 292	582
	2	D1005	9623	1462371	-17.8928	8.26	6.39	tttttccaggcgtccatgtag	62	actcctgatccttcaaaaccg	62	pkP6142	AGA[T/C]CT	Y	*Bgl* II	56, 301	357
	3	F47G3	13697	2389438	-15.1501	13.49	12.43	gccgaactatataacagccg	60	cataccaacacagttgcacg	60	pkP6118	[T/A]CGA	Y	*Taq* I	47, 73, 85, 127	47, 73, 212
	4	F11D5	27263	3328811	-10.5284	18.79	22.60	tcgtggcaccataaaagtg	56	gattcagatcaaacagaggtgg	64	pkP6120	TT[C/T]AAA	Y	*Dra* I	244	117, 127
	5	ZK470	27170	4161495	-8.1624	23.49	27.81	ccaaaacggccaagtatcag	60	ttgcactctttcctcttccg	60	pkP6105	TTT[T/A]AA	Y	*Dra* I	40, 72, 422	40, 72, 96, 326
	6	ZC449	4683	5010049	-6.1517	28.28	32.24	attgtgacgaatcggaaccc	60	agtagactgagaccgcacaaac	66	pkP6107	TTTAA[T/A]	Y	*Dra* I	146	63, 83
	7	C54H2	23555	5770179	-4.2898	32.57	36.33	ttaaaagctggctctagtgttg	62	agcaattatagtgtcattgccg	62	pkP6151	TT[T/C]AAA	Y	*Dra* I	57, 60, 96, 130	57, 96, 190
	8	B0403	33022	7067019	-1.9388	39.89	41.51	aacctccaacttcgaaggac	60	caagactacgatggaggacg	62	pkP6155	AGA[C/T]CT	Y	*Bgl* II	364	87, 277
	9	F45E1	9628	7982354	-0.9370	45.06	43.72	tttcttgacacctccggtag	60	ctcactctggtctttttccg	60	snp_F45E10.1 [?]	GA[A/G]TTC	Y	*Eco* RI	260, 516	776
	10	F53A9	5088	8691677	0.4135	49.07	46.69	aagacacccatccatgcatatc	64	caaattgtcagccgttgtttc	60	pkP6158	AAG[C/T]TT	Y	*Hin* dIII	179, 185	364
	11	T20B5	4201	9330879	1.1075	52.67	48.22	ttatcacctcaagccgaaga	58	tactctaacagtcccaatgc	58	snp_T20B5.1	C[A/G]TG	N	*Nla* III	136, 205	341
	12	R07E3	10700	10323428	1.8242	58.28	49.80	accatatcaagtggtgtcgg	60	acttggtgagcattccgcac	62	pkP6128	[C/A]ATG	Y	*Nla* III	271, 378	649
	13	C05C9	8686	11142277	3.0298	62.90	52.45	cgcactgacttttttccagc	60	acactagaatctccacacctcc	66	pkP6114	TTT[T/A]AA	Y	*Dra* I	66, 541	66, 95, 446
	14	C23H4	9217	12208257	6.8455	68.92	60.85	ccttcaatcgcttttgtagg	58	gatttacgtaacggctgagg	60	pkP6125	TCG[G/A]	Y	*Taq* I	37, 90, 691	37, 90, 260, 431
	15	K02A4	17281	12891891	8.9333	72.78	65.45	cgaggagacaccagtttaaacg	66	agaaggacgcgaactgaaatg	62	pkP6131	TGA[A/T]CA	Y	*Bcl* I	468	216, 252
	16	T21H8	29883	13893785	14.4125	78.43	77.51	attggagcactcttacggc	58	atccgattgaagttcaccc	56	pkP6163	C[G/A]TG	Y	*Nla* III	78, 245	64, 78, 181
	17	F54E4	29832	14600585	16.6104	82.42	82.35	agaattcgaatcgaggcaag	58	atcgcttctctaacctcacc	60	snp_F54E4.2	GTA[C/A]	N	*Rsa* I	111, 286	397
	18	K09E9	8901	15620481	22.9098	88.18	96.22	ttatcttccagcacccgatg	60	aaaacgaggagcagcatgac	60	snp_K09E9.1	[G/C]TTAAC	N	*Hpa* I	77, 188	265
	19	W09B12	8861	16366136	23.8963	92.39	98.39	cctcaggatttaccagtgacac	66	ttagtcttgcgcccctagag	62	pkP6169	AGAT[G/C]T	Y	*Bgl* II	330	116, 214
	20	C16H3	20454	17614442	24.3623	99.44	99.41	cgatgcggtttcctagcttac	64	attgcccatttcaagccc	54	pkP6171	ATC[G/A]AT	Y	*Cla* I	101, 270	371

Column title		Additional information (if applicable)
chromosome		
marker#		One marker per segment (as defined by the SNP website: http://genome.wustl.edu/projects/celegans/index.php?snp=1) was selected
Cosmid name		
SNP location on cosmid		
Map position	bp	Position in bp is position of SNP on cosmid (relative to start of cosmid) + starting position of cosmid relative to chromosome
	cM	Interpolated position of cosmid on which SNP is mapped in cM. Obtained from Wormbase release 106 (http://www.wormbase.org)
Position in % of chromosomal size	if size in bp	Both relative to total size of chromosome in bp or cM according to Wormbase release 106
	if size in cM	
Primers	Forward	
	Tm	Primer melting temperature calculated by 2°Cx(#A+#T)+4°Cx(#C+#G)
	Reverse	
	Tm	Primer melting temperature calculated by 2°Cx(#A+#T)+4°Cx(#C+#G)
Polymorphism	name	names by which SNPs can be found in Wormbase. SNPs with a [?] are the likely/possible identities of the SNPs according to the authors; these could not be recovered in Wormbase.
	type	Shows the kind of polymorphism between brackets in the following order: [N2/CB4856].
Restriction enzyme		Restriction enzyme which has been used for detection. In some cases other restriction enzymes can be used as well, see http://rebase.neb.com/rebase/rebase.html for more details on restriction enzymes
Digestion results (bp)	N2	expected pattern for the N2-allele after digestion.
	CB4856	expected pattern for the CB4856-allele after digestion.
