# Supplementary material for: Mapping Determinants of Gene Expression Plasticity by Genetical Genomics in C. elegans
Source: PLoS Genet. 2006 Dec 29;2(12):e222. doi: 10.1371/journal.pgen.0020222 (PMC1756913; doi:10.1371/journal.pgen.0020222)
Supplement: Table S2 — (66 KB PDF) [file pgen.0020222.st002.pdf]

| chromosome | marker# | WN1 | WN2 | WN3 | WN4 | WN5 | WN6 | WN7 | WN8 | WN9 | WN10 | WN11 | WN12 | WN13 | WN14 | WN15 | WN16 | WN17 | WN18 | WN19 | WN20 | WN21 | WN22 | WN23 | WN24 | WN25 |
|---|---|---|---|---|---|---|---|---|---|---|---|---|---|---|---|---|---|---|---|---|---|---|---|---|---|---|
| | | RILs: a = inheritance of N2-polymorphism (red color), b = inheritance of CB4856-polymorphism (green color); white is missing data | | | | | | | | | | | | | | | | | | | | | | | | |
| I | 1 | a | a | a | a | a | b | b | a | a | b | a | a | b | a | a | a | a | b | a | b | a | b | b | a | a |
| | 2 | a | a | a | a | a | b | b | a | a | b | a | a | b | a | a | a | a | b | a | b | a | a | b | a | a |
| | 3 | a | a | a | a | a | a | a | a | a | a | a | a | b | a | a | a | a | b | a | a | a | a | ? | a | a |
| | 4 | a | a | a | a | b | a | a | a | a | a | a | a | a | a | a | a | a | a | a | b | a | a | ? | a | a |
| | 5 | a | a | a | a | b | a | b | a | a | a | a | b | a | a | a | a | a | a | a | b | b | a | ? | a | a |
| | 6 | a | a | a | a | a | a | b | a | a | a | a | b | a | a | a | a | a | b | a | b | b | b | b | a | b |
| | 7 | a | a | a | a | a | a | b | a | a | a | a | b | a | a | a | a | a | b | a | b | b | b | a | a | b |
| | 8 | a | a | a | a | a | a | b | a | a | a | a | b | a | a | a | a | a | b | a | b | b | b | ? | a | b |
| | 9 | a | a | a | a | a | a | b | a | a | a | a | b | a | a | a | a | a | b | a | b | b | b | a | a | b |
| | 10 | a | a | a | a | a | a | b | a | a | a | a | b | a | a | a | a | a | b | a | b | b | b | ? | a | b |
| | 11 | a | a | a | a | a | a | b | a | a | a | a | a | a | a | a | a | a | b | a | b | b | b | a | a | b |
| | 12 | a | a | a | a | a | a | b | a | a | a | a | a | a | a | a | a | a | b | a | b | b | b | a | a | b |
| | 13 | a | a | a | a | a | a | b | a | a | a | a | b | a | a | a | a | a | b | a | b | b | b | a | a | b |
| | 14 | a | a | a | a | a | a | b | a | a | a | a | b | a | a | a | a | a | b | a | b | b | b | a | a | b |
| | 15 | a | a | a | a | a | a | b | a | a | a | a | b | a | a | a | a | a | b | a | b | b | b | ? | a | b |
| | 16 | a | a | a | a | a | a | b | a | a | a | a | b | a | a | a | a | a | b | a | b | b | b | ? | a | b |
| | 17 | a | a | a | a | a | a | b | a | a | a | a | b | a | a | a | a | b | b | a | b | b | b | a | a | b |
| | 18 | a | a | a | b | a | a | b | a | a | a | a | b | a | a | b | a | b | b | a | b | b | b | a | a | b |
| | 19 | a | a | a | b | a | a | b | a | a | a | a | b | a | a | b | a | b | b | a | b | b | b | a | a | b |
| | 20 | a | a | a | b | a | a | b | a | a | a | a | b | a | a | b | a | b | a | a | b | b | b | a | a | b |
| II | 1 | b | a | b | b | b | a | b | b | a | b | b | a | b | b | a | b | a | a | b | a | b | b | ? | b | a |
| | 2 | b | a | b | b | b | a | b | b | a | b | b | a | b | b | a | b | a | a | b | a | b | b | ? | b | a |
| | 3 | b | a | b | b | b | a | b | a | a | b | b | a | b | b | a | b | a | a | b | a | b | b | ? | b | a |
| | 4 | b | a | b | a | b | a | b | a | a | b | b | a | b | b | a | b | a | b | b | a | a | b | b | b | a |
| | 5 | b | a | b | a | b | a | b | a | a | b | b | a | b | b | a | b | a | b | a | a | a | b | b | b | a |
| | 6 | b | a | b | a | b | a | b | b | a | b | b | a | b | b | a | b | a | b | a | a | a | b | ? | b | a |
| | 7 | b | a | b | a | a | a | b | b | a | b | b | a | b | b | a | b | a | b | a | a | a | b | ? | b | a |
| | 8 | b | a | b | a | b | a | b | b | a | b | b | a | b | b | a | b | a | b | a | b | a | b | ? | b | a |
| | 9 | b | a | b | a | b | b | b | b | a | b | b | a | b | b | a | b | a | b | a | b | a | b | ? | b | a |
| | 10 | b | a | b | a | b | b | b | b | a | b | b | a | b | a | a | b | a | b | a | b | a | b | ? | b | a |
| | 11 | b | a | b | a | b | b | b | b | a | b | b | a | b | a | a | b | a | b | a | b | a | b | ? | b | a |
| | 12 | b | a | b | a | b | b | b | b | a | b | b | a | b | a | a | b | a | b | a | b | a | b | ? | b | a |
| | 13 | b | a | b | a | b | b | b | b | a | b | b | a | b | a | a | b | a | b | a | b | a | b | ? | b | a |
| | 14 | b | a | b | a | b | b | b | b | a | b | b | a | b | a | a | b | a | b | a | b | a | b | ? | b | a |
| | 15 | b | a | b | a | b | b | b | b | a | b | b | a | b | b | a | b | a | b | a | b | a | b | ? | b | a |
| | 16 | a | a | b | a | b | b | b | b | a | b | b | a | b | b | a | b | a | b | a | b | a | b | ? | b | a |
| | 17 | a | a | a | a | b | b | a | b | a | b | b | a | a | b | a | b | a | b | a | b | a | b | ? | b | a |
| | 18 | a | a | a | a | b | b | a | b | a | b | b | a | a | b | a | b | a | b | a | b | a | a | ? | b | a |
| | 19 | a | a | a | a | a | b | a | b | a | b | b | a | a | b | b | a | a | a | a | a | a | a | b | a | a |
| | 20 | a | a | a | a | a | b | a | b | a | b | b | a | a | b | b | a | a | a | a | a | a | a | ? | a | a |
| III | 1 | b | a | b | b | a | b | b | b | b | a | b | b | a | a | a | a | b | b | b | b | b | a | ? | b | b |
| | 2 | b | a | b | b | a | b | b | b | b | a | b | b | a | a | a | a | b | b | b | b | b | a | ? | b | b |
| | 3 | b | a | a | b | a | b | b | b | b | a | b | b | a | a | a | a | b | b | b | b | b | a | ? | b | b |
| | 4 | b | a | b | b | a | b | a | b | b | a | b | b | a | a | a | a | b | b | b | a | b | a | ? | b | b |
| | 5 | b | a | a | b | a | a | b | b | a | a | b | a | a | a | a | a | b | b | a | a | b | a | ? | b | b |
| | 6 | b | a | a | b | a | a | b | b | a | a | a | a | a | a | a | a | b | b | a | a | b | a | ? | b | b |
| | 7 | b | a | a | b | a | a | b | b | a | a | a | a | b | a | a | a | b | b | a | a | b | a | ? | b | b |
| | 8 | b | a | a | b | a | a | b | b | a | a | a | a | b | a | a | a | b | b | a | a | b | b | ? | b | b |
| | 9 | b | a | a | b | a | a | b | b | a | a | a | a | b | a | a | a | b | b | a | a | b | a | ? | b | b |
| | 10 | b | a | a | b | a | a | b | b | a | a | a | a | b | a | a | a | b | b | a | a | b | b | ? | b | b |
| | 11 | b | a | a | b | a | a | b | b | a | a | a | a | b | a | a | a | b | b | a | a | b | b | ? | b | b |
| | 12 | b | a | a | b | a | a | b | b | a | a | a | a | b | a | a | a | b | a | a | a | b | b | ? | b | b |
| | 13 | b | a | a | b | a | a | b | b | a | a | a | a | b | a | a | a | b | b | a | a | b | b | ? | b | b |
| | 14 | b | a | a | b | a | a | b | b | a | a | a | a | b | a | a | a | b | b | a | a | b | b | ? | b | b |
| | 15 | b | a | a | b | b | a | b | b | a | a | a | a | b | a | a | a | b | b | a | a | b | a | ? | b | b |
| | 16 | b | a | a | b | b | a | b | b | a | a | a | a | b | a | a | a | b | b | a | a | b | a | ? | b | b |
| | 17 | a | a | a | b | b | a | b | a | a | a | a | a | a | a | a | a | b | a | a | a | b | a | ? | b | a |
| | 18 | a | a | b | b | b | a | b | a | b | a | a | a | a | b | a | a | b | a | a | a | b | a | ? | b | a |
| | 19 | a | b | b | b | b | a | b | a | b | a | a | a | a | b | a | a | b | b | a | a | b | a | ? | b | a |
| | 20 | a | b | b | b | b | a | b | a | a | a | a | a | a | b | a | a | b | b | a | a | b | a | ? | b | a |
| IV | 1 | a | b | b | b | b | a | b | b | b | a | a | b | a | a | a | a | a | b | b | a | b | a | ? | a | a |
| | 2 | a | b | b | b | b | a | b | b | b | b | a | b | a | a | a | a | a | b | b | a | b | a | ? | b | b |
| | 3 | b | b | b | b | b | a | b | b | b | a | a | a | a | a | a | a | a | b | b | a | b | a | ? | b | b |
| | 4 | b | b | b | b | b | a | b | b | b | a | a | b | a | a | a | a | a | b | b | a | b | a | ? | a | b |
| | 5 | b | b | b | b | b | b | b | b | b | a | a | a | a | a | a | a | a | b | a | a | b | a | ? | a | b |
| | 6 | b | b | b | b | b | b | b | b | b | a | a | a | a | a | a | a | a | b | a | a | b | a | ? | a | b |
| | 7 | b | b | b | b | b | b | b | b | b | a | a | a | a | a | a | a | a | b | a | a | b | a | ? | a | b |
| | 8 | b | b | b | b | b | b | b | b | b | a | a | a | a | a | a | a | a | b | a | a | b | a | ? | a | b |
| | 9 | b | b | b | b | b | b | b | b | b | a | a | a | a | a | a | a | a | b | a | a | b | a | ? | a | b |
| | 10 | b | b | b | b | b | b | b | b | b | a | a | a | a | b | a | a | a | b | a | a | b | a | ? | a | b |
| | 11 | b | b | b | b | b | b | b | b | b | a | a | a | a | b | a | a | a | b | a | a | b | a | ? | a | b |
| | 12 | b | b | b | b | b | b | b | b | b | a | a | a | a | b | a | a | a | b | a | a | b | a | ? | a | b |
| | 13 | b | b | b | b | b | b | b | b | b | a | a | a | a | b | a | a | a | b | a | a | b | a | ? | a | b |
| | 14 | b | b | b | b | b | b | b | b | b | a | a | a | a | b | a | a | a | b | a | a | b | a | ? | a | b |
| | 15 | b | b | b | b | b | b | b | b | b | a | b | a | b | b | a | b | a | b | a | a | b | a | ? | a | b |
| | 16 | b | b | b | b | a | b | b | a | b | b | b | a | b | a | a | b | b | b | a | a | b | b | ? | a | b |
| | 17 | b | b | a | b | a | b | b | a | b | b | b | a | b | a | a | b | b | b | a | a | b | b | ? | a | b |
| | 18 | b | b | a | b | a | b | a | a | b | b | b | a | b | a | a | b | b | b | a | a | b | b | ? | a | b |
| | 19 | b | b | a | b | a | b | a | a | b | b | b | a | b | a | a | b | a | b | a | a | a | b | ? | a | b |
| | 20 | b | b | a | b | a | b | a | a | b | b | b | a | b | a | a | b | a | b | a | a | a | b | ? | a | b |
| V | 1 | b | b | a | b | b | b | a | b | b | b | a | a | b | a | b | a | b | a | a | b | a | a | ? | b | b |
| | 2 | b | b | a | b | b | b | a | b | b | b | a | a | b | b | b | a | b | a | a | b | a | a | ? | b | b |
| | 3 | b | b | a | b | b | b | b | b | b | a | a | a | b | b | b | a | b | a | a | b | a | b | ? | b | a |
| | 4 | b | b | a | b | b | b | b | b | b | a | a | a | b | b | b | a | b | a | a | b | a | b | ? | b | a |
| | 5 | b | b | a | b | b | b | b | b | b | a | a | b | b | b | b | a | b | a | a | b | a | b | ? | b | b |
| | 6 | b | b | a | b | b | b | b | b | b | a | a | b | b | b | b | b | a | a | a | b | a | b | ? | a | b |
| | 7 | b | b | a | b | b | b | b | b | b | a | a | b | b | b | b | b | a | a | a | b | a | b | ? | a | b |
| | 8 | b | b | a | b | b | b | b | b | b | a | a | b | b | b | b | b | a | a | a | b | a | b | ? | a | b |
| | 9 | b | b | a | b | b | b | b | b | b | a | a | b | b | b | b | b | a | a | a | b | a | b | ? | a | b |
| | 10 | b | b | a | b | b | b | b | b | b | a | a | b | b | b | b | b | a | a | a | b | a | b | ? | a | b |
| | 11 | b | b | a | b | b | b | b | b | a | a | a | b | b | a | b | b | a | a | a | b | a | b | ? | a | b |
| | 12 | b | b | a | b | b | b | b | b | a | a | a | b | b | a | b | b | a | a | a | b | a | b | ? | a | b |
| | 13 | b | b | a | b | b | b | b | b | a | a | a | b | b | a | b | b | a | a | a | b | a | b | ? | a | b |
| | 14 | a | b | a | b | b | b | b | b | a | a | a | b | b | a | b | b | a | a | a | b | a | b | ? | a | b |
| | 15 | a | b | a | b | b | b | b | b | a | a | a | b | b | a | b | b | a | a | a | b | a | b | ? | a | b |
| | 16 | a | a | a | b | b | b | b | b | a | a | a | b | b | a | b | b | a | a | a | b | a | b | ? | a | a |
| | 17 | a | a | a | b | b | b | b | b | a | a | a | b | b | a | b | b | a | b | a | b | b | b | ? | a | a |
| | 18 | a | b | a | b | b | b | b | b | a | a | b | b | b | a | b | b | a | b | a | b | b | b | ? | a | a |
| | 19 | a | b | a | b | b | b | b | b | a | a | b | b | b | a | b | b | a | b | a | b | b | b | ? | a | a |
| | 20 | a | b | a | a | b | b | b | b | a | a | b | b | b | a | b | b | a | b | a | b | b | b | ? | a | a |
| | 21 | a | b | a | a | b | b | b | b | a | a | b | b | b | a | b | b | a | b | a | b | b | b | ? | a | a |
| X | 1 | a | a | a | a | b | a | a | a | a | b | a | b | a | b | b | a | a | a | b | a | a | b | ? | b | a |
| | 2 | a | a | a | a | b | a | a | a | a | b | a | b | a | b | b | a | a | a | b | a | a | b | ? | b | a |
| | 3 | a | a | a | a | b | a | a | a | b | b | a | b | b | b | b | a | a | a | b | a | a | b | ? | b | a |
| | 4 | a | a | a | a | a | a | a | a | b | b | a | b | b | b | b | a | a | a | b | a | a | b | ? | b | a |
| | 5 | a | a | a | a | a | a | a | a | b | b | a | b | b | a | b | a | a | a | b | a | a | b | ? | b | b |
| | 6 | a | a | a | a | a | a | a | a | b | b | a | b | b | a | b | a | a | a | b | a | a | b | ? | b | b |
| | 7 | a | a | a | a | a | a | a | a | b | b | a | b | b | a | b | a | a | a | b | a | a | b | ? | a | b |
| | 8 | b | a | a | a | a | a | a | a | b | b | a | b | b | b | b | a | a | b | b | a | a | b | ? | a | b |
| | 9 | b | a | a | a | a | a | a | a | b | b | a | b | b | a | b | a | a | b | b | a | a | b | ? | a | b |
| | 10 | b | a | a | b | a | a | a | a | b | b | a | b | b | a | b | a | a | b | b | a | a | b | ? | b | b |
| | 11 | b | a | a | b | a | a | a | a | b | b | a | b | b | a | b | a | a | b | a | a | a | b | ? | b | b |
| | 12 | b | a | a | b | a | a | a | a | b | b | a | b | b | a | b | a | a | a | a | a | a | b | ? | b | b |
| | 13 | b | b | a | b | a | a | a | a | b | b | a | b | b | a | b | a | a | a | a | a | a | b | ? | b | b |
| | 14 | b | b | a | b | a | a | a | a | b | b | a | b | b | a | b | a | a | a | a | b | a | b | ? | b | b |
| | 15 | b | b | a | b | a | a | a | a | b | b | a | b | b | a | b | a | a | a | a | a | a | b | ? | b | b |
| | 16 | b | b | a | b | a | a | a | a | b | b | a | b | b | a | b | b | b | b | a | a | a | b | ? | b | b |
| | 17 | b | b | a | b | a | a | a | b | b | b | a | b | b | a | b | a | b | b | a | a | a | b | ? | b | b |
| | 18 | b | b | b | b | a | b | a | b | b | a | a | b | b | a | b | a | b | b | a | a | a | b | ? | b | b |
| | 19 | b | b | b | b | a | b | a | b | b | a | a | b | b | a | b | a | b | b | a | a | a | b | ? | b | b |
| | 20 | b | b | b | b | a | b | a | b | b | a | a | b | b | a | b | a | b | b | a | a | a | b | ? | b | b |
| Number of N2-markers | | 46 | 72 | 80 | 45 | 57 | 63 | 32 | 48 | 67 | 74 | 86 | 63 | 47 | 83 | 75 | 80 | 84 | 38 | 99 | 66 | 59 | 38 | - | 59 | 42 |
| Number of CB4856-markers | | 75 | 49 | 41 | 76 | 64 | 58 | 89 | 73 | 54 | 47 | 35 | 58 | 74 | 38 | 46 | 41 | 37 | 83 | 22 | 55 | 62 | 83 | - | 62 | 79 |
| RIL-specific N2/CB4856 ratio | | 0.613 | 1.469 | 1.951 | 0.592 | 0.891 | 1.086 | 0.36 | 0.658 | 1.241 | 1.574 | 2.457 | 1.086 | 0.635 | 2.184 | 1.63 | 1.951 | 2.27 | 0.458 | 4.5 | 1.2 | 0.952 | 0.458 | - | 0.952 | 0.532 |

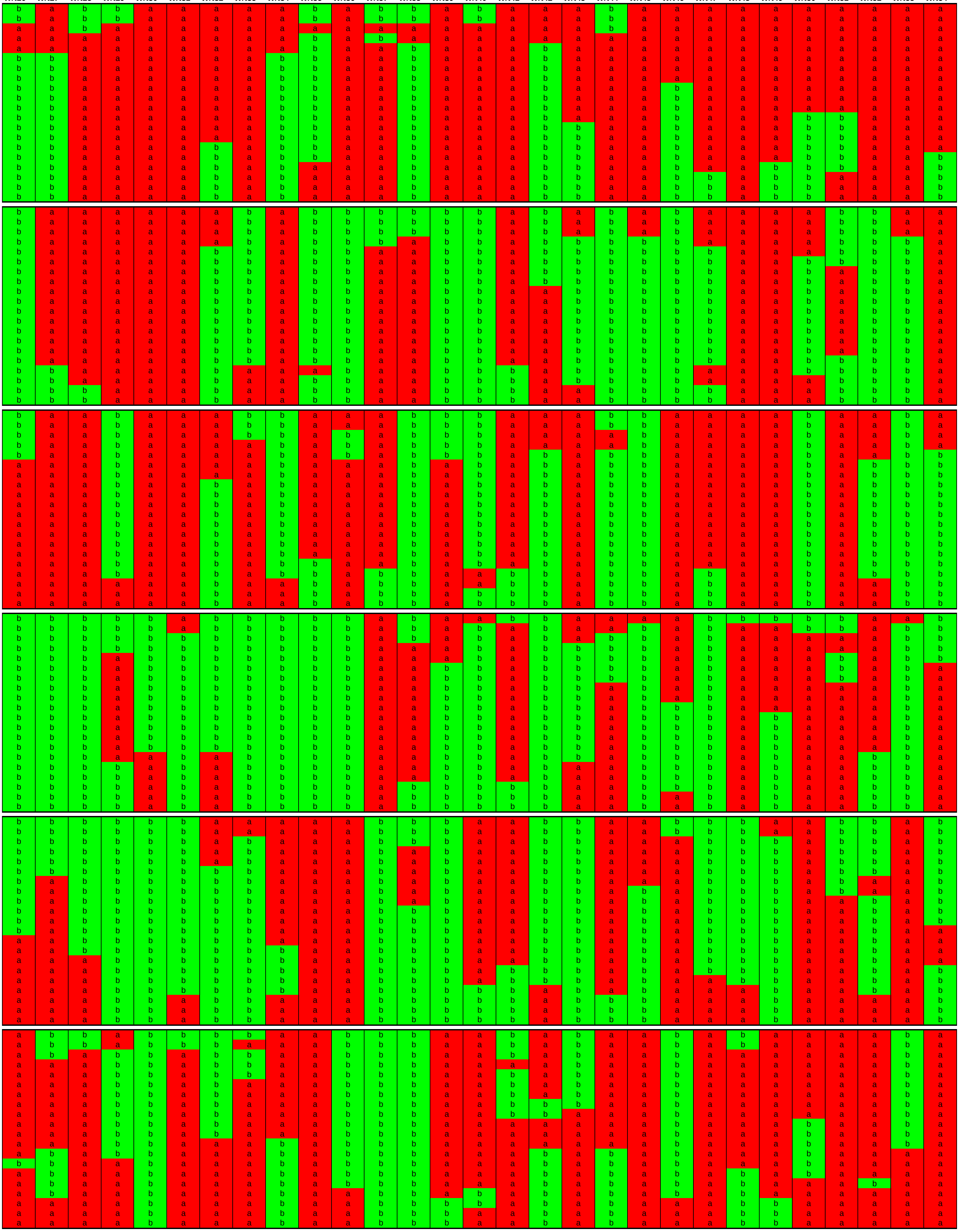

| WN26 | WN27 | WN28 | WN29 | WN30 | WN31 | WN32 | WN33 | WN34 | WN35 | WN36 | WN37 | WN38 | WN39 | WN40 | WN41 | WN42 | WN43 | WN44 | WN45 | WN46 | WN47 | WN48 | WN49 | WN50 | WN51 | WN52 | WN53 | WN54 |
|---|---|---|---|---|---|---|---|---|---|---|---|---|---|---|---|---|---|---|---|---|---|---|---|---|---|---|---|---|
| 46 | 68 | 80 | 61 | 66 | 83 | 45 | 59 | 55 | 62 | 62 | 69 | 39 | 57 | 56 | 95 | 34 | 57 | 64 | 51 | 61 | 64 | 95 | 84 | 72 | 91 | 66 | 53 | 78 |
| 75 | 53 | 41 | 60 | 55 | 38 | 76 | 62 | 66 | 59 | 59 | 52 | 82 | 64 | 65 | 26 | 87 | 64 | 57 | 70 | 60 | 57 | 26 | 37 | 49 | 30 | 55 | 68 | 43 |
| 0.613 | 1.283 | 1.951 | 1.017 | 1.2 | 2.184 | 0.592 | 0.952 | 0.833 | 1.051 | 1.051 | 1.327 | 0.476 | 0.891 | 0.862 | 3.654 | 0.391 | 0.891 | 1.123 | 0.729 | 1.017 | 1.123 | 3.654 | 2.27 | 1.469 | 3.033 | 1.2 | 0.779 | 1.814 |

| WN55 | WN56 | WN57 | WN58 | WN59 | WN60 | WN61 | WN62 | WN63 | WN64 | WN65 | WN66 | WN67 | WN68 | WN69 | WN70 | WN71 | WN72 | WN73 | WN74 | WN75 | WN76 | WN77 | WN78 | WN79 | WN80 | Number of N2-markers | Number of CB4856-markers |
|---|---|---|---|---|---|---|---|---|---|---|---|---|---|---|---|---|---|---|---|---|---|---|---|---|---|---|---|
| a | a | b | b | b | a | a | a | b | a | a | b | b | a | a | b | a | b | b | a | a | a | a | b | b | a | 53 | 27 |
| a | a | b | b | b | a | a | a | b | a | a | b | b | a | a | b | a | b | b | a | a | a | a | b | b | a | 54 | 26 |
| a | a | b | a | b | a | a | a | a | a | a | a | b | a | a | a | a | a | a | a | a | a | a | b | a | a | 71 | 8 |
| b | a | b | a | a | a | a | a | a | a | a | a | a | a | a | a | b | a | a | a | a | a | a | a | a | a | 72 | 7 |
| b | a | b | a | a | a | a | a | a | b | a | a | a | b | a | a | b | a | a | a | a | a | a | a | a | a | 66 | 13 |
| b | a | b | a | a | a | a | a | a | b | b | a | a | b | a | a | b | a | a | a | a | a | a | a | a | a | 60 | 20 |
| b | a | b | a | a | a | a | a | a | b | b | a | a | b | a | a | b | a | a | a | a | a | a | a | a | a | 61 | 19 |
| a | a | b | a | a | a | a | a | a | b | b | a | a | b | a | a | b | a | a | a | a | a | a | a | a | a | 61 | 18 |
| a | a | b | a | a | a | a | a | a | b | b | a | a | b | a | a | b | a | a | a | a | a | a | a | a | a | 61 | 19 |
| a | a | b | a | a | a | a | a | a | b | b | a | a | b | a | a | b | a | a | a | a | a | a | a | a | a | 60 | 19 |
| a | a | b | a | a | a | a | a | a | b | b | a | a | b | a | a | b | a | b | a | a | a | a | a | a | a | 61 | 19 |
| a | a | a | a | a | a | a | a | a | b | b | a | a | b | a | a | b | a | b | a | a | a | a | a | a | a | 60 | 20 |
| a | a | a | a | a | a | a | a | a | b | b | a | a | b | a | a | b | a | b | a | a | a | b | a | a | a | 57 | 23 |
| a | a | a | a | a | a | a | a | a | b | b | a | a | b | a | a | b | a | b | a | a | a | b | a | a | a | 57 | 23 |
| a | a | a | a | a | a | b | a | a | a | b | a | a | b | a | a | b | a | b | a | a | a | b | a | a | a | 55 | 24 |
| a | a | a | a | a | a | b | a | a | a | b | a | a | b | a | a | b | a | b | a | a | a | b | a | a | a | 54 | 25 |
| a | a | a | a | a | a | b | a | a | a | b | a | a | b | a | a | b | a | b | a | a | a | b | a | a | a | 54 | 26 |
| a | a | a | a | a | a | b | b | a | a | b | a | a | b | a | a | b | a | b | a | a | a | b | a | a | a | 51 | 29 |
| a | a | a | a | a | a | b | b | a | a | b | a | a | b | a | a | b | a | b | a | a | b | b | a | a | a | 50 | 30 |
| a | a | a | a | b | a | b | b | a | a | b | a | a | b | a | a | b | a | b | a | a | b | b | a | a | a | 50 | 30 |
| b | b | b | a | b | a | a | b | b | a | a | b | a | b | b | b | b | b | a | b | a | a | a | b | b | a | 36 | 43 |
| b | b | b | a | b | a | a | b | b | a | a | b | a | b | b | b | b | b | a | b | a | a | a | b | b | a | 36 | 43 |
| b | b | b | a | b | a | a | b | b | a | b | b | a | b | b | b | b | b | a | b | a | a | a | b | b | a | 36 | 43 |
| b | b | b | b | b | a | a | b | b | a | b | b | a | b | b | b | b | b | a | b | a | a | a | b | b | a | 34 | 46 |
| b | b | b | b | b | b | a | b | b | a | b | b | a | b | a | b | b | b | a | b | a | a | b | b | b | a | 33 | 47 |
| b | b | b | b | b | b | a | b | b | a | b | b | a | b | a | b | b | b | a | a | a | a | b | b | b | a | 32 | 47 |
| b | b | b | b | b | b | a | b | b | a | b | b | a | b | a | b | b | b | a | a | a | a | b | b | b | a | 34 | 45 |
| b | b | b | b | b | b | a | b | b | a | b | b | a | b | a | b | b | a | b | a | a | a | b | b | b | a | 32 | 47 |
| b | b | b | b | b | b | a | b | b | a | b | b | a | b | a | b | b | a | b | a | a | a | b | b | b | a | 32 | 47 |
| b | b | b | b | b | b | a | b | b | a | b | b | a | b | a | b | b | a | b | a | a | a | b | b | b | a | 33 | 46 |
| b | b | b | b | b | b | a | b | b | a | b | b | a | b | a | b | b | a | b | a | a | a | b | b | b | a | 33 | 46 |
| b | b | b | b | b | a | a | b | b | a | b | b | a | b | a | b | b | a | b | a | a | a | b | b | b | a | 34 | 45 |
| b | b | b | b | b | a | a | b | b | a | b | b | a | b | a | b | b | a | b | a | a | a | b | b | b | a | 34 | 45 |
| b | b | b | b | b | a | a | b | b | a | b | b | a | b | a | b | b | a | b | a | a | a | b | b | b | a | 34 | 45 |
| b | b | b | b | b | a | a | b | b | a | b | b | a | b | a | b | b | a | b | a | a | a | b | b | b | a | 33 | 46 |
| b | b | b | b | b | a | a | b | b | a | b | b | a | b | a | b | b | a | a | a | a | a | b | b | b | a | 34 | 45 |
| b | b | b | b | b | b | a | b | b | a | b | b | a | b | a | b | b | a | a | a | b | a | b | b | b | b | 35 | 44 |
| b | a | b | b | b | b | b | b | b | a | b | b | a | b | a | b | b | a | a | a | b | a | b | b | b | b | 36 | 43 |
| b | b | a | b | b | b | b | b | a | a | b | a | a | b | a | b | b | a | b | a | a | a | a | b | b | b | 42 | 38 |
| b | b | a | b | b | a | b | b | a | a | b | a | a | b | a | b | b | a | b | a | a | a | a | b | b | b | 43 | 36 |
| a | b | b | a | a | b | a | a | a | a | b | a | b | b | b | b | a | a | a | b | b | b | a | b | b | a | 39 | 40 |
| a | b | b | a | a | b | a | a | a | a | b | a | b | b | b | b | a | a | a | b | b | b | a | b | b | a | 39 | 40 |
| a | b | b | a | a | b | a | a | a | a | b | a | b | b | b | b | a | a | a | b | b | b | a | a | b | a | 41 | 38 |
| a | a | a | a | a | b | b | a | a | a | b | a | b | b | b | b | a | a | a | b | a | b | a | a | b | a | 45 | 34 |
| b | a | a | a | a | b | b | a | a | a | b | a | b | b | b | b | a | a | a | b | a | b | a | b | b | a | 44 | 35 |
| b | a | a | a | a | b | b | a | b | a | b | a | b | b | b | b | a | a | a | b | a | b | a | b | b | a | 46 | 33 |
| b | a | a | a | a | b | b | a | b | a | b | a | b | b | b | b | a | a | a | b | a | b | a | b | b | a | 45 | 34 |
| b | a | a | b | a | b | b | a | b | a | b | a | b | b | b | b | a | a | a | b | b | b | a | b | b | a | 41 | 38 |
| b | a | a | b | a | b | b | a | b | a | b | a | b | b | b | b | a | b | a | b | b | b | a | b | b | a | 41 | 38 |
| b | a | a | b | a | b | b | a | b | a | b | a | b | b | b | b | a | b | a | b | b | b | a | b | b | a | 40 | 39 |
| b | a | a | b | a | b | b | a | b | a | b | a | b | b | b | b | a | b | a | b | b | b | a | b | b | a | 40 | 39 |
| b | a | a | b | a | b | b | a | b | a | b | a | b | b | b | b | a | b | a | b | b | b | a | b | b | a | 41 | 38 |
| b | a | a | b | a | b | b | a | b | a | b | a | b | b | b | b | a | b | a | b | b | b | a | b | b | a | 40 | 39 |
| b | a | a | b | a | b | b | a | b | a | b | a | b | b | b | b | a | b | a | b | b | b | a | b | b | a | 40 | 39 |
| b | a | a | b | a | b | b | a | b | a | b | a | b | b | b | b | a | b | a | b | b | b | a | b | b | a | 40 | 39 |
| b | a | a | b | a | b | b | a | a | a | b | a | b | b | b | b | a | b | a | b | b | b | a | b | b | a | 40 | 39 |
| b | a | a | b | a | a | b | a | a | a | b | a | b | a | b | a | a | b | b | a | b | b | a | b | b | b | 45 | 34 |
| b | a | a | b | a | a | b | a | a | a | b | a | b | a | b | a | a | b | a | a | b | b | a | b | a | b | 47 | 32 |
| b | a | a | a | a | a | b | a | a | a | b | a | b | a | b | b | a | b | a | a | b | b | a | b | a | b | 44 | 35 |
| b | a | a | a | a | a | b | a | a | a | b | a | b | a | b | b | a | b | a | a | b | b | a | b | a | b | 45 | 34 |
| a | b | a | a | a | a | b | a | a | a | b | b | a | a | a | a | a | a | a | a | a | b | a | a | a | b | 43 | 36 |
| a | a | a | b | a | a | a | a | a | b | b | b | a | a | a | b | a | a | a | a | a | b | a | a | b | a | 39 | 40 |
| b | a | a | b | a | a | a | a | a | b | b | b | a | b | a | b | a | a | a | a | b | b | a | b | b | a | 36 | 43 |
| b | a | a | b | a | a | a | a | a | b | a | a | a | a | a | b | a | b | a | b | b | b | b | b | b | a | 36 | 43 |
| b | a | b | b | a | a | a | a | a | b | a | a | a | b | a | b | a | b | a | b | b | b | b | b | b | a | 35 | 44 |
| b | a | b | b | a | a | a | a | a | b | b | a | a | b | a | b | a | b | a | b | b | b | b | b | b | a | 34 | 45 |
| b | a | b | b | a | a | a | a | a | b | b | a | a | b | a | b | a | b | a | b | b | b | b | b | b | a | 34 | 45 |
| b | a | b | b | a | a | a | a | a | b | b | a | a | b | a | b | a | b | a | b | b | b | b | b | b | a | 36 | 43 |
| b | a | b | b | a | a | a | a | a | b | b | a | a | b | a | b | a | b | a | b | b | b | b | b | b | a | 36 | 43 |
| b | a | b | b | a | a | a | a | a | b | b | a | a | b | a | b | a | b | a | b | b | b | b | b | b | a | 34 | 45 |
| b | a | b | b | a | a | a | a | a | b | b | a | a | b | a | b | a | b | a | b | b | b | b | b | b | a | 33 | 46 |
| b | a | b | b | a | a | a | a | a | b | b | a | a | b | a | b | a | b | a | b | b | b | b | b | b | a | 33 | 46 |
| b | a | b | b | a | a | a | a | a | b | b | a | a | b | a | b | a | b | a | b | b | b | b | b | b | a | 33 | 46 |
| b | a | b | b | a | a | a | a | a | b | b | a | a | b | a | b | a | b | a | b | b | b | b | b | b | a | 33 | 46 |
| b | a | b | b | a | a | a | a | a | b | b | a | a | b | a | b | a | b | a | b | b | b | b | b | b | a | 31 | 48 |
| b | a | b | b | a | a | a | a | a | b | b | a | a | b | a | b | a | b | a | b | b | a | b | b | b | a | 32 | 47 |
| b | a | b | b | a | a | a | a | a | b | b | a | a | b | a | b | a | b | a | b | b | a | b | b | b | a | 33 | 46 |
| b | a | b | b | a | a | a | a | a | b | b | a | a | b | a | b | a | b | a | b | b | a | b | b | b | b | 31 | 48 |
| b | a | b | b | a | a | a | a | a | a | b | a | a | b | a | b | b | b | b | b | b | a | b | b | b | b | 33 | 46 |
| b | a | b | b | a | a | a | a | a | a | b | a | a | b | a | b | b | b | b | b | b | a | b | b | b | b | 33 | 46 |
| b | a | a | a | a | a | a | a | b | a | a | a | b | a | b | a | a | b | a | a | b | a | b | a | a | a | 41 | 38 |
| b | a | a | a | b | a | a | a | b | a | a | a | b | a | b | a | a | b | a | a | b | a | b | a | a | a | 39 | 40 |
| b | a | a | a | b | a | a | a | b | a | a | a | b | a | b | a | a | b | a | a | b | a | b | a | a | a | 38 | 41 |
| b | a | a | b | b | a | a | a | b | a | a | a | b | a | b | a | b | b | a | b | b | a | b | a | a | a | 36 | 43 |
| b | a | a | b | b | a | a | a | b | a | a | a | b | a | b | a | b | b | a | b | b | a | b | a | a | a | 34 | 45 |
| b | a | a | b | b | a | a | a | b | a | a | a | b | a | b | a | b | b | a | b | b | a | b | a | a | a | 34 | 45 |
| b | a | a | b | b | a | a | a | b | a | a | a | b | a | b | a | b | b | a | b | b | a | b | a | a | a | 36 | 43 |
| b | b | a | b | b | a | a | a | b | a | a | a | b | a | b | a | b | b | a | b | b | b | b | a | a | a | 33 | 46 |
| b | b | a | b | b | a | a | a | b | a | a | a | b | a | b | a | b | b | a | b | b | b | b | a | a | a | 33 | 46 |
| b | b | a | b | b | a | a | a | b | a | a | a | b | a | b | a | b | b | a | b | b | b | b | a | a | a | 32 | 47 |
| b | b | a | b | b | a | a | b | b | a | a | a | b | a | b | a | b | b | a | b | b | b | b | a | a | a | 33 | 46 |
| b | b | a | b | b | a | a | b | b | a | a | a | b | a | b | a | b | b | a | b | b | b | b | a | a | a | 34 | 45 |
| b | b | a | b | b | a | a | b | b | a | a | a | b | a | b | a | b | b | a | b | b | b | b | a | b | a | 34 | 45 |
| b | b | a | b | a | a | a | b | b | a | a | a | b | a | b | a | b | b | a | b | b | b | b | a | b | a | 35 | 44 |
| b | b | a | a | a | a | a | b | b | a | a | a | b | a | b | a | b | b | a | b | b | b | b | a | b | a | 37 | 42 |
| b | b | a | a | a | a | a | b | b | b | a | a | b | a | b | a | b | a | a | b | b | b | b | a | b | b | 36 | 43 |
| b | b | a | a | a | a | a | b | b | b | a | a | b | a | b | a | b | a | a | a | b | b | b | a | b | b | 36 | 43 |
| b | b | a | a | a | a | b | b | b | b | b | a | b | a | b | b | b | a | b | a | b | b | b | a | b | b | 31 | 48 |
| b | a | a | a | a | a | b | b | b | b | b | b | b | a | b | b | b | a | b | a | a | b | b | b | b | b | 33 | 46 |
| b | a | a | a | a | a | b | b | b | b | a | b | b | a | b | b | a | a | b | a | a | b | b | b | b | b | 36 | 43 |
| b | a | a | a | a | a | b | b | b | b | a | b | b | a | b | b | a | a | b | a | a | b | b | b | b | b | 36 | 43 |
| a | a | a | b | a | a | b | a | a | a | a | a | a | a | a | a | b | a | b | b | b | a | b | a | a | b | 49 | 30 |
| a | a | a | b | a | a | b | a | a | a | a | a | a | a | a | a | b | a | b | b | b | a | b | a | a | b | 50 | 29 |
| a | a | a | b | a | a | b | a | a | a | a | a | a | a | a | a | b | a | b | b | b | a | b | a | a | b | 49 | 30 |
| a | a | a | b | a | a | b | a | a | a | a | a | a | a | a | b | b | a | b | b | b | a | b | a | a | b | 51 | 28 |
| a | a | a | b | a | a | b | a | a | a | a | a | a | a | a | b | b | a | b | b | b | a | b | a | a | b | 50 | 29 |
| a | a | a | b | a | a | b | a | a | a | a | a | a | a | a | b | b | a | b | b | b | a | b | a | a | b | 51 | 28 |
| a | a | a | b | a | a | b | a | a | a | a | a | a | a | a | b | b | a | b | b | b | a | b | a | a | b | 52 | 27 |
| a | a | a | b | a | a | a | a | a | a | a | a | a | a | a | b | b | b | b | b | b | b | b | a | a | b | 47 | 32 |
| a | a | a | b | a | a | a | a | a | a | a | a | a | a | a | b | b | b | b | b | b | b | a | a | a | b | 50 | 29 |
| a | a | a | b | a | a | a | a | a | a | a | a | a | a | a | b | b | b | b | b | b | b | a | a | a | a | 50 | 29 |
| a | a | b | b | a | a | a | a | a | a | a | a | b | b | a | b | b | b | b | b | b | b | a | a | a | a | 48 | 31 |
| a | a | b | b | a | a | a | a | a | a | a | a | b | b | a | b | b | b | b | b | b | b | a | a | a | a | 49 | 30 |
| a | b | b | a | a | b | a | a | a | a | a | a | b | b | a | b | b | b | b | b | b | b | a | a | a | a | 45 | 34 |
| a | b | b | a | a | b | a | a | a | a | a | a | b | b | a | b | b | b | b | b | b | b | a | a | a | a | 44 | 35 |
| a | b | b | a | a | a | a | b | a | a | a | a | b | b | a | b | b | b | a | b | b | b | a | a | b | a | 45 | 34 |
| a | b | b | a | a | a | a | b | a | b | a | a | b | b | a | b | b | b | a | b | b | b | a | a | b | a | 41 | 38 |
| a | b | a | a | a | a | a | a | a | b | a | a | b | b | a | b | a | b | a | b | b | b | a | a | b | a | 45 | 34 |
| a | b | a | a | b | b | b | a | a | b | a | a | b | b | a | a | a | b | a | a | b | b | a | a | b | a | 43 | 36 |
| a | b | b | a | b | b | a | a | a | b | a | a | b | b | b | a | a | b | a | a | b | b | a | a | b | a | 43 | 36 |
| a | b | b | a | b | b | a | a | a | a | a | a | b | b | b | a | a | b | a | b | b | b | a | a | b | a | 43 | 36 |
| 42 | 79 | 65 | 49 | 82 | 90 | 82 | 85 | 70 | 84 | 48 | 95 | 67 | 42 | 74 | 44 | 50 | 55 | 78 | 52 | 47 | 57 | 53 | 59 | 48 | 94 | | |
| 79 | 42 | 56 | 72 | 39 | 31 | 39 | 36 | 51 | 37 | 73 | 26 | 54 | 79 | 47 | 77 | 71 | 66 | 43 | 69 | 74 | 64 | 68 | 62 | 73 | 27 | | |
| 0.532 | 1.881 | 1.161 | 0.681 | 2.103 | 2.903 | 2.103 | 2.361 | 1.373 | 2.27 | 0.658 | 3.654 | 1.241 | 0.532 | 1.574 | 0.571 | 0.704 | 0.833 | 1.814 | 0.754 | 0.635 | 0.891 | 0.779 | 0.952 | 0.658 | 3.481 | | |

| marker segregation ratio N2/CB4856 |
|---|
| 1.9630 |
| 2.0769 |
| 8.8750 |
| 10.2857 |
| 5.0769 |
| 3.0000 |
| 3.2105 |
| 3.3889 |
| 3.2105 |
| 3.1579 |
| 3.2105 |
| 3.0000 |
| 2.4783 |
| 2.4783 |
| 2.2917 |
| 2.1600 |
| 2.0769 |
| 1.7586 |
| 1.6667 |
| 1.6667 |
| 0.8372 |
| 0.8372 |
| 0.8372 |
| 0.7391 |
| 0.7021 |
| 0.6809 |
| 0.7556 |
| 0.6809 |
| 0.6809 |
| 0.7174 |
| 0.7174 |
| 0.7556 |
| 0.7556 |
| 0.7556 |
| 0.7174 |
| 0.7556 |
| 0.7955 |
| 0.8372 |
| 1.1053 |
| 1.1944 |
| 0.9750 |
| 0.9750 |
| 1.0789 |
| 1.3235 |
| 1.2571 |
| 1.3939 |
| 1.3235 |
| 1.0789 |
| 1.0789 |
| 1.0256 |
| 1.0256 |
| 1.0789 |
| 1.0256 |
| 1.0256 |
| 1.0256 |
| 1.0256 |
| 1.3235 |
| 1.4688 |
| 1.2571 |
| 1.3235 |
| 1.1944 |
| 0.9750 |
| 0.8372 |
| 0.8372 |
| 0.7955 |
| 0.7556 |
| 0.7556 |
| 0.8372 |
| 0.8372 |
| 0.7556 |
| 0.7174 |
| 0.7174 |
| 0.7174 |
| 0.7174 |
| 0.6458 |
| 0.6809 |
| 0.7174 |
| 0.6458 |
| 0.7174 |
| 0.7174 |
| 1.0789 |
| 0.9750 |
| 0.9268 |
| 0.8372 |
| 0.7556 |
| 0.7556 |
| 0.8372 |
| 0.7174 |
| 0.7174 |
| 0.6809 |
| 0.7174 |
| 0.7556 |
| 0.7556 |
| 0.7955 |
| 0.8810 |
| 0.8372 |
| 0.8372 |
| 0.6458 |
| 0.7174 |
| 0.8372 |
| 0.8372 |
| 1.6333 |
| 1.7241 |
| 1.6333 |
| 1.8214 |
| 1.7241 |
| 1.8214 |
| 1.9259 |
| 1.4688 |
| 1.7241 |
| 1.7241 |
| 1.5484 |
| 1.6333 |
| 1.3235 |
| 1.2571 |
| 1.3235 |
| 1.0789 |
| 1.3235 |
| 1.1944 |
| 1.1944 |
| 1.1944 |
