# Supplementary material for: Mapping Determinants of Gene Expression Plasticity by Genetical Genomics in C. elegans
Source: PLoS Genet. 2006 Dec 29;2(12):e222. doi: 10.1371/journal.pgen.0020222 (PMC1756913; doi:10.1371/journal.pgen.0020222)
Supplement: Table S3 — (173 KB PDF) [file pgen.0020222.st003.pdf]

Yellow = p<0.05; Orange = p<0.01; Red = p <0.001

Marker association frequencies between markers on the same and on different chromosomes

Comparisons within chromosomes are chi2 tested against an expected recombination frequency calculated using the Haldane mapping function with twice the distance in cM as input. Comparisons between markers on different chromosomes are tested for significant deviation from an expected marker association using a chi2 test. The expected association was calculated with the formula Z = N*(x+y-2yx), in which Z is the expected association, N the total sample size, and x and y the marker frequencies on positions x and y, both for markers originating from the same parent. N2 marker frequencies are hidden in row 5 and column C. Yellow indicates a significant deviation at p<0.05, orange at p<0.01, red at p<0.001.

Vs.		N2 freq.	I 1	I 2	I 3	I 4	I 5	I 6	I 7	I 8	I 9	I 10	I 11	I 12	I 13	I 14	I 15	I 16	I 17	I 18	I 19	I 20
N2 marker freq.			0.68	0.69	0.89	0.91	0.82	0.73	0.75	0.76	0.75	0.74	0.75	0.73	0.69	0.69	0.68	0.66	0.65	0.61	0.60	0.60
	1	0.68	-	1.3	17.1	29.7	35.1	34.7	36.0	33.8	36.0	35.1	33.3	37.3	41.3	41.3	41.9	43.2	48.0	52.0	53.3	53.3
	2	0.69		-	15.7	28.4	33.8	36.0	37.3	35.1	37.3	36.5	34.7	38.7	42.7	42.7	43.2	44.6	49.3	53.3	54.7	54.7
	3	0.89			-	14.3	24.3	30.0	30.0	28.6	30.0	30.0	30.0	34.3	38.6	38.6	40.0	41.4	42.9	47.1	48.6	48.6
	4	0.91				-	10.8	21.6	21.6	23.0	24.3	24.3	24.3	28.4	32.4	32.4	33.8	35.1	39.2	43.2	44.6	44.6
	5	0.82					-	10.8	10.8	12.2	13.5	13.5	16.2	20.3	21.6	21.6	25.7	27.0	31.1	35.1	36.5	36.5
	6	0.73						-	1.3	1.4	4.0	2.7	6.7	10.7	12.0	12.0	14.9	16.2	21.3	25.3	26.7	29.3
	7	0.75							-	1.4	2.7	2.7	5.3	9.3	10.7	10.7	14.9	16.2	20.0	24.0	25.3	28.0
	8	0.76								-	1.4	1.4	4.1	8.1	9.5	9.5	13.5	14.9	18.9	23.0	24.3	27.0
	9	0.75									-	0.0	2.7	6.7	8.0	8.0	12.2	13.5	17.3	21.3	22.7	25.3
I	10	0.74										-	2.7	6.8	8.1	8.1	12.2	13.5	17.6	21.6	23.0	25.7
	11	0.75											-	4.0	8.0	8.0	12.2	13.5	17.3	21.3	22.7	25.3
	12	0.73												-	4.0	4.0	8.1	9.5	13.3	20.0	21.3	24.0
	13	0.69													-	0.0	4.1	5.4	9.3	16.0	17.3	20.0
	14	0.69														-	4.1	5.4	9.3	16.0	17.3	20.0
	15	0.68															-	1.4	5.4	12.2	13.5	16.2
	16	0.66																-	4.1	10.8	12.2	14.9
	17	0.65																	-	6.7	8.0	10.7
	18	0.61																		-	1.3	4.0
	19	0.60																			-	2.7
	20	0.60																				-
	1	0.46																				
	2	0.46																				
	3	0.46																				
	4	0.43																				
	5	0.41																				
	6	0.41																				
	7	0.43																				
	8	0.41																				
	9	0.41																				
II	10	0.42																				
	11	0.42																				
	12	0.43																				
	13	0.43																				
	14	0.43																				
	15	0.42																				
	16	0.43																				
	17	0.47																				
	18	0.49																				
	19	0.56																				
	20	0.58																				
	1	0.50																				
	2	0.50																				
	3	0.53																				
	4	0.58																				
	5	0.57																				
	6	0.59																				
	7	0.58																				
	8	0.53																				
	9	0.53																				
III	10	0.51																				
	11	0.51																				
	12	0.53																				
	13	0.51																				
	14	0.51																				
	15	0.51																				
	16	0.51																				
	17	0.58																				
	18	0.59																				
	19	0.58																				
	20	0.59																				
	1	0.54																				
	2	0.50																				
	3	0.46																				
	4	0.46																				
	5	0.45																				
	6	0.43																				
	7	0.43																				
	8	0.46																				
	9	0.46																				
IV	10	0.43																				
	11	0.42																				
	12	0.42																				
	13	0.42																				
	14	0.42																				
	15	0.39																				
	16	0.41																				
	17	0.42																				
	18	0.42																				
	19	0.45																				
	20	0.45																				
	1	0.49																				
	2	0.46																				
	3	0.45																				
	4	0.42																				
	5	0.39																				
	6	0.39																				
	7	0.42																				
	8	0.38																				
	9	0.38																				
	10	0.36																				
V	11	0.38																				
	12	0.39																				
	13	0.41																				
	14	0.42																				
	15	0.45																				
	16	0.46																				
	17	0.46																				
	18	0.42																				
	19	0.45																				
	20	0.49																				
	21	0.49																				
	1	0.62																				
	2	0.64																				
	3	0.62																				
	4	0.65																				
	5	0.65																				
	6	0.66																				
	7	0.68																				
	8	0.61																				
	9	0.65																				
X	10	0.62																				
	11	0.59																				
	12	0.61																				
	13	0.55																				
	14	0.54																				
	15	0.57																				
	16	0.51																				
	17	0.58																				
	18	0.54																				
	19	0.53																				
	20	0.53																				

II

1	2	3	4	5	6	7	8	9	10	11	12	13	14	15	16	17	18	19	20
0.46	0.46	0.46	0.43	0.41	0.41	0.43	0.41	0.41	0.42	0.42	0.43	0.43	0.43	0.42	0.43	0.47	0.49	0.56	0.58
44.6	44.6	44.6	44.6	48.6	50.0	47.3	47.3	44.6	43.2	43.2	41.9	41.9	41.9	43.2	44.6	48.6	47.3	47.3	45.9
45.9	45.9	45.9	45.9	50.0	51.4	48.6	48.6	45.9	44.6	44.6	43.2	43.2	43.2	44.6	45.9	50.0	45.9	45.9	44.6
51.4	51.4	51.4	51.4	54.3	55.7	52.9	54.3	54.3	52.9	52.9	51.4	51.4	51.4	52.9	51.4	51.4	48.6	44.3	42.9
47.3	47.3	47.3	50.0	54.1	55.4	55.4	52.7	52.7	51.4	51.4	50.0	50.0	50.0	51.4	50.0	48.6	44.6	44.6	43.2
44.6	44.6	44.6	52.7	54.1	55.4	55.4	52.7	55.4	54.1	54.1	52.7	52.7	52.7	54.1	52.7	54.1	50.0	50.0	48.6
50.0	50.0	47.3	52.7	54.1	55.4	52.7	52.7	55.4	54.1	54.1	52.7	52.7	52.7	54.1	52.7	51.4	50.0	50.0	48.6
50.0	50.0	47.3	52.7	54.1	55.4	52.7	52.7	55.4	54.1	54.1	52.7	52.7	52.7	54.1	52.7	51.4	50.0	50.0	48.6
51.4	51.4	48.6	54.1	55.4	56.8	54.1	54.1	56.8	55.4	55.4	54.1	54.1	54.1	55.4	54.1	52.7	51.4	51.4	50.0
50.0	50.0	47.3	52.7	54.1	55.4	52.7	52.7	55.4	54.1	54.1	52.7	52.7	52.7	54.1	52.7	51.4	50.0	50.0	48.6
50.0	50.0	47.3	52.7	54.1	55.4	52.7	52.7	55.4	54.1	54.1	52.7	52.7	52.7	54.1	52.7	51.4	50.0	50.0	48.6
50.0	50.0	47.3	52.7	54.1	55.4	52.7	50.0	52.7	51.4	51.4	50.0	50.0	50.0	51.4	52.7	51.4	50.0	47.3	45.9
51.4	51.4	48.6	54.1	55.4	54.1	54.1	51.4	54.1	52.7	52.7	51.4	51.4	51.4	52.7	51.4	50.0	51.4	45.9	44.6
55.4	55.4	52.7	55.4	54.1	52.7	52.7	50.0	52.7	51.4	51.4	50.0	50.0	50.0	51.4	50.0	48.6	50.0	50.0	48.6
55.4	55.4	52.7	55.4	54.1	52.7	52.7	50.0	52.7	51.4	51.4	50.0	50.0	50.0	51.4	50.0	48.6	50.0	50.0	48.6
56.8	56.8	54.1	56.8	52.7	51.4	51.4	48.6	51.4	50.0	50.0	48.6	48.6	48.6	50.0	48.6	47.3	45.9	45.9	44.6
58.1	58.1	55.4	58.1	54.1	52.7	52.7	50.0	52.7	51.4	51.4	50.0	50.0	50.0	51.4	50.0	48.6	47.3	47.3	45.9
62.2	62.2	59.5	62.2	58.1	56.8	56.8	54.1	56.8	55.4	55.4	54.1	54.1	54.1	55.4	54.1	50.0	51.4	51.4	50.0
63.5	63.5	60.8	66.2	59.5	58.1	55.4	52.7	55.4	54.1	54.1	52.7	52.7	52.7	54.1	55.4	54.1	55.4	50.0	48.6
64.9	64.9	62.2	67.6	60.8	59.5	56.8	54.1	56.8	55.4	55.4	54.1	54.1	54.1	55.4	56.8	55.4	56.8	51.4	50.0
62.2	62.2	59.5	67.6	60.8	59.5	56.8	54.1	56.8	55.4	55.4	54.1	54.1	54.1	55.4	56.8	55.4	56.8	48.6	47.3
-	0.0	2.7	13.5	23.0	24.3	27.0	29.7	32.4	33.8	33.8	32.4	32.4	32.4	31.1	29.7	39.2	40.5	45.9	44.6
	-	2.7	13.5	23.0	24.3	27.0	29.7	32.4	33.8	33.8	32.4	32.4	32.4	31.1	29.7	39.2	40.5	45.9	44.6
		-	10.8	20.3	24.3	27.0	29.7	32.4	33.8	33.8	32.4	32.4	32.4	31.1	29.7	39.2	40.5	45.9	44.6
			-	9.3	13.5	16.2	18.9	21.6	23.0	23.0	21.6	21.6	21.6	20.3	18.9	28.4	29.7	40.0	39.2
				-	4.1	6.8	9.5	12.2	13.5	13.5	14.9	14.9	14.9	13.5	12.2	21.6	23.0	33.3	35.1
					-	2.7	5.4	8.1	9.5	9.5	10.8	10.8	10.8	9.5	8.1	17.6	21.6	32.4	33.8
						-	5.4	8.1	9.5	9.5	10.8	10.8	10.8	9.5	10.8	20.3	24.3	32.4	33.8
							-	2.7	4.1	4.1	5.4	5.4	5.4	4.1	8.1	17.6	21.6	32.4	33.8
								-	1.4	1.4	2.7	2.7	2.7	1.4	5.4	14.9	18.9	29.7	31.1
									-	0.0	1.4	1.4	1.4	2.7	6.8	16.2	20.3	31.1	32.4
										-	1.4	1.4	1.4	2.7	6.8	16.2	20.3	31.1	32.4
											-	0.0	0.0	1.4	5.4	17.6	21.6	32.4	31.1
												-	0.0	1.4	5.4	17.6	21.6	32.4	31.1
													-	1.4	5.4	17.6	21.6	32.4	31.1
														-	4.1	16.2	20.3	31.1	29.7
															-	12.2	16.2	29.7	28.4
																-	6.8	25.7	27.0
																	-	21.6	23.0
																		-	1.4
																			-

III

1	2	3	4	5	6	7	8	9	10	11	12	13	14	15	16	17	18	19	20
0.50	0.50	0.53	0.58	0.57	0.59	0.58	0.53	0.53	0.51	0.51	0.53	0.51	0.51	0.51	0.51	0.58	0.59	0.58	0.59
51.4	51.4	54.1	56.8	52.7	50.0	48.6	48.6	48.6	47.3	47.3	48.6	47.3	47.3	50.0	50.0	43.2	47.3	48.6	47.3
50.0	50.0	52.7	55.4	51.4	48.6	47.3	50.0	47.3	48.6	48.6	50.0	48.6	48.6	48.6	48.6	41.9	45.9	47.3	45.9
47.1	47.1	50.0	48.6	44.3	41.4	40.0	45.7	45.7	47.1	47.1	48.6	47.1	47.1	47.1	45.7	42.9	41.4	40.0	38.6
54.1	54.1	51.4	51.4	50.0	47.3	48.6	54.1	54.1	55.4	55.4	54.1	55.4	55.4	52.7	50.0	40.5	39.2	40.5	39.2
48.6	48.6	45.9	48.6	44.6	41.9	43.2	48.6	48.6	50.0	50.0	48.6	50.0	50.0	47.3	44.6	40.5	39.2	40.5	39.2
43.2	43.2	40.5	43.2	39.2	39.2	40.5	43.2	45.9	44.6	44.6	45.9	44.6	44.6	47.3	44.6	45.9	47.3	45.9	44.6
43.2	43.2	40.5	43.2	39.2	39.2	40.5	43.2	45.9	44.6	44.6	45.9	44.6	44.6	47.3	44.6	45.9	47.3	45.9	44.6
41.9	41.9	39.2	41.9	40.5	40.5	41.9	44.6	47.3	45.9	45.9	47.3	45.9	45.9	48.6	45.9	47.3	48.6	47.3	45.9
43.2	43.2	40.5	43.2	41.9	41.9	43.2	45.9	48.6	47.3	47.3	48.6	47.3	47.3	50.0	47.3	48.6	50.0	48.6	47.3
43.2	43.2	40.5	43.2	41.9	41.9	43.2	45.9	48.6	47.3	47.3	48.6	47.3	47.3	50.0	47.3	48.6	50.0	48.6	47.3
45.9	45.9	43.2	45.9	41.9	41.9	43.2	45.9	48.6	47.3	47.3	48.6	47.3	47.3	50.0	47.3	45.9	50.0	48.6	47.3
47.3	47.3	44.6	44.6	40.5	40.5	41.9	44.6	47.3	45.9	45.9	47.3	45.9	45.9	48.6	45.9	44.6	48.6	47.3	45.9
48.6	48.6	45.9	45.9	44.6	44.6	45.9	48.6	51.4	50.0	50.0	51.4	50.0	50.0	52.7	50.0	48.6	52.7	51.4	50.0
48.6	48.6	45.9	45.9	44.6	44.6	45.9	48.6	51.4	50.0	50.0	51.4	50.0	50.0	52.7	50.0	48.6	52.7	51.4	50.0
50.0	50.0	47.3	44.6	43.2	43.2	44.6	44.6	47.3	45.9	45.9	47.3	45.9	45.9	48.6	45.9	44.6	48.6	47.3	45.9
51.4	51.4	48.6	45.9	41.9	41.9	43.2	43.2	45.9	44.6	44.6	45.9	44.6	44.6	47.3	44.6	43.2	47.3	45.9	44.6
50.0	50.0	47.3	44.6	40.5	40.5	41.9	41.9	44.6	43.2	43.2	44.6	43.2	43.2	45.9	45.9	44.6	48.6	47.3	45.9
51.4	51.4	48.6	45.9	41.9	41.9	43.2	43.2	45.9	44.6	44.6	45.9	44.6	44.6	47.3	47.3	43.2	47.3	45.9	44.6
50.0	50.0	47.3	44.6	40.5	40.5	41.9	41.9	44.6	43.2	43.2	44.6	43.2	43.2	45.9	45.9	41.9	45.9	44.6	43.2
52.7	52.7	50.0	47.3	43.2	43.2	44.6	44.6	47.3	45.9	45.9	44.6	45.9	45.9	48.6	48.6	41.9	45.9	47.3	45.9
50.0	50.0	52.7	55.4	48.6	51.4	50.0	52.7	52.7	51.4	51.4	50.0	51.4	51.4	51.4	51.4	55.4	51.4	52.7	51.4
50.0	50.0	52.7	55.4	48.6	51.4	50.0	52.7	52.7	51.4	51.4	50.0	51.4	51.4	51.4	51.4	55.4	51.4	52.7	51.4
50.0	50.0	52.7	55.4	48.6	51.4	50.0	52.7	52.7	51.4	51.4	50.0	51.4	51.4	51.4	51.4	52.7	48.6	50.0	48.6
52.7	52.7	55.4	58.1	51.4	54.1	52.7	52.7	52.7	51.4	51.4	52.7	51.4	51.4	51.4	51.4	55.4	51.4	52.7	51.4
56.8	56.8	59.5	62.2	52.7	55.4	54.1	51.4	51.4	50.0	50.0	51.4	50.0	50.0	50.0	50.0	56.8	52.7	54.1	52.7
55.4	55.4	58.1	60.8	51.4	54.1	52.7	50.0	50.0	48.6	48.6	50.0	48.6	48.6	48.6	48.6	55.4	51.4	52.7	51.4
52.7	52.7	55.4	58.1	48.6	51.4	50.0	47.3	47.3	45.9	45.9	47.3	45.9	45.9	48.6	48.6	55.4	51.4	52.7	51.4
52.7	52.7	55.4	60.8	51.4	54.1	52.7	50.0	52.7	51.4	51.4	52.7	51.4	51.4	51.4	51.4	55.4	54.1	55.4	54.1
50.0	50.0	52.7	58.1	54.1	56.8	55.4	52.7	55.4	54.1	54.1	55.4	54.1	54.1	54.1	54.1	58.1	56.8	58.1	56.8
48.6	48.6	51.4	56.8	52.7	55.4	54.1	51.4	54.1	52.7	52.7	54.1	52.7	52.7	52.7	52.7	56.8	58.1	59.5	58.1
48.6	48.6	51.4	56.8	52.7	55.4	54.1	51.4	54.1	52.7	52.7	54.1	52.7	52.7	52.7	52.7	56.8	58.1	59.5	58.1
50.0	50.0	52.7	58.1	54.1	56.8	55.4	52.7	55.4	54.1	54.1	55.4	54.1	54.1	54.1	54.1	55.4	56.8	58.1	56.8
50.0	50.0	52.7	58.1	54.1	56.8	55.4	52.7	55.4	54.1	54.1	55.4	54.1	54.1	54.1	54.1	55.4	56.8	58.1	56.8
50.0	50.0	52.7	58.1	54.1	56.8	55.4	52.7	55.4	54.1	54.1	55.4	54.1	54.1	54.1	54.1	55.4	56.8	58.1	56.8
51.4	51.4	54.1	59.5	55.4	58.1	56.8	54.1	56.8	55.4	55.4	56.8	55.4	55.4	55.4	55.4	56.8	55.4	56.8	55.4
52.7	52.7	55.4	60.8	56.8	59.5	58.1	55.4	58.1	56.8	56.8	58.1	56.8	56.8	56.8	56.8	58.1	54.1	55.4	54.1
51.4	51.4	51.4	56.8	52.7	55.4	56.8	51.4	54.1	52.7	52.7	54.1	52.7	52.7	52.7	55.4	59.5	58.1	59.5	58.1
55.4	55.4	55.4	55.4	51.4	54.1	55.4	52.7	52.7	54.1	54.1	55.4	54.1	54.1	51.4	51.4	55.4	54.1	55.4	54.1
58.1	58.1	58.1	52.7	48.6	54.1	55.4	55.4	55.4	56.8	56.8	55.4	56.8	56.8	56.8	54.1	50.0	51.4	55.4	54.1
59.5	59.5	59.5	54.1	50.0	55.4	56.8	56.8	56.8	58.1	58.1	56.8	58.1	58.1	58.1	55.4	48.6	50.0	54.1	52.7
-	0.0	5.4	13.5	20.3	25.7	27.0	29.7	29.7	31.1	31.1	32.4	31.1	31.1	31.1	31.1	43.2	41.9	40.5	41.9
	-	5.4	13.5	20.3	25.7	27.0	29.7	29.7	31.1	31.1	32.4	31.1	31.1	31.1	31.1	43.2	41.9	40.5	41.9
		-	10.8	20.3	28.4	29.7	32.4	32.4	33.8	33.8	35.1	33.8	33.8	33.8	33.8	45.9	47.3	45.9	47.3
			-	14.9	23.0	24.3	29.7	29.7	31.1	31.1	32.4	31.1	31.1	31.1	31.1	43.2	41.9	40.5	41.9
				-	8.1	9.5	14.9	14.9	16.2	16.2	17.6	16.2	16.2	16.2	16.2	28.4	32.4	31.1	29.7
					-	1.4	6.8	6.8	8.1	8.1	9.5	8.1	8.1	8.1	10.8	23.0	29.7	28.4	27.0
						-	5.4	5.4	6.8	6.8	8.1	6.8	6.8	6.8	9.5	24.3	31.1	29.7	28.4
							-	2.7	1.4	1.4	2.7	1.4	1.4	4.1	6.8	21.6	28.4	29.7	28.4
								-	1.4	1.4	2.7	1.4	1.4	1.4	4.1	18.9	25.7	27.0	25.7
									-	0.0	1.4	0.0	0.0	2.7	5.4	20.3	27.0	28.4	27.0
										-	1.4	0.0	0.0	2.7	5.4	20.3	27.0	28.4	27.0
											-	1.4	1.4	4.1	6.8	18.9	25.7	29.7	28.4
												-	0.0	2.7	5.4	20.3	27.0	28.4	27.0
													-	2.7	5.4	20.3	27.0	28.4	27.0
														-	2.7	17.6	24.3	25.7	24.3
															-	14.9	21.6	23.0	21.6
																-	9.5	13.5	12.2
																	-	4.1	5.4
																		-	1.4
																			-

IV

1	2	3	4	5	6	7	8	9	10	11	12	13	14	15	16	17	18	19	20
0.54	0.50	0.46	0.46	0.45	0.43	0.43	0.46	0.46	0.43	0.42	0.42	0.42	0.42	0.39	0.41	0.42	0.42	0.45	0.45
55.4	54.1	55.4	58.1	56.8	58.1	58.1	58.1	58.1	60.8	62.2	62.2	62.2	62.2	62.2	52.7	51.4	51.4	45.9	45.9
54.1	52.7	54.1	56.8	55.4	56.8	56.8	56.8	56.8	59.5	60.8	60.8	60.8	60.8	60.8	54.1	52.7	52.7	47.3	47.3
52.9	57.1	55.7	55.7	54.3	55.7	55.7	55.7	55.7	58.6	60.0	60.0	60.0	60.0	60.0	57.1	55.7	55.7	54.3	54.3
50.0	54.1	55.4	55.4	54.1	55.4	55.4	52.7	52.7	55.4	56.8	56.8	56.8	56.8	59.5	60.8	59.5	59.5	54.1	54.1
44.6	45.9	47.3	50.0	48.6	50.0	50.0	47.3	47.3	50.0	51.4	51.4	51.4	51.4	54.1	55.4	54.1	54.1	54.1	54.1
41.9	40.5	41.9	47.3	45.9	44.6	44.6	41.9	41.9	44.6	45.9	45.9	45.9	45.9	48.6	44.6	43.2	43.2	43.2	43.2
41.9	40.5	41.9	47.3	45.9	44.6	44.6	41.9	41.9	44.6	45.9	45.9	45.9	45.9	48.6	44.6	43.2	43.2	43.2	43.2
40.5	39.2	43.2	48.6	47.3	45.9	45.9	43.2	43.2	45.9	47.3	47.3	47.3	47.3	50.0	45.9	44.6	44.6	44.6	44.6
41.9	40.5	44.6	50.0	48.6	47.3	47.3	44.6	44.6	44.6	45.9	45.9	45.9	45.9	48.6	44.6	43.2	43.2	45.9	45.9
41.9	40.5	44.6	50.0	48.6	47.3	47.3	44.6	44.6	44.6	45.9	45.9	45.9	45.9	48.6	44.6	43.2	43.2	45.9	45.9
44.6	43.2	44.6	52.7	48.6	47.3	47.3	44.6	44.6	44.6	45.9	45.9	45.9	45.9	48.6	44.6	43.2	43.2	43.2	43.2
40.5	39.2	45.9	54.1	50.0	48.6	48.6	48.6	48.6	48.6	50.0	50.0	50.0	50.0	52.7	48.6	47.3	47.3	47.3	47.3
41.9	40.5	50.0	50.0	48.6	47.3	47.3	47.3	47.3	47.3	48.6	48.6	48.6	48.6	51.4	50.0	48.6	48.6	48.6	48.6
41.9	40.5	50.0	50.0	48.6	47.3	47.3	47.3	47.3	47.3	48.6	48.6	48.6	48.6	51.4	50.0	48.6	48.6	48.6	48.6
37.8	41.9	51.4	51.4	50.0	48.6	48.6	48.6	48.6	48.6	50.0	50.0	50.0	50.0	55.4	54.1	52.7	52.7	50.0	50.0
36.5	40.5	50.0	50.0	48.6	50.0	50.0	50.0	50.0	50.0	51.4	51.4	51.4	51.4	56.8	55.4	54.1	54.1	51.4	51.4
37.8	44.6	54.1	54.1	52.7	54.1	54.1	54.1	54.1	54.1	52.7	52.7	52.7	52.7	58.1	54.1	52.7	52.7	52.7	52.7
39.2	45.9	52.7	52.7	54.1	55.4	55.4	52.7	52.7	52.7	51.4	51.4	51.4	51.4	56.8	52.7	51.4	51.4	51.4	51.4
37.8	44.6	51.4	51.4	52.7	54.1	54.1	51.4	51.4	51.4	50.0	50.0	50.0	50.0	55.4	54.1	52.7	52.7	52.7	52.7
40.5	47.3	54.1	54.1	55.4	56.8	56.8	54.1	54.1	54.1	52.7	52.7	52.7	52.7	58.1	56.8	55.4	55.4	55.4	55.4
56.8	58.1	54.1	59.5	55.4	54.1	54.1	56.8	56.8	54.1	55.4	55.4	55.4	55.4	47.3	48.6	50.0	50.0	50.0	50.0
56.8	58.1	54.1	59.5	55.4	54.1	54.1	56.8	56.8	54.1	55.4	55.4	55.4	55.4	47.3	48.6	50.0	50.0	50.0	50.0
56.8	58.1	54.1	62.2	58.1	54.1	54.1	56.8	56.8	54.1	55.4	55.4	55.4	55.4	47.3	45.9	47.3	47.3	47.3	47.3
64.9	58.1	54.1	56.8	52.7	48.6	48.6	51.4	51.4	48.6	50.0	50.0	50.0	50.0	41.9	43.2	44.6	47.3	44.6	44.6
63.5	56.8	52.7	52.7	45.9	41.9	41.9	44.6	44.6	41.9	43.2	43.2	43.2	43.2	37.8	39.2	40.5	43.2	40.5	40.5
59.5	52.7	51.4	54.1	47.3	43.2	43.2	45.9	45.9	43.2	44.6	44.6	44.6	44.6	39.2	43.2	44.6	47.3	44.6	44.6
62.2	55.4	51.4	54.1	50.0	45.9	45.9	45.9	45.9	43.2	44.6	44.6	44.6	44.6	39.2	40.5	41.9	44.6	41.9	41.9
62.2	55.4	51.4	56.8	52.7	48.6	48.6	48.6	48.6	45.9	47.3	47.3	47.3	47.3	41.9	45.9	47.3	50.0	44.6	44.6
64.9	58.1	54.1	59.5	52.7	48.6	48.6	48.6	48.6	45.9	47.3	47.3	47.3	47.3	41.9	45.9	47.3	50.0	44.6	44.6
63.5	56.8	52.7	58.1	51.4	47.3	47.3	47.3	47.3	47.3	48.6	48.6	48.6	48.6	43.2	44.6	45.9	48.6	43.2	43.2
63.5	56.8	52.7	58.1	51.4	47.3	47.3	47.3	47.3	47.3	48.6	48.6	48.6	48.6	43.2	44.6	45.9	48.6	43.2	43.2
62.2	55.4	51.4	56.8	50.0	45.9	45.9	45.9	45.9	45.9	47.3	47.3	47.3	47.3	41.9	43.2	44.6	47.3	41.9	41.9
62.2	55.4	51.4	56.8	50.0	45.9	45.9	45.9	45.9	45.9	47.3	47.3	47.3	47.3	41.9	43.2	44.6	47.3	41.9	41.9
62.2	55.4	51.4	56.8	50.0	45.9	45.9	45.9	45.9	45.9	47.3	47.3	47.3	47.3	41.9	43.2	44.6	47.3	41.9	41.9
63.5	56.8	52.7	58.1	51.4	47.3	47.3	47.3	47.3	44.6	45.9	45.9	45.9	45.9	40.5	44.6	45.9	48.6	43.2	43.2
59.5	52.7	54.1	59.5	50.0	45.9	45.9	48.6	48.6	45.9	47.3	47.3	47.3	47.3	41.9	45.9	47.3	50.0	47.3	47.3
66.2	59.5	58.1	63.5	54.1	50.0	50.0	52.7	52.7	50.0	51.4	51.4	51.4	51.4	48.6	52.7	51.4	51.4	48.6	48.6
64.9	58.1	54.1	59.5	50.0	45.9	45.9	48.6	48.6	45.9	47.3	47.3	47.3	47.3	44.6	51.4	50.0	50.0	47.3	47.3
56.8	55.4	54.1	59.5	52.7	48.6	48.6	51.4	51.4	48.6	50.0	50.0	50.0	50.0	50.0	51.4	50.0	50.0	44.6	44.6
55.4	54.1	52.7	58.1	51.4	47.3	47.3	50.0	50.0	47.3	48.6	48.6	48.6	48.6	48.6	50.0	48.6	48.6	43.2	43.2
47.3	43.2	39.2	44.6	45.9	41.9	41.9	41.9	41.9	44.6	45.9	45.9	45.9	45.9	45.9	44.6	45.9	45.9	48.6	48.6
47.3	43.2	39.2	44.6	45.9	41.9	41.9	41.9	41.9	44.6	45.9	45.9	45.9	45.9	45.9	44.6	45.9	45.9	48.6	48.6
44.6	40.5	41.9	47.3	48.6	44.6	44.6	41.9	41.9	44.6	45.9	45.9	45.9	45.9	45.9	44.6	43.2	43.2	45.9	45.9
41.9	37.8	41.9	47.3	51.4	47.3	47.3	44.6	44.6	47.3	48.6	48.6	48.6	48.6	48.6	47.3	48.6	45.9	48.6	48.6
45.9	41.9	35.1	43.2	44.6	43.2	43.2	43.2	43.2	45.9	47.3	47.3	47.3	47.3	47.3	45.9	44.6	44.6	47.3	47.3
48.6	44.6	37.8	45.9	47.3	48.6	48.6	48.6	48.6	51.4	52.7	52.7	52.7	52.7	52.7	51.4	50.0	50.0	52.7	52.7
50.0	45.9	39.2	47.3	48.6	50.0	50.0	50.0	50.0	52.7	54.1	54.1	54.1	54.1	51.4	50.0	48.6	48.6	51.4	51.4
52.7	45.9	36.5	44.6	45.9	47.3	47.3	47.3	47.3	50.0	51.4	51.4	51.4	51.4	51.4	47.3	45.9	45.9	48.6	48.6
52.7	45.9	36.5	41.9	43.2	44.6	44.6	44.6	44.6	47.3	48.6	48.6	48.6	48.6	48.6	47.3	45.9	45.9	48.6	48.6
54.1	47.3	37.8	43.2	44.6	45.9	45.9	45.9	45.9	48.6	50.0	50.0	50.0	50.0	50.0	45.9	44.6	44.6	47.3	47.3
54.1	47.3	37.8	43.2	44.6	45.9	45.9	45.9	45.9	48.6	50.0	50.0	50.0	50.0	50.0	45.9	44.6	44.6	47.3	47.3
55.4	48.6	39.2	44.6	45.9	47.3	47.3	47.3	47.3	50.0	51.4	51.4	51.4	51.4	51.4	47.3	45.9	45.9	48.6	48.6
54.1	47.3	37.8	43.2	44.6	45.9	45.9	45.9	45.9	48.6	50.0	50.0	50.0	50.0	50.0	45.9	44.6	44.6	47.3	47.3
54.1	47.3	37.8	43.2	44.6	45.9	45.9	45.9	45.9	48.6	50.0	50.0	50.0	50.0	50.0	45.9	44.6	44.6	47.3	47.3
51.4	44.6	35.1	40.5	41.9	43.2	43.2	43.2	43.2	45.9	47.3	47.3	47.3	47.3	47.3	48.6	47.3	47.3	50.0	50.0
48.6	41.9	32.4	37.8	39.2	40.5	40.5	40.5	40.5	43.2	44.6	44.6	44.6	44.6	44.6	45.9	44.6	44.6	47.3	47.3
44.6	40.5	36.5	41.9	45.9	47.3	47.3	47.3	47.3	50.0	51.4	51.4	51.4	51.4	54.1	52.7	51.4	51.4	51.4	51.4
43.2	39.2	35.1	40.5	41.9	43.2	43.2	43.2	43.2	43.2	44.6	44.6	44.6	44.6	50.0	54.1	55.4	55.4	58.1	58.1
39.2	37.8	33.8	39.2	40.5	41.9	41.9	41.9	41.9	41.9	43.2	43.2	43.2	43.2	48.6	52.7	54.1	54.1	56.8	56.8
40.5	39.2	35.1	40.5	41.9	43.2	43.2	43.2	43.2	43.2	44.6	44.6	44.6	44.6	50.0	54.1	55.4	55.4	58.1	58.1
-	14.9	27.0	32.4	39.2	40.5	40.5	40.5	40.5	43.2	41.9	41.9	41.9	41.9	50.0	54.1	55.4	55.4	55.4	55.4
	-	14.9	23.0	29.7	31.1	31.1	31.1	31.1	33.8	35.1	35.1	35.1	35.1	43.2	44.6	45.9	45.9	48.6	48.6
		-	13.5	17.6	18.9	18.9	18.9	18.9	21.6	23.0	23.0	23.0	23.0	31.1	35.1	36.5	36.5	39.2	39.2
			-	9.5	13.5	13.5	13.5	13.5	16.2	17.6	17.6	17.6	17.6	25.7	32.4	33.8	36.5	39.2	39.2
				-	4.1	4.1	6.8	6.8	9.5	10.8	10.8	10.8	10.8	18.9	28.4	29.7	32.4	35.1	35.1
					-	0.0	2.7	2.7	5.4	6.8	6.8	6.8	6.8	14.9	24.3	25.7	28.4	31.1	31.1
						-	2.7	2.7	5.4	6.8	6.8	6.8	6.8	14.9	24.3	25.7	28.4	31.1	31.1
							-	0.0	2.7	4.1	4.1	4.1	4.1	12.2	21.6	23.0	25.7	28.4	28.4
								-	2.7	4.1	4.1	4.1	4.1	12.2	21.6	23.0	25.7	28.4	28.4
									-	1.4	1.4	1.4	1.4	9.5	21.6	23.0	25.7	31.1	31.1
										-	0.0	0.0	0.0	8.1	20.3	21.6	24.3	29.7	29.7
											-	0.0	0.0	8.1	20.3	21.6	24.3	29.7	29.7
												-	0.0	8.1	20.3	21.6	24.3	29.7	29.7
													-	8.1	20.3	21.6	24.3	29.7	29.7
														-	12.2	13.5	16.2	21.6	21.6
															-	1.4	4.1	12.2	12.2
																-	2.7	10.8	10.8
																	-	8.1	8.1
																		-	0.0
																			-

V

1	2	3	4	5	6	7	8	9	10	11	12	13	14	15	16	17	18	19	20	21
0.49	0.46	0.45	0.42	0.39	0.39	0.42	0.38	0.38	0.36	0.38	0.39	0.41	0.42	0.45	0.46	0.46	0.42	0.45	0.49	0.49
50.0	50.0	48.6	51.4	54.1	54.1	51.4	55.4	55.4	54.1	52.7	51.4	52.7	54.1	56.8	58.1	55.4	56.8	45.9	41.9	41.9
48.6	48.6	50.0	52.7	55.4	55.4	52.7	56.8	56.8	55.4	54.1	52.7	54.1	55.4	58.1	59.5	56.8	58.1	47.3	43.2	43.2
52.9	52.9	54.3	57.1	60.0	61.4	58.6	62.9	62.9	64.3	62.9	61.4	60.0	61.4	61.4	60.0	57.1	61.4	52.9	48.6	48.6
50.0	52.7	54.1	54.1	56.8	56.8	54.1	58.1	58.1	59.5	58.1	56.8	55.4	54.1	51.4	50.0	50.0	54.1	51.4	50.0	50.0
55.4	58.1	56.8	59.5	59.5	59.5	56.8	60.8	60.8	59.5	58.1	56.8	55.4	54.1	51.4	47.3	44.6	51.4	48.6	47.3	47.3
58.1	60.8	59.5	62.2	59.5	59.5	59.5	63.5	63.5	62.2	60.8	59.5	60.8	56.8	54.1	52.7	47.3	51.4	51.4	52.7	52.7
58.1	60.8	59.5	62.2	59.5	59.5	59.5	63.5	63.5	62.2	60.8	59.5	60.8	56.8	54.1	52.7	47.3	51.4	51.4	52.7	52.7
59.5	62.2	60.8	63.5	60.8	60.8	60.8	64.9	64.9	63.5	62.2	60.8	62.2	58.1	55.4	54.1	48.6	52.7	52.7	54.1	54.1
58.1	60.8	62.2	64.9	62.2	62.2	62.2	66.2	66.2	64.9	63.5	62.2	63.5	59.5	56.8	55.4	50.0	54.1	54.1	55.4	55.4
58.1	60.8	62.2	64.9	62.2	62.2	62.2	66.2	66.2	64.9	63.5	62.2	63.5	59.5	56.8	55.4	50.0	54.1	54.1	55.4	55.4
58.1	60.8	62.2	64.9	64.9	64.9	64.9	68.9	68.9	67.6	66.2	64.9	66.2	62.2	59.5	58.1	52.7	54.1	54.1	55.4	55.4
56.8	59.5	60.8	63.5	63.5	63.5	63.5	67.6	70.3	68.9	67.6	66.2	67.6	63.5	60.8	59.5	54.1	55.4	55.4	56.8	56.8
55.4	58.1	59.5	62.2	59.5	59.5	59.5	63.5	66.2	64.9	63.5	62.2	63.5	59.5	56.8	55.4	50.0	51.4	51.4	52.7	52.7
55.4	58.1	59.5	62.2	59.5	59.5	59.5	63.5	66.2	64.9	63.5	62.2	63.5	59.5	56.8	55.4	50.0	51.4	51.4	52.7	52.7
56.8	59.5	60.8	63.5	60.8	58.1	58.1	62.2	64.9	63.5	62.2	60.8	62.2	58.1	55.4	56.8	51.4	50.0	50.0	51.4	51.4
55.4	58.1	59.5	62.2	59.5	56.8	56.8	60.8	63.5	62.2	60.8	62.2	63.5	59.5	56.8	55.4	50.0	48.6	48.6	50.0	50.0
54.1	56.8	55.4	58.1	55.4	55.4	55.4	59.5	62.2	60.8	59.5	60.8	62.2	58.1	55.4	54.1	48.6	47.3	47.3	48.6	48.6
52.7	55.4	54.1	56.8	54.1	54.1	54.1	58.1	58.1	56.8	52.7	54.1	55.4	51.4	48.6	47.3	44.6	43.2	43.2	47.3	47.3
54.1	56.8	55.4	58.1	55.4	55.4	55.4	56.8	56.8	55.4	51.4	52.7	54.1	50.0	47.3	45.9	43.2	41.9	41.9	45.9	45.9
54.1	54.1	52.7	55.4	52.7	52.7	52.7	54.1	54.1	52.7	48.6	50.0	51.4	50.0	47.3	45.9	45.9	44.6	44.6	48.6	48.6
54.1	51.4	50.0	50.0	52.7	52.7	52.7	54.1	54.1	52.7	51.4	50.0	51.4	55.4	52.7	54.1	54.1	58.1	52.7	54.1	54.1
54.1	51.4	50.0	50.0	52.7	52.7	52.7	54.1	54.1	52.7	51.4	50.0	51.4	55.4	52.7	54.1	54.1	58.1	52.7	54.1	54.1
56.8	54.1	52.7	52.7	55.4	55.4	55.4	56.8	56.8	55.4	54.1	52.7	54.1	58.1	55.4	56.8	56.8	58.1	52.7	56.8	56.8
62.2	59.5	58.1	52.7	55.4	55.4	55.4	54.1	54.1	55.4	54.1	52.7	54.1	58.1	58.1	59.5	59.5	60.8	55.4	56.8	56.8
63.5	60.8	59.5	54.1	56.8	54.1	54.1	52.7	52.7	54.1	52.7	51.4	52.7	56.8	56.8	58.1	60.8	62.2	56.8	58.1	58.1
62.2	59.5	58.1	55.4	58.1	55.4	55.4	54.1	54.1	55.4	54.1	52.7	54.1	58.1	58.1	59.5	59.5	60.8	55.4	56.8	56.8
64.9	62.2	60.8	58.1	60.8	58.1	58.1	56.8	54.1	55.4	54.1	52.7	54.1	58.1	58.1	59.5	59.5	60.8	55.4	56.8	56.8
64.9	62.2	60.8	58.1	60.8	58.1	58.1	56.8	54.1	55.4	54.1	52.7	54.1	58.1	58.1	56.8	56.8	55.4	50.0	51.4	51.4
64.9	62.2	60.8	58.1	60.8	58.1	58.1	56.8	54.1	55.4	54.1	52.7	54.1	58.1	58.1	56.8	56.8	52.7	47.3	48.6	48.6
63.5	63.5	62.2	59.5	62.2	59.5	59.5	58.1	55.4	56.8	52.7	51.4	52.7	56.8	56.8	55.4	55.4	51.4	45.9	47.3	47.3
63.5	63.5	62.2	59.5	62.2	59.5	59.5	58.1	55.4	56.8	52.7	51.4	52.7	56.8	56.8	55.4	55.4	51.4	45.9	47.3	47.3
62.2	62.2	60.8	58.1	60.8	58.1	58.1	56.8	54.1	55.4	51.4	50.0	51.4	55.4	55.4	54.1	54.1	50.0	44.6	45.9	45.9
62.2	62.2	60.8	58.1	60.8	58.1	58.1	56.8	54.1	55.4	51.4	50.0	51.4	55.4	55.4	54.1	54.1	50.0	44.6	45.9	45.9
62.2	62.2	60.8	58.1	60.8	58.1	58.1	56.8	54.1	55.4	51.4	50.0	51.4	55.4	55.4	54.1	54.1	50.0	44.6	45.9	45.9
63.5	60.8	59.5	56.8	59.5	56.8	56.8	55.4	52.7	54.1	52.7	51.4	52.7	56.8	56.8	55.4	55.4	51.4	45.9	47.3	47.3
62.2	59.5	58.1	55.4	58.1	55.4	55.4	54.1	54.1	55.4	54.1	52.7	54.1	58.1	55.4	54.1	54.1	52.7	47.3	48.6	48.6
58.1	55.4	59.5	56.8	59.5	56.8	59.5	58.1	58.1	59.5	58.1	56.8	58.1	59.5	59.5	58.1	55.4	54.1	51.4	52.7	52.7
56.8	54.1	60.8	58.1	60.8	58.1	60.8	62.2	62.2	63.5	62.2	60.8	62.2	63.5	63.5	62.2	59.5	55.4	50.0	51.4	51.4
59.5	56.8	63.5	60.8	63.5	60.8	63.5	62.2	62.2	63.5	62.2	60.8	62.2	63.5	66.2	64.9	67.6	60.8	58.1	59.5	59.5
58.1	55.4	62.2	59.5	62.2	59.5	62.2	60.8	60.8	62.2	60.8	59.5	60.8	62.2	64.9	63.5	66.2	59.5	56.8	58.1	58.1
58.1	60.8	59.5	62.2	59.5	64.9	62.2	58.1	58.1	56.8	58.1	56.8	58.1	56.8	54.1	55.4	52.7	51.4	51.4	52.7	52.7
58.1	60.8	59.5	62.2	59.5	64.9	62.2	58.1	58.1	56.8	58.1	56.8	58.1	56.8	54.1	55.4	52.7	51.4	51.4	52.7	52.7
55.4	58.1	56.8	59.5	56.8	62.2	59.5	55.4	55.4	54.1	55.4	54.1	55.4	54.1	51.4	52.7	50.0	48.6	47.3	55.4	55.4
55.4	58.1	62.2	64.9	62.2	67.6	64.9	63.5	63.5	62.2	63.5	62.2	63.5	62.2	59.5	60.8	58.1	54.1	54.1	55.4	55.4
54.1	56.8	58.1	60.8	60.8	66.2	63.5	62.2	62.2	60.8	59.5	60.8	62.2	60.8	58.1	56.8	54.1	52.7	47.3	48.6	48.6
51.4	54.1	55.4	58.1	58.1	63.5	63.5	62.2	59.5	58.1	56.8	58.1	56.8	55.4	52.7	51.4	48.6	50.0	47.3	48.6	48.6
50.0	52.7	54.1	56.8	56.8	62.2	62.2	60.8	58.1	56.8	55.4	56.8	55.4	54.1	51.4	50.0	47.3	48.6	45.9	47.3	47.3
52.7	55.4	54.1	54.1	54.1	56.8	56.8	55.4	52.7	51.4	50.0	51.4	50.0	48.6	48.6	47.3	44.6	45.9	45.9	47.3	47.3
50.0	52.7	54.1	54.1	54.1	56.8	56.8	55.4	52.7	51.4	50.0	51.4	50.0	48.6	48.6	50.0	47.3	48.6	48.6	50.0	50.0
51.4	54.1	52.7	52.7	52.7	55.4	55.4	54.1	51.4	50.0	48.6	50.0	48.6	47.3	47.3	48.6	45.9	47.3	47.3	48.6	48.6
51.4	54.1	52.7	52.7	52.7	55.4	55.4	54.1	51.4	50.0	48.6	50.0	48.6	47.3	47.3	48.6	45.9	47.3	47.3	48.6	48.6
50.0	52.7	51.4	51.4	51.4	54.1	54.1	52.7	50.0	48.6	47.3	48.6	47.3	45.9	45.9	47.3	47.3	48.6	48.6	50.0	50.0
51.4	54.1	52.7	52.7	52.7	55.4	55.4	54.1	51.4	50.0	48.6	50.0	48.6	47.3	47.3	48.6	45.9	47.3	47.3	48.6	48.6
51.4	54.1	52.7	52.7	52.7	55.4	55.4	54.1	51.4	50.0	48.6	50.0	48.6	47.3	47.3	48.6	45.9	47.3	47.3	48.6	48.6
48.6	51.4	52.7	52.7	52.7	55.4	55.4	54.1	51.4	50.0	48.6	50.0	48.6	47.3	47.3	48.6	45.9	47.3	47.3	48.6	48.6
51.4	54.1	55.4	55.4	55.4	58.1	58.1	56.8	54.1	52.7	51.4	52.7	51.4	50.0	50.0	51.4	48.6	50.0	50.0	51.4	51.4
50.0	52.7	51.4	54.1	56.8	59.5	59.5	58.1	55.4	54.1	52.7	54.1	52.7	48.6	48.6	47.3	47.3	45.9	45.9	47.3	47.3
51.4	51.4	50.0	52.7	55.4	58.1	55.4	54.1	54.1	52.7	56.8	58.1	56.8	55.4	55.4	54.1	54.1	55.4	50.0	51.4	51.4
50.0	50.0	48.6	54.1	56.8	59.5	56.8	55.4	55.4	54.1	58.1	59.5	58.1	56.8	54.1	55.4	52.7	51.4	45.9	47.3	47.3
51.4	51.4	50.0	55.4	58.1	60.8	58.1	56.8	56.8	55.4	56.8	58.1	56.8	55.4	52.7	54.1	51.4	50.0	44.6	45.9	45.9
54.1	56.8	50.0	55.4	55.4	52.7	52.7	51.4	54.1	52.7	54.1	55.4	56.8	52.7	52.7	51.4	48.6	50.0	50.0	51.4	51.4
52.7	55.4	56.8	59.5	56.8	56.8	56.8	55.4	58.1	56.8	58.1	59.5	60.8	56.8	59.5	58.1	55.4	56.8	54.1	55.4	55.4
51.4	54.1	52.7	55.4	55.4	55.4	55.4	54.1	54.1	52.7	54.1	55.4	56.8	55.4	58.1	56.8	54.1	55.4	52.7	54.1	54.1
48.6	51.4	50.0	47.3	44.6	41.9	41.9	40.5	40.5	41.9	43.2	44.6	45.9	44.6	47.3	48.6	48.6	52.7	52.7	51.4	51.4
47.3	50.0	48.6	45.9	45.9	43.2	43.2	41.9	44.6	45.9	47.3	48.6	50.0	48.6	51.4	52.7	52.7	56.8	56.8	55.4	55.4
48.6	51.4	50.0	47.3	47.3	44.6	44.6	43.2	45.9	47.3	48.6	47.3	48.6	47.3	50.0	54.1	54.1	55.4	55.4	56.8	56.8
48.6	51.4	50.0	47.3	47.3	44.6	44.6	43.2	45.9	47.3	48.6	47.3	48.6	47.3	50.0	54.1	54.1	55.4	55.4	56.8	56.8
48.6	51.4	50.0	47.3	47.3	44.6	44.6	43.2	43.2	44.6	45.9	44.6	45.9	44.6	47.3	51.4	51.4	52.7	55.4	56.8	56.8
48.6	51.4	50.0	47.3	47.3	44.6	44.6	43.2	43.2	44.6	45.9	44.6	45.9	44.6	47.3	51.4	51.4	52.7	55.4	56.8	56.8
48.6	48.6	50.0	47.3	47.3	44.6	44.6	43.2	43.2	44.6	48.6	47.3	48.6	47.3	50.0	54.1	54.1	55.4	58.1	59.5	59.5
50.0	50.0	48.6	45.9	45.9	43.2	43.2	41.9	41.9	43.2	47.3	45.9	47.3	45.9	48.6	52.7	52.7	54.1	56.8	58.1	58.1
50.0	50.0	48.6	45.9	45.9	43.2	43.2	41.9	41.9	43.2	47.3	45.9	47.3	45.9	48.6	52.7	52.7	54.1	56.8	58.1	58.1
50.0	50.0	48.6	45.9	45.9	43.2	43.2	41.9	41.9	43.2	47.3	45.9	47.3	45.9	48.6	52.7	52.7	54.1	56.8	58.1	58.1
50.0	50.0	48.6	45.9	45.9	43.2	43.2	41.9	41.9	43.2	47.3	45.9	47.3	45.9	48.6	52.7	52.7	54.1	56.8	58.1	58.1
50.0	50.0	48.6	45.9	45.9	43.2	45.9	44.6	41.9	43.2	47.3	45.9	47.3	45.9	48.6	52.7	52.7	51.4	56.8	58.1	58.1
48.6	51.4	50.0	47.3	47.3	47.3	50.0	51.4	48.6	50.0	51.4	50.0	51.4	50.0	52.7	56.8	56.8	55.4	60.8	62.2	62.2
47.3	50.0	48.6	45.9	45.9	45.9	48.6	50.0	47.3	48.6	50.0	48.6	50.0	48.6	51.4	55.4	55.4	54.1	59.5	60.8	60.8
44.6	47.3	48.6	48.6	48.6	48.6	51.4	52.7	50.0	48.6	50.0	48.6	50.0	48.6	51.4	55.4	55.4	54.1	59.5	60.8	60.8
47.3	50.0	48.6	45.9	45.9	43.2	45.9	47.3	44.6	43.2	44.6	43.2	44.6	43.2	45.9	52.7	55.4	51.4	56.8	60.8	60.8
47.3	50.0	48.6	45.9	45.9	43.2	45.9	47.3	44.6	43.2	44.6	43.2	44.6	43.2	45.9	52.7	55.4	51.4	56.8	60.8	60.8
-	2.7	12.2	17.6	17.6	23.0	25.7	29.7	29.7	28.4	29.7	31.1	32.4	33.8	33.8	37.8	40.5	47.3	52.7	51.4	51.4
	-	9.5	14.9	14.9	20.3	23.0	27.0	27.0	25.7	29.7	31.1	32.4	36.5	36.5	40.5	43.2	50.0	55.4	54.1	54.1
		-	5.4	8.1	13.5	16.2	20.3	20.3	18.9	23.0	24.3	25.7	29.7	29.7	31.1	33.8	40.5	45.9	44.6	44.6
			-	2.7	8.1	10.8	14.9	14.9	16.2	20.3	21.6	23.0	27.0	29.7	31.1	36.5	43.2	48.6	50.0	50.0
				-	5.4	8.1	12.2	12.2	13.5	17.6	18.9	20.3	24.3	27.0	31.1	36.5	43.2	48.6	50.0	50.0
					-	2.7	6.8	6.8	8.1	12.2	13.5	14.9	18.9	21.6	25.7	31.1	37.8	43.2	44.6	44.6
						-	4.1	6.8	8.1	12.2	13.5	14.9	18.9	21.6	25.7	31.1	37.8	40.5	41.9	41.9
							-	2.7	4.1	8.1	9.5	10.8	14.9	17.6	21.6	27.0	33.8	39.2	40.5	40.5
								-	4.1	8.1	9.5	10.8	14.9	17.6	21.6	27.0	33.8	39.2	40.5	40.5
									-	4.1	5.4	6.8	10.8	13.5	17.6	23.0	29.7	37.8	39.2	39.2
										-	1.4	2.7	6.8	9.5	13.5	18.9	25.7	33.8	35.1	35.1
											-	1.4	5.4	8.1	14.9	20.3	27.0	35.1	36.5	36.5
												-	4.1	6.8	13.5	18.9	25.7	33.8	35.1	35.1
													-	2.7	9.5	14.9	21.6	32.4	33.8	33.8
														-	6.8	12.2	18.9	29.7	31.1	31.1
															-	5.4	14.9	25.7	27.0	27.0
																-	9.5	20.3	21.6	21.6
																	-	10.8	14.9	14.9
																		-	4.1	4.1
																			-	0.0
																				-

X

1	2	3	4	5	6	7	8	9	10	11	12	13	14	15	16	17	18	19	20
0.62	0.64	0.62	0.65	0.65	0.66	0.68	0.61	0.65	0.62	0.59	0.61	0.55	0.54	0.57	0.51	0.58	0.54	0.53	0.53
50.0	48.6	47.3	44.6	44.6	43.2	41.9	43.2	39.2	41.9	39.2	40.5	45.9	44.6	50.0	52.7	48.6	50.0	48.6	48.6
51.4	50.0	48.6	45.9	45.9	44.6	43.2	44.6	40.5	43.2	40.5	41.9	47.3	45.9	51.4	54.1	50.0	51.4	50.0	50.0
47.1	45.7	47.1	44.3	44.3	42.9	41.4	45.7	41.4	44.3	41.4	42.9	45.7	45.7	44.3	45.7	41.4	42.9	41.4	41.4
39.2	37.8	39.2	39.2	39.2	37.8	36.5	43.2	39.2	41.9	41.9	40.5	45.9	44.6	44.6	50.0	48.6	52.7	51.4	51.4
44.6	43.2	44.6	44.6	44.6	43.2	41.9	45.9	41.9	47.3	44.6	43.2	45.9	44.6	44.6	47.3	45.9	50.0	48.6	51.4
50.0	48.6	50.0	50.0	47.3	45.9	44.6	45.9	41.9	47.3	44.6	43.2	43.2	39.2	41.9	41.9	40.5	47.3	45.9	48.6
50.0	48.6	50.0	50.0	47.3	45.9	44.6	45.9	41.9	47.3	44.6	43.2	43.2	39.2	41.9	41.9	40.5	47.3	45.9	48.6
48.6	47.3	48.6	48.6	45.9	44.6	43.2	44.6	40.5	45.9	43.2	41.9	41.9	37.8	40.5	40.5	39.2	45.9	44.6	47.3
47.3	45.9	47.3	47.3	44.6	43.2	41.9	43.2	39.2	44.6	41.9	40.5	40.5	36.5	39.2	39.2	37.8	47.3	45.9	48.6
47.3	45.9	47.3	47.3	44.6	43.2	41.9	43.2	39.2	44.6	41.9	40.5	40.5	36.5	39.2	39.2	37.8	47.3	45.9	48.6
47.3	45.9	47.3	47.3	44.6	43.2	41.9	43.2	39.2	44.6	41.9	40.5	40.5	36.5	41.9	41.9	40.5	50.0	48.6	51.4
48.6	47.3	48.6	48.6	45.9	44.6	43.2	44.6	40.5	43.2	43.2	41.9	41.9	37.8	43.2	45.9	41.9	51.4	52.7	55.4
44.6	43.2	44.6	44.6	41.9	40.5	39.2	40.5	41.9	44.6	44.6	43.2	43.2	39.2	44.6	47.3	43.2	52.7	54.1	56.8
44.6	43.2	44.6	44.6	41.9	40.5	39.2	40.5	41.9	44.6	44.6	43.2	43.2	39.2	44.6	47.3	43.2	52.7	54.1	56.8
40.5	39.2	40.5	40.5	37.8	36.5	35.1	39.2	40.5	43.2	43.2	44.6	44.6	40.5	45.9	51.4	47.3	54.1	58.1	58.1
41.9	40.5	41.9	41.9	39.2	37.8	36.5	40.5	41.9	44.6	44.6	45.9	45.9	41.9	47.3	52.7	48.6	55.4	59.5	59.5
43.2	41.9	43.2	43.2	40.5	39.2	37.8	41.9	43.2	45.9	45.9	47.3	47.3	43.2	48.6	51.4	47.3	51.4	55.4	55.4
44.6	43.2	44.6	44.6	41.9	40.5	39.2	43.2	44.6	44.6	44.6	45.9	45.9	41.9	44.6	47.3	45.9	50.0	54.1	54.1
45.9	44.6	45.9	45.9	43.2	41.9	40.5	41.9	43.2	43.2	43.2	44.6	44.6	40.5	43.2	45.9	44.6	48.6	52.7	52.7
45.9	44.6	45.9	45.9	43.2	41.9	40.5	44.6	45.9	45.9	45.9	44.6	44.6	40.5	43.2	48.6	47.3	48.6	52.7	52.7
56.8	58.1	54.1	54.1	56.8	58.1	59.5	55.4	54.1	54.1	54.1	52.7	50.0	48.6	45.9	45.9	52.7	56.8	52.7	50.0
56.8	58.1	54.1	54.1	56.8	58.1	59.5	55.4	54.1	54.1	54.1	52.7	50.0	48.6	45.9	45.9	52.7	56.8	52.7	50.0
56.8	58.1	54.1	54.1	56.8	58.1	59.5	55.4	54.1	54.1	54.1	52.7	50.0	48.6	45.9	45.9	55.4	59.5	55.4	52.7
54.1	55.4	51.4	51.4	54.1	55.4	56.8	50.0	51.4	54.1	54.1	55.4	58.1	56.8	54.1	51.4	60.8	64.9	60.8	58.1
55.4	56.8	52.7	52.7	55.4	56.8	58.1	51.4	55.4	58.1	55.4	59.5	59.5	58.1	58.1	55.4	64.9	66.2	64.9	62.2
59.5	60.8	56.8	56.8	59.5	60.8	62.2	55.4	59.5	59.5	56.8	60.8	60.8	59.5	59.5	59.5	66.2	64.9	63.5	63.5
59.5	60.8	56.8	54.1	56.8	58.1	59.5	52.7	56.8	56.8	54.1	58.1	58.1	56.8	56.8	56.8	63.5	62.2	60.8	60.8
56.8	58.1	54.1	54.1	56.8	58.1	59.5	55.4	59.5	59.5	56.8	60.8	60.8	56.8	62.2	62.2	68.9	67.6	66.2	66.2
56.8	58.1	54.1	54.1	56.8	58.1	59.5	58.1	62.2	59.5	56.8	60.8	63.5	59.5	64.9	64.9	71.6	67.6	66.2	66.2
58.1	59.5	55.4	55.4	55.4	56.8	58.1	59.5	60.8	58.1	55.4	59.5	62.2	58.1	63.5	63.5	70.3	66.2	64.9	64.9
58.1	59.5	55.4	55.4	55.4	56.8	58.1	59.5	60.8	58.1	55.4	59.5	62.2	58.1	63.5	63.5	70.3	66.2	64.9	64.9
56.8	58.1	54.1	54.1	54.1	55.4	56.8	58.1	59.5	56.8	54.1	58.1	63.5	59.5	62.2	62.2	68.9	67.6	66.2	66.2
56.8	58.1	54.1	54.1	54.1	55.4	56.8	58.1	59.5	56.8	54.1	58.1	63.5	59.5	62.2	62.2	68.9	67.6	66.2	66.2
56.8	58.1	54.1	54.1	54.1	55.4	56.8	58.1	59.5	56.8	54.1	58.1	63.5	59.5	62.2	62.2	68.9	67.6	66.2	66.2
55.4	56.8	52.7	52.7	55.4	56.8	58.1	56.8	60.8	58.1	55.4	59.5	64.9	60.8	63.5	63.5	70.3	68.9	67.6	67.6
56.8	58.1	54.1	54.1	56.8	58.1	59.5	60.8	64.9	62.2	59.5	63.5	68.9	64.9	64.9	64.9	71.6	70.3	68.9	68.9
50.0	48.6	50.0	52.7	55.4	54.1	55.4	56.8	60.8	58.1	55.4	59.5	59.5	55.4	58.1	58.1	64.9	66.2	64.9	64.9
48.6	47.3	48.6	51.4	54.1	52.7	54.1	58.1	62.2	62.2	59.5	63.5	66.2	62.2	64.9	62.2	68.9	67.6	68.9	68.9
45.9	44.6	48.6	48.6	51.4	50.0	48.6	55.4	54.1	56.8	56.8	58.1	58.1	56.8	59.5	62.2	63.5	62.2	66.2	66.2
44.6	43.2	47.3	47.3	50.0	48.6	47.3	54.1	52.7	55.4	55.4	56.8	59.5	58.1	58.1	60.8	62.2	63.5	67.6	67.6
66.2	67.6	60.8	58.1	55.4	56.8	58.1	56.8	52.7	47.3	44.6	43.2	43.2	41.9	47.3	50.0	43.2	39.2	35.1	32.4
66.2	67.6	60.8	58.1	55.4	56.8	58.1	56.8	52.7	47.3	44.6	43.2	43.2	41.9	47.3	50.0	43.2	39.2	35.1	32.4
60.8	62.2	55.4	52.7	50.0	51.4	52.7	51.4	47.3	41.9	39.2	37.8	40.5	39.2	44.6	47.3	43.2	41.9	37.8	35.1
58.1	56.8	52.7	50.0	47.3	45.9	47.3	48.6	44.6	39.2	39.2	37.8	43.2	44.6	47.3	50.0	43.2	36.5	37.8	35.1
64.9	63.5	62.2	59.5	56.8	55.4	56.8	55.4	51.4	48.6	45.9	44.6	44.6	45.9	48.6	51.4	44.6	43.2	44.6	41.9
64.9	63.5	62.2	59.5	56.8	55.4	56.8	55.4	51.4	48.6	45.9	44.6	44.6	48.6	48.6	48.6	41.9	43.2	44.6	41.9
66.2	64.9	60.8	58.1	55.4	54.1	55.4	54.1	50.0	47.3	44.6	43.2	43.2	47.3	47.3	47.3	40.5	41.9	43.2	40.5
60.8	59.5	55.4	52.7	50.0	48.6	50.0	48.6	44.6	41.9	39.2	40.5	43.2	47.3	47.3	47.3	40.5	41.9	43.2	40.5
63.5	62.2	58.1	55.4	52.7	51.4	52.7	48.6	44.6	41.9	39.2	40.5	43.2	47.3	47.3	47.3	40.5	41.9	43.2	40.5
62.2	60.8	56.8	54.1	51.4	50.0	51.4	47.3	43.2	40.5	37.8	39.2	41.9	45.9	45.9	45.9	39.2	40.5	41.9	39.2
62.2	60.8	56.8	54.1	51.4	50.0	51.4	47.3	43.2	40.5	37.8	39.2	41.9	45.9	45.9	45.9	39.2	40.5	41.9	39.2
60.8	59.5	55.4	52.7	50.0	48.6	50.0	48.6	44.6	41.9	39.2	37.8	40.5	44.6	44.6	47.3	40.5	41.9	43.2	40.5
62.2	60.8	56.8	54.1	51.4	50.0	51.4	47.3	43.2	40.5	37.8	39.2	41.9	45.9	45.9	45.9	39.2	40.5	41.9	39.2
62.2	60.8	56.8	54.1	51.4	50.0	51.4	47.3	43.2	40.5	37.8	39.2	41.9	45.9	45.9	45.9	39.2	40.5	41.9	39.2
62.2	60.8	56.8	56.8	54.1	52.7	54.1	50.0	45.9	43.2	40.5	41.9	44.6	48.6	48.6	48.6	41.9	43.2	44.6	41.9
62.2	60.8	56.8	56.8	54.1	52.7	54.1	50.0	45.9	43.2	40.5	41.9	44.6	48.6	48.6	48.6	41.9	43.2	44.6	41.9
52.7	51.4	50.0	50.0	50.0	48.6	50.0	51.4	47.3	44.6	44.6	43.2	48.6	52.7	52.7	55.4	51.4	52.7	54.1	54.1
51.4	50.0	48.6	48.6	51.4	50.0	51.4	50.0	48.6	45.9	45.9	47.3	52.7	54.1	51.4	56.8	50.0	48.6	50.0	50.0
55.4	54.1	52.7	52.7	55.4	54.1	55.4	51.4	50.0	47.3	47.3	51.4	51.4	52.7	50.0	52.7	45.9	44.6	45.9	45.9
54.1	52.7	54.1	54.1	56.8	55.4	56.8	52.7	51.4	48.6	48.6	52.7	52.7	54.1	51.4	54.1	47.3	45.9	47.3	47.3
51.4	52.7	51.4	54.1	54.1	55.4	54.1	55.4	51.4	51.4	56.8	58.1	52.7	54.1	51.4	56.8	50.0	48.6	52.7	52.7
50.0	51.4	47.3	50.0	47.3	48.6	50.0	48.6	44.6	41.9	47.3	48.6	51.4	52.7	52.7	55.4	48.6	55.4	56.8	59.5
54.1	55.4	54.1	56.8	54.1	55.4	56.8	52.7	48.6	48.6	51.4	52.7	52.7	54.1	54.1	54.1	47.3	51.4	52.7	55.4
48.6	50.0	48.6	51.4	48.6	50.0	48.6	41.9	43.2	45.9	51.4	52.7	52.7	54.1	54.1	54.1	47.3	54.1	55.4	55.4
55.4	56.8	58.1	60.8	58.1	59.5	58.1	51.4	52.7	55.4	52.7	54.1	54.1	52.7	52.7	52.7	48.6	52.7	51.4	51.4
56.8	58.1	59.5	62.2	59.5	60.8	59.5	52.7	54.1	56.8	54.1	55.4	55.4	54.1	54.1	54.1	50.0	51.4	50.0	50.0
56.8	58.1	59.5	62.2	59.5	60.8	59.5	52.7	54.1	56.8	54.1	55.4	55.4	54.1	54.1	54.1	50.0	51.4	50.0	50.0
54.1	55.4	56.8	59.5	56.8	58.1	56.8	50.0	51.4	54.1	51.4	52.7	55.4	54.1	54.1	54.1	50.0	51.4	50.0	50.0
54.1	55.4	56.8	59.5	56.8	58.1	56.8	50.0	51.4	54.1	51.4	52.7	55.4	54.1	54.1	54.1	50.0	51.4	50.0	50.0
51.4	52.7	54.1	56.8	56.8	58.1	56.8	47.3	51.4	54.1	51.4	52.7	55.4	54.1	54.1	54.1	50.0	54.1	52.7	52.7
52.7	54.1	55.4	58.1	58.1	59.5	58.1	48.6	52.7	55.4	52.7	54.1	56.8	55.4	55.4	55.4	51.4	52.7	51.4	51.4
52.7	54.1	55.4	58.1	58.1	59.5	58.1	48.6	52.7	55.4	52.7	54.1	56.8	55.4	55.4	55.4	51.4	52.7	51.4	51.4
52.7	54.1	55.4	58.1	58.1	59.5	58.1	48.6	52.7	55.4	52.7	54.1	56.8	55.4	55.4	55.4	51.4	52.7	51.4	51.4
52.7	54.1	55.4	58.1	58.1	59.5	58.1	48.6	52.7	55.4	52.7	54.1	56.8	55.4	55.4	55.4	51.4	52.7	51.4	51.4
60.8	62.2	60.8	63.5	63.5	64.9	63.5	54.1	58.1	60.8	58.1	56.8	59.5	58.1	58.1	52.7	54.1	55.4	54.1	54.1
62.2	63.5	59.5	59.5	56.8	58.1	56.8	52.7	51.4	54.1	51.4	50.0	52.7	54.1	54.1	45.9	50.0	54.1	52.7	52.7
60.8	62.2	58.1	58.1	55.4	56.8	55.4	51.4	50.0	52.7	50.0	48.6	51.4	52.7	52.7	44.6	48.6	55.4	54.1	54.1
58.1	59.5	55.4	55.4	52.7	54.1	52.7	48.6	47.3	50.0	47.3	45.9	48.6	50.0	50.0	41.9	45.9	52.7	51.4	51.4
52.7	54.1	50.0	50.0	47.3	48.6	47.3	43.2	41.9	44.6	41.9	40.5	43.2	44.6	47.3	44.6	51.4	55.4	54.1	51.4
52.7	54.1	50.0	50.0	47.3	48.6	47.3	43.2	41.9	44.6	41.9	40.5	43.2	44.6	47.3	44.6	51.4	55.4	54.1	51.4
48.6	47.3	48.6	51.4	48.6	47.3	48.6	47.3	48.6	48.6	48.6	47.3	44.6	43.2	43.2	43.2	36.5	43.2	41.9	41.9
48.6	47.3	48.6	51.4	51.4	50.0	51.4	47.3	51.4	51.4	51.4	50.0	47.3	45.9	45.9	45.9	39.2	43.2	41.9	41.9
50.0	51.4	50.0	52.7	55.4	56.8	58.1	54.1	58.1	58.1	58.1	56.8	54.1	52.7	52.7	52.7	45.9	41.9	40.5	40.5
47.3	48.6	47.3	50.0	52.7	54.1	55.4	51.4	55.4	55.4	55.4	54.1	54.1	52.7	52.7	52.7	48.6	47.3	45.9	43.2
47.3	48.6	47.3	50.0	50.0	51.4	52.7	48.6	52.7	52.7	52.7	51.4	51.4	50.0	50.0	50.0	45.9	44.6	43.2	40.5
47.3	48.6	47.3	50.0	50.0	51.4	50.0	45.9	50.0	52.7	52.7	54.1	54.1	52.7	52.7	52.7	51.4	50.0	48.6	45.9
47.3	48.6	47.3	47.3	47.3	48.6	47.3	43.2	47.3	50.0	50.0	51.4	54.1	52.7	52.7	55.4	51.4	47.3	45.9	43.2
51.4	52.7	51.4	51.4	51.4	52.7	51.4	44.6	48.6	51.4	51.4	52.7	52.7	51.4	51.4	54.1	50.0	45.9	44.6	41.9
51.4	52.7	51.4	51.4	51.4	52.7	51.4	44.6	48.6	51.4	51.4	52.7	52.7	51.4	51.4	51.4	50.0	45.9	44.6	41.9
50.0	51.4	50.0	50.0	50.0	51.4	50.0	43.2	47.3	50.0	50.0	51.4	51.4	50.0	50.0	50.0	48.6	44.6	43.2	40.5
51.4	52.7	54.1	54.1	51.4	52.7	51.4	47.3	48.6	51.4	51.4	52.7	52.7	51.4	48.6	48.6	50.0	45.9	44.6	41.9
50.0	51.4	52.7	52.7	50.0	51.4	50.0	45.9	47.3	50.0	50.0	51.4	51.4	50.0	47.3	47.3	48.6	44.6	43.2	40.5
48.6	50.0	51.4	51.4	48.6	50.0	48.6	44.6	45.9	48.6	48.6	50.0	50.0	51.4	45.9	45.9	47.3	43.2	41.9	39.2
47.3	48.6	50.0	50.0	47.3	48.6	47.3	45.9	47.3	50.0	50.0	48.6	48.6	50.0	44.6	44.6	45.9	44.6	43.2	40.5
50.0	51.4	50.0	50.0	47.3	48.6	47.3	45.9	47.3	50.0	50.0	48.6	45.9	47.3	41.9	41.9	43.2	41.9	40.5	37.8
48.6	50.0	48.6	48.6	48.6	50.0	48.6	50.0	51.4	54.1	54.1	52.7	52.7	54.1	48.6	45.9	47.3	45.9	44.6	44.6
51.4	52.7	51.4	51.4	51.4	52.7	51.4	50.0	51.4	54.1	54.1	55.4	55.4	56.8	51.4	45.9	47.3	43.2	41.9	44.6
52.7	54.1	50.0	50.0	50.0	51.4	50.0	54.1	55.4	55.4	55.4	56.8	56.8	58.1	58.1	52.7	54.1	47.3	48.6	51.4
55.4	56.8	50.0	50.0	50.0	51.4	50.0	54.1	55.4	55.4	55.4	59.5	59.5	60.8	60.8	58.1	56.8	50.0	51.4	54.1
54.1	55.4	48.6	48.6	48.6	50.0	48.6	52.7	54.1	56.8	56.8	60.8	60.8	62.2	62.2	59.5	55.4	48.6	50.0	52.7
54.1	55.4	48.6	48.6	48.6	50.0	48.6	52.7	54.1	56.8	56.8	60.8	60.8	62.2	62.2	59.5	55.4	48.6	50.0	52.7
-	1.4	8.1	10.8	13.5	14.9	16.2	23.0	27.0	27.0	32.4	33.8	41.9	43.2	40.5	45.9	44.6	56.8	60.8	58.1
	-	9.5	12.2	14.9	13.5	14.9	21.6	25.7	25.7	31.1	32.4	40.5	41.9	39.2	44.6	43.2	55.4	59.5	56.8
		-	2.7	5.4	6.8	8.1	14.9	18.9	18.9	24.3	25.7	33.8	37.8	37.8	43.2	41.9	54.1	58.1	55.4
			-	2.7	4.1	5.4	12.2	16.2	16.2	21.6	23.0	33.8	37.8	37.8	43.2	41.9	51.4	55.4	52.7
				-	1.4	2.7	12.2	13.5	13.5	18.9	20.3	31.1	35.1	35.1	40.5	39.2	48.6	52.7	50.0
					-	1.4	10.8	12.2	12.2	17.6	18.9	29.7	33.8	33.8	39.2	37.8	47.3	51.4	48.6
						-	9.5	10.8	13.5	18.9	20.3	31.1	35.1	35.1	40.5	39.2	48.6	52.7	50.0
							-	4.1	9.5	14.9	18.9	27.0	31.1	31.1	33.8	32.4	44.6	45.9	43.2
								-	5.4	10.8	14.9	23.0	27.0	27.0	29.7	28.4	40.5	41.9	39.2
									-	5.4	9.5	20.3	24.3	24.3	29.7	28.4	40.5	41.9	39.2
										-	4.1	14.9	18.9	18.9	24.3	25.7	37.8	36.5	33.8
											-	10.8	14.9	14.9	23.0	24.3	36.5	35.1	32.4
												-	4.1	6.8	14.9	16.2	28.4	27.0	24.3
													-	8.1	16.2	17.6	29.7	28.4	25.7
														-	8.1	12.2	27.0	25.7	23.0
															-	9.5	24.3	23.0	23.0
																-	14.9	16.2	16.2
																	-	4.1	6.8
																		-	2.7
																			-
