# Supplementary material for: Mapping Determinants of Gene Expression Plasticity by Genetical Genomics in C. elegans
Source: PLoS Genet. 2006 Dec 29;2(12):e222. doi: 10.1371/journal.pgen.0020222 (PMC1756913; doi:10.1371/journal.pgen.0020222)
Supplement: Table S4 — (17 KB PDF) [file pgen.0020222.st004.pdf]

		gene WITH annotation		gene WITHOUT annotation
genes WITH cis_QTL at transband	Num=18	F36D3.2	Num=25	C47E8.8
		F09C6.9		W06A7.5
		C29F3.6		T09F5.11
		T06E6.3		C29F3.7
		W08G11.4		T26H5.4
		F08E10.3		K06B4.3
		F11A5.12		Y6E2A.8
		F21H7.4		T06C12.10
		K06B4.4		F35E8.11
		F26D2.10		F44G3.10
		T10H4.11		F21H7.1
		Y102A5B.3		T13F3.6
		T09F5.9		F57E7.1
		Y102A5C.17		Y102A5C.1
		Y102A5C.16		Y102A5C.3
		T27C5.7		ZK218.4
		F57A10.2		ZK218.8
		R12G8.2		T19C9.8
				F47H4.9
				F47H4.10
				F47H4.2
				Y20C6A.1
				C38D9.9
				Y6G8.2
				F59A1.8
gene with cis-Interaction effect at transband		/		Y75B12B.3
gene with both cis-QTL and cis-Interaction		/		T09F5.10
gene without statistically significant QTL or Interaction effect at transband	Num=451	Y49A3A.5	Num=323	Y49A3A.3
		F56A12.1		Y49A3A.4
		F56A12.2		F23B12.9
		C15H11.1		F23B12.3
		C15H11.2		F23B12.4
		C15H11.3		F08H9.2
		C15H11.6		F08H9.3
		C15H11.5		F08H9.4
		C15H11.4		R02D5.7
		C15H11.7		R02D5.3
		C15H11.8		R02D5.4
		C15H11.9		R02D5.1
		F23B12.1		R02D5.6
		F23B12.5		C53A5.13
		F23B12.6		C53A5.8
		F23B12.7		C53A5.10
		F23B12.8		R11H6.4
		F08H9.1		F43D2.4

F08H9.6	F43D2.3
F08H9.5	F43D2.2
F08H9.7	C54G10.1
F08H9.8	C47E8.1
C53A5.2	C47E8.6
C53A5.1	R08A2.5
C53A5.3	R08A2.4
C53A5.4	R08A2.1
C53A5.5	Y50E8A.1
C53A5.6	Y50E8A.3
C53A5.9	Y50E8A.8
C53A5.11	Y50E8A.15
Y40H4A.2	Y50E8A.14
R11H6.1	Y50E8A.9
R11H6.2	Y50E8A.10
R11H6.3	Y50E8A.11
R11H6.5	Y50E8A.12
F43D2.1	C48G7.1
C54G10.2	W06A7.2
C54G10.3	W06A7.4
C54G10.4a	ZC412.4
C54G10.4b	ZC412.3
C47E8.3	ZC412.5
C47E8.5	ZC412.6
C47E8.4	ZC412.9
C47E8.7	H12D21.2
R08A2.3	H12D21.5
R08A2.2	H12D21.6
Y50E8A.2	H12D21.10
Y50E8A.4a	H12D21.9
Y50E8A.4b	W09D12.2
Y50E8A.5	F02D8.1
Y50E8A.6	F02D8.2
Y50E8A.7	F02D8.3
Y50E8A.16	T01C3.1
C48G7.3	T01C3.2
C48G7.2	T01C3.5
W06A7.3a	T01C3.8
W06A7.3b	T01C3.9
W06A7.3c	F14H8.2
ZC412.1	F14H8.5
ZC412.2	F14H8.4
H12D21.4	C25D7.4
H12D21.7	C25D7.5
H12D21.8	C25D7.9
W09D12.1	C25D7.8
C30G7.1	C25D7.10
F02D8.4	C01G10.11a
T01C3.3	C01G10.11b
T01C3.4	C01G10.8
T01C3.6	C01G10.6
T01C3.7	C01G10.5

T01C3.10	C01G10.4
F14H8.1	C01G10.3
F14H8.6	C01G10.2
C25D7.1	C01G10.13
C25D7.2	T01G5.4
C25D7.3	K08F9.2
C25D7.6	K08F9.4
C25D7.7	T09F5.2
C01G10.12	Y75B12B.1
C01G10.10	M01B2.4
C01G10.9	M01B2.3
C01G10.7	M01B2.8
C01G10.1	M01B2.10
C01G10.14	T10H4.4
T01G5.7	T26H8.3
T01G5.6	ZK1037.3
T01G5.1	ZK1037.6
T01G5.2	C29F3.3
T01G5.3	R08H2.8
K08F9.1	T06E6.11
K08F9.3	T06E6.10
T09F5.1	T06E6.13
T09F5.5	Y36E3A.1
T09F5.7	T23F1.5
T09F5.3	T23F1.6
T09F5.8	C06B8.2a
Y75B12B.2	C06B8.2b
Y75B12B.4	F21A3.3
Y75B12B.5	F21A3.4
Y75B12B.6	F28F8.4
Y75B12B.7	F28F8.5
C41G6.11	F28F8.7
C41G6.14	B0391.10
C41G6.10	B0391.9
C41G6.9	B0391.5
C41G6.6	C55A1.9
C41G6.7	C55A1.6
C41G6.8	C55A1.8
C41G6.1	F53E4.1
C41G6.3	K06B4.9
C41G6.15	Y6E2A.4
C41G6.5	Y6E2A.5
M01B2.6	Y6E2A.7
M01B2.1	T23D5.3
M01B2.9	F57A10.4
M01B2.7	T26E4.2
T10H4.3	T26E4.5
T10H4.5	T26E4.4
T10H4.6	T26E4.7
T10H4.8	T26E4.10
T10H4.9	T26E4.9
T10H4.10	F54B8.1

T10H4.2	F54B8.3
T10H4.12	F54B8.4
T26H8.2	F54B8.5
ZK1037.1	F54B8.8
ZK1037.4	F54B8.9
ZK1037.5	F54B8.10
ZK1037.7	F54B8.12
ZK1037.8	F54B8.11
ZK1037.9	K08G2.7
ZK1037.10	T06C12.4
C29F3.2	T06C12.12
C29F3.5	T06C12.14
C29F3.4	F35E8.1
C29F3.1	F35E8.2
R08H2.9	F35E8.6
R08H2.10	F35E8.7
R08H2.7	F35E8.8
R08H2.5	F35E8.9
R08H2.4	F35E8.10
R08H2.1	T05G11.2
R08H2.2	F36G9.3
R08H2.13	F36G9.7
R08H2.3	F36G9.6
T06E6.1	F36G9.5
T06E6.2b	F36G9.14
T06E6.4	F36G9.13
T06E6.5	F36G9.15
T06E6.7	C06C6.6
T06E6.6	C06C6.7
T06E6.8	C06C6.9
T06E6.9	C06C6.8
T26H5.3	T10C6.4
T23F1.3	T10C6.5
T23F1.4	T10C6.7
T23F1.7a	T10C6.9
T23F1.7b	T10C6.10
C06B8.1	F14H3.3
C06B8.3	F14H3.5
C06B8.4	F14H3.6
C06B8.6	F14H3.9
C06B8.7	F22B8.4
C06B8.8	F22B8.7
F21A3.1	F44G3.6
F21A3.2	F44G3.8
F21A3.5	F10A3.1
F21A3.6	F10A3.2
F28F8.1	F10A3.3
F28F8.2	F10A3.4
F28F8.3	F10A3.11
F28F8.6	K05D4.3
B0391.11	F11A5.6
B0391.4	F11A5.13

B0391.3	F21H7.2
C55A1.1	F21H7.12
C55A1.3	F21H7.3
C55A1.5	T13F3.4
K06B4.1	T13F3.5
K06B4.2	F28G4.2
K06B4.5	F28G4.4
K06B4.7	F28G4.3
K06B4.6	C31A11.3
K06B4.8	C31A11.4
K06B4.10	F57G8.7
K06B4.11	W08G11.1
K06B4.12	W08G11.3
Y6E2A.1	F13A7.7
Y6E2A.2	F13A7.9
Y6E2A.6	F13A7.11
T23D5.2	F26D2.13
T23D5.1	F26D2.14
T23D5.6	T08G3.6
T23D5.7	T08G3.8
T23D5.8	T08G3.11
T23D5.10	F57E7.2
T23D5.9	Y32B12A.4
T23D5.11	F36D3.1
F57A10.1	F36D3.3
F57A10.3	F36D3.8
F57A10.5	Y32B12B.1
T26E4.1	Y32B12B.2
T26E4.3	Y32B12B.3
T26E4.8	Y32B12B.4
T26E4.11	T03E6.8
T26E4.12	Y32B12C.3
T26E4.14	Y70C5A.2
T26E4.15	W06G6.2
F54B8.2	W06G6.13
F54B8.6	W06G6.6
K08G2.5	W06G6.7
T06C12.2	W06G6.8
T06C12.3	W06G6.9
T06C12.6	W06G6.10
T06C12.7	W06G6.11
T06C12.8	W06G6.12
T06C12.9	Y70C5B.1
T06C12.11	F14F8.8
T06C12.13	F14F8.3
T06C12.1	F14F8.4
F35E8.12	Y70C5C.5
T05G11.3	T25E12.11
T05G11.4	T25E12.6
T05G11.6	T25E12.4b
H24D24.2	W06H3.1
H24D24.1	T20B3.14

F36G9.1	T20B3.2
F36G9.2	T20B3.8
F36G9.9	F49A5.6
F36G9.8	F09C6.1
F36G9.11	F09C6.2
F36G9.12	F09C6.6
C06C6.5a	F09C6.10
C06C6.5b	Y102A5C.2
C06C6.4	Y102A5C.4
C06C6.3	Y102A5C.9
C06C6.2	Y102A5C.10
T10C6.2	Y102A5C.12
T10C6.1	Y102A5C.13
T10C6.3	Y102A5C.19
T10C6.6a	Y102A5C.23
T10C6.6b	Y102A5C.25
T10C6.11	Y102A5C.27
T10C6.13	Y102A5C.33
ZK285.1	F49H6.3
F14H3.2	F49H6.12
F14H3.4	F49H6.8
F14H3.7	F49H6.11
F14H3.8	F28B1.1
F14H3.10	F28B1.2
F14H3.11	F28B1.3
F14H3.1	F28B1.4
F14H3.12	F28B1.5
F22B8.1	T05E12.2
F22B8.3	T05E12.6
F22B8.5	ZK218.1
F22B8.6	ZK218.3
F44G3.2	ZK218.5
F44G3.5	ZK218.7
F44G3.11	ZK218.11
F44G3.1	Y102A5D.1
F44G3.9	F40D4.13
F44G3.7	F40D4.12
F10A3.9	F40D4.7
F10A3.8	F40D4.6
F10A3.7	Y68A4A.2
F10A3.6	Y68A4A.5
F10A3.5	T19C9.1
F10A3.12	T19C9.6
F10A3.13	Y68A4B.3
F10A3.15	K10G4.3
K05D4.4	K10G4.1
K05D4.2	K10G4.4
K05D4.6	K10G4.5
F11A5.1	Y61B8B.2
F11A5.2	F31E9.6
F11A5.3	F31E9.5
F11A5.4	F31E9.1

F11A5.5
F11A5.7
F11A5.8
F11A5.9
F11A5.10
F21H7.5
F21H7.7
F21H7.11
F21H7.9
T13F3.2
T13F3.3
T13F3.1
F28G4.1
F28G4.5
C31A11.5
C31A11.6
C31A11.7
C31A11.9
F57G8.1
F57G8.8
F57G8.3
F57G8.4
F57G8.5
F13A7.3
F13A7.1
F13A7.8
F13A7.10
F13A7.2
F26D2.2
F26D2.4
F26D2.7
F26D2.9
F26D2.11
F26D2.1
F26D2.12
T08G3.4
T08G3.5
T08G3.2
T08G3.1
T08G3.3
T08G3.7
T08G3.10
F57E7.3
Y32B12A.1
Y32B12A.3
F36D3.6
F36D3.5
F36D3.4
F36D3.10
F36D3.9
Y32B12B.6
Y32B12B.5
F31E9.3
F31E9.4
F47H4.4
F47H4.6
F47H4.7
F47H4.8
F47H4.11
Y20C6A.2
Y20C6A.3
T27C5.2
T27C5.8
F20E11.1
F20E11.5
F20E11.6
F08E10.2
K03D7.9
K03D7.8
K03D7.7
C18D4.1
C18D4.9
C18D4.4
C18D4.3
C18D4.6a
C18D4.6b
C18D4.8
C38D9.7
C38D9.6
C38D9.1
C38D9.4
Y6G8.1
F57G4.1
F57G4.2
F57G4.4
F57G4.5
F57G4.6
F57G4.9
F57G4.8
F59A1.7
F59A1.10
F59A1.11
F59A1.12
F59A1.6
Y26G10.1
W06D12.6
W06D12.1

T03E6.1
T03E6.3
T03E6.2
T03E6.4
T03E6.6
T03E6.5
T03E6.7
Y32B12C.1
Y32B12C.2
W06G6.3
F14F8.9
F14F8.1
F14F8.6
F14F8.7
F14F8.5
Y70C5C.1
Y70C5C.2
Y70C5C.3
Y70C5C.4
T25E12.10
T25E12.8
T25E12.9
T25E12.7
T25E12.5
T25E12.4a
W06H3.2
Y102A5A.1
T20B3.1
T20B3.5
T20B3.4
T20B3.3
T20B3.7
T20B3.13
T20B3.12
Y102A5B.2
F49A5.2
F49A5.3
F49A5.4
F49A5.7
F49A5.8
F09C6.5
F09C6.4
F09C6.7
F09C6.8
Y102A5C.7
Y102A5C.8
Y102A5C.14
Y102A5C.15
Y102A5C.18
Y102A5C.21
Y102A5C.22
Y102A5C.24

Y102A5C.28
Y102A5C.29
Y102A5C.31
Y102A5C.32
F49H6.1
F49H6.2
F49H6.4
F49H6.5
F49H6.7
F28B1.6
T05E12.1
T05E12.3
T05E12.4
ZK218.6
F40D4.11
F40D4.8
F40D4.5
F40D4.1
F40D4.2
F40D4.3
Y68A4A.3
Y68A4A.6
Y68A4A.9
Y68A4A.7
T19C9.4
T19C9.3
T19C9.2
Y68A4B.2
Y68A4B.1
Y61B8A.1
Y61B8A.2
K10G4.2
Y61B8B.1
F31E9.2
F47H4.1
T27C5.1
T27C5.5
F20E11.2
F20E11.12
F20E11.10
F20E11.4
F20E11.7
F08E10.1
F08E10.6
F08E10.7
K03D7.6
K03D7.4
K03D7.2
K03D7.11
C18D4.2
C38D9.8
C38D9.2

C38D9.3
Y6G8.3
F59A1.9
F59A1.4
F59A1.3
F59A1.13
Y26G10.2
W06D12.7
W06D12.5
W06D12.4
W06D12.3
W06D12.2
R12G8.1
C47A10.1
C47A10.2
C47A10.3
C47A10.4
